# Role of chemokine systems in cancer and inflammatory diseases

**DOI:** 10.1002/mco2.147

**Published:** 2022-06-08

**Authors:** Hongyi Li, Min Wu, Xia Zhao

**Affiliations:** ^1^ Department of Gynecology and Obstetrics, Development and Related Disease of Women and Children Key Laboratory of Sichuan Province, Key Laboratory of Birth Defects and Related Diseases of Women and Children, Ministry of Education West China Second Hospital Sichuan University Chengdu China; ^2^ Department of Biomedical Sciences, School of Medicine and Health Sciences University of North Dakota Grand Forks North Dakota USA

**Keywords:** cancer progression, chemokine, chemokine receptor, inflammatory diseases, tumor microenvironment (TME)

## Abstract

Chemokines are a large family of small secreted proteins that have fundamental roles in organ development, normal physiology, and immune responses upon binding to their corresponding receptors. The primary functions of chemokines are to coordinate and recruit immune cells to and from tissues and to participate in regulating interactions between immune cells. In addition to the generally recognized antimicrobial immunity, the chemokine/chemokine receptor axis also exerts a tumorigenic function in many different cancer models and is involved in the formation of immunosuppressive and protective tumor microenvironment (TME), making them potential prognostic markers for various hematologic and solid tumors. In fact, apart from its vital role in tumors, almost all inflammatory diseases involve chemokines and their receptors in one way or another. Modulating the expression of chemokines and/or their corresponding receptors on tumor cells or immune cells provides the basis for the exploitation of new drugs for clinical evaluation in the treatment of related diseases. Here, we summarize recent advances of chemokine systems in protumor and antitumor immune responses and discuss the prevailing understanding of how the chemokine system operates in inflammatory diseases. In this review, we also emphatically highlight the complexity of the chemokine system and explore its potential to guide the treatment of cancer and inflammatory diseases.

## Graphical abstract

Chemokines are a large family of small secreted proteins that coordinate and recruit immune cells into and out of tissues and to participate in regulating the interactions between immune cells. The chemokine/chemokine receptor axis is involved in the progression of multiple malignancy types and almost all inflammatory diseases. This review summarizes recent advances of chemokine system in antitumor and protumor immune responses and discuss the prevailing understanding of how the chemokine system operates in inflammatory diseases. Modulating the expression of chemokines and/or their corresponding receptors on tumor cells or immune cells provides the basis for the exploitation of new drugs for clinical evaluation in the treatment of related diseases.

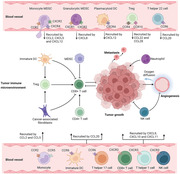

## 1 INTRODUCTION

Chemokines (chemotactic cytokines or chemoattractant cytokines) are a complicated family of small secreted proteins that, when bound to the corresponding cell surface receptors, have a fundamental role in the development of human organs, physiological function, and homeostasis of the immune system. All chemokines are 8–12 kDa peptides that modulate cellular chemotactic migration, adhesion, localization, and cell–cell interactions through binding to the so‐called classical seven transmembrane receptors, that is, G protein‐coupled receptors (GPCRs).^1^, ^2^ Chemokines can also bind to atypical chemokine receptors (ACKRs) with high affinity, which are non‐G protein‐coupled seven‐transmembrane receptors that do not induce directional cell migration. Indeed, ACKRs mainly activate β‐arrestin‐dependent pathways that regulate the bioavailability of chemokines and modulate the expression of other typical chemokine receptors or downstream signaling pathways, thus playing a role in immune responses.^3^ Chemokines possess a spectrum of characteristics and functions. Some chemokines are secreted without stimulation, which are called homeostatic chemokines and govern the chemotaxis of immune cells during immune surveillance, such as the induction of lymphocytes to lymph nodes. One function of the homeostatic chemokines is monitoring pathogen invasion by interacting with antigen‐presenting cells in tissues.^4^, ^5^ Other chemokines stimulate the formation of new blood vessels (angiogenesis), facilitate cells into tissues, and deliver specific signals for cell maturation, playing a major role in development.^6^ There are also chemokines that can be released by various cells in response to viral or bacterial infections; such chemokines can also be produced in response to noninfectious stimuli, such as inhalation of silica and the presence of urinary stones, and thus exert important functions in the inflammatory response.^7^ This group of inflammatory chemokines is released by many different types of cells and constitutes the bulk of the chemokine family. Inflammatory chemokines primarily act as chemotactic agents for leukocytes (e.g., monocytes and neutrophils), attracting leukocytes from the circulation to the site of infection or tissue damage, and activating cells to illicit an immune response or promoting wound healing.^8^ Owing to their molecular stability and targeting specificity, chemokines are thought to be critical for leukocyte infiltration and subsequent elicitation of inflammatory responses, directing the natural and adaptive immune response of the host.^7^

Given the diversity and dynamic regulation of chemokine ligand and chemokine receptor expression by tumor cells, stromal cells, and immune cells, the role of chemokines in tumor immunity is multifaceted. The tumor microenvironment (TME) is formed by cancer cells, tissue‐resident cells (like fibroblasts, endothelial cells), and infiltrating immune cells that express multiple chemokines and chemokine receptors.^9^, ^10^, ^11^ In TME, chemokines act on tumor cells to regulate their proliferative, invasive, and stem cell properties; and in turn, chemokines generated by tumor cells appeal to leukocyte infiltration, regulate neurogenesis and fibrogenesis, and induce vascularization, thereby affecting the microenvironment.^12^ However, the role of chemokines in regulating key aspects of immune cell activation, localized recruitment, phenotypic differentiation, and function within the TME during tumorigenesis is only beginning to be discovered.^13^ To complicate matters further, the same chemokine system contributes to both protumor and antitumor immune responses. The stage of disease onset, the activation status of immune cells, and the expression of chemokine receptors on regulatory and effector target cells may all have an impact on the balance between the different functions.^13^, ^14^ Further studies of chemokine systems in malignant tumors will not only provide a better understanding of cancer biology, but more importantly will also suggest novel therapeutic strategies for cancer immunotherapy. The regulation of the immune system by chemokines and chemokine receptors is also involved in a variety of inflammatory diseases other than tumors, such as rheumatoid arthritis (RA), multiple sclerosis (MS), asthma, type 2 diabetes, and atherosclerosis.^15^, ^16^

We will attempt to make a brief summary of the classification and structure of chemokines, highlighting recent advances in the function of the eight most currently reported chemokine axes in cancer progression and cancer immune response. We also summarize the latest understanding of how these chemokine systems operate in inflammatory diseases. We focus on the intricacy of the chemokine system and explore its potential to guide the treatment of cancer and inflammation‐related diseases.

## 2 CHEMOKINE SYSTEMS

### 2.1 The structure of chemokines

**FIGURE 1:**
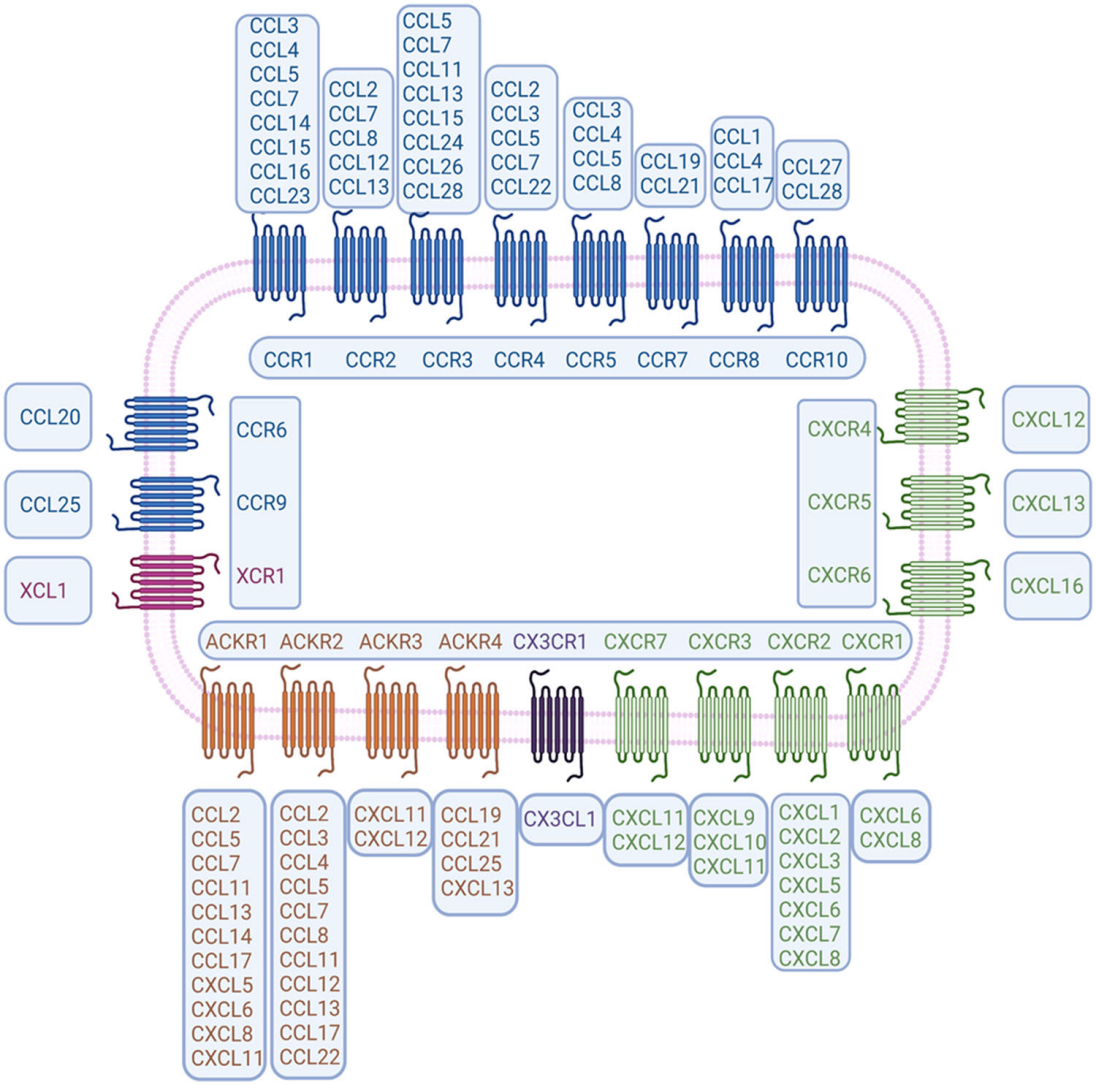
The ligand‐binding patterns of the seven‐transmembrane domain G‐protein‐coupled chemokine receptors. The receptors CCR1–CCR5, CCR7, CCR8, CCR10, CXCR1–CXCR3, and CXCR7 all bind to multiple chemokines. In contrast, CCR6, CCR9, CXCR4–CXCR6, CX3CR1, and XCR1 each bind only one ligand. Four molecules are included in the atypical chemokine receptor (ACKR) family and boast high affinity to CC‐/CXC‐ chemokines (the figure was created using biorender.com)

**FIGURE 2:**
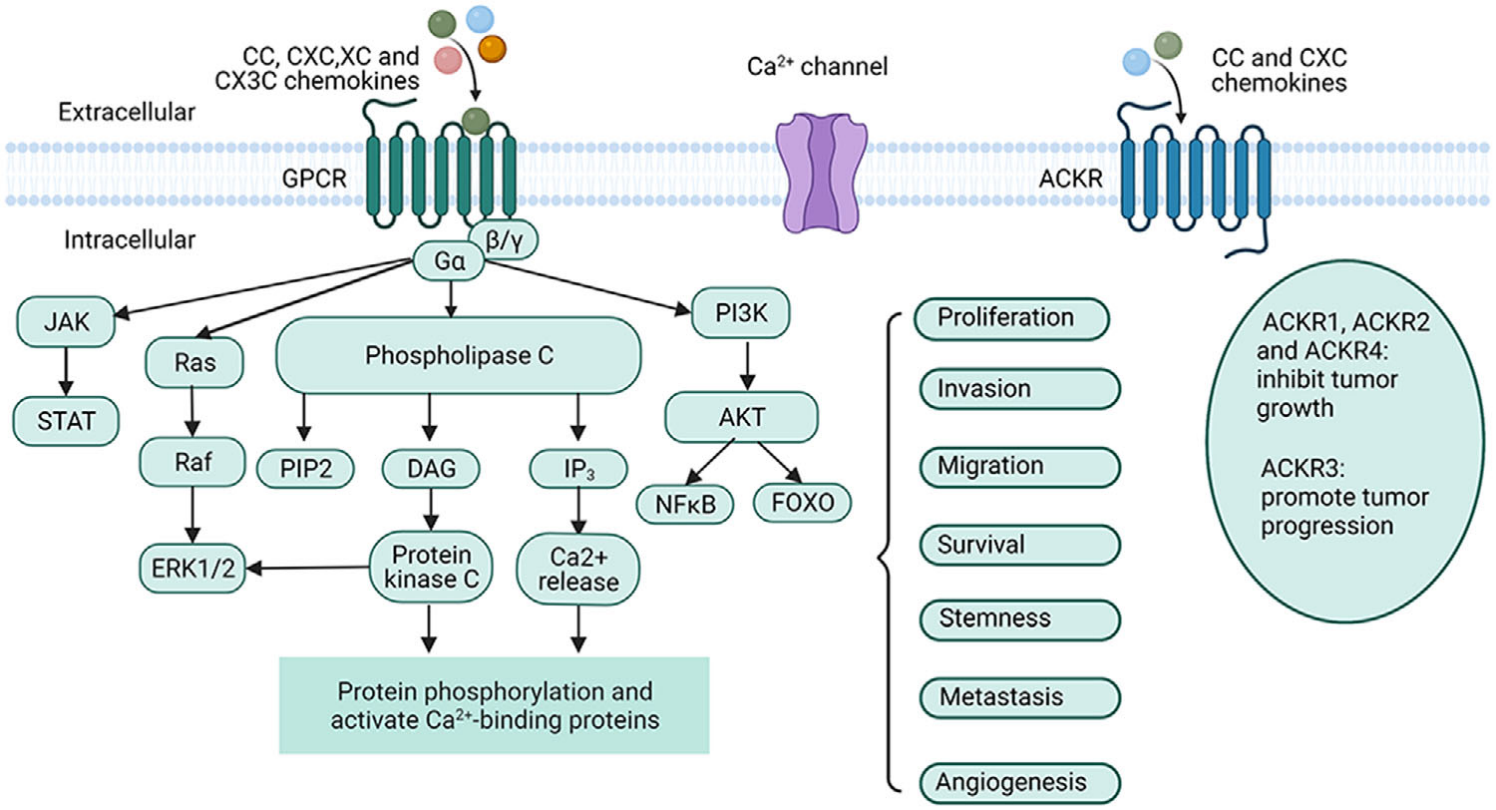
Chemokines/chemokine receptors signaling pathways. Chemokines transmit cellular signals by interacting with chemokine receptors, which are expressed on the cell surface as 7‐transmembrane proteins. Almost all types of chemokines bind to the classical G protein‐coupled receptor (GPCR), and activation of G proteins leads to subsequent activation of phospholipase C (PLC). PLC then cleaves a molecule called phosphatidylinositol‐bisphosphate (PIP2) into inositol triphosphate (IP3) and diacylglycerol (DAG); DAG activates protein kinase C, whereas IP3 triggers the intracellular release of stored calcium. Chemokines also activate the JAK/STAT, Ras/Raf/ERK, and PI3K/AKT signaling pathways through the GPCR signaling cascade. These events have an important role in cancer biology, involving tumor cell proliferation, invasion, metastasis, and angiogenesis. The atypical chemokine receptors (ACKRs) do not induce immune cell movement due to their structural inability to bind G proteins, rather their main function is to regulate the concentrations and bioavailability of chemokines on both sides of the cell membrane (the figure was created using biorender.com)

The chemokine network is composed of nearly 50 chemokine ligands, 20 GPCRs, and four ACKRs,^17^ which play significant roles in the body's immune homeostasis, inflammatory response, viral infection, and tumor progression (Figure 1). Most of the functional studies published in recent years have shown that the chemokine ligand axis conforms the classical GPCR activation paradigm.^18^, ^19^, ^20^ Once the ligand is stimulated, the G protein dissociates from the corresponding receptor and initiates different signaling events downstream of ligand binding that can eventually lead to various responses, such as cell proliferation, survival, invasion, migration, and gene transcription.^2^, ^8^, ^17^ All chemokines are small, with approximately 20–50% identical sequences between individual chemokines, suggesting homology in their gene sequences and amino acid sequences.^1^, ^21^ Typical chemokine proteins are synthesized as peptide precursors and during their secretion from cells, a signal peptide consisting of about 20 amino acids is split from the active part of the molecule. All chemokines possess conserved amino acid sequences, typically four cysteines that in most cases interact to form a Greek key shape, which is important for the formation of their three‐dimensional or tertiary structure.^22^ The first two cysteines are close to the n‐terminus of the maturing protein, the third cysteine is located in the middle of the molecule, and the fourth cysteine is near the C‐terminus. After the first two cysteines, there is a ring of about 10 amino acids called the N ring. Chemokine receptors bind to G proteins and transmit cellular signals (Figure 2). When chemokines bind to the seven transmembrane GPCRs, they initiate the dissociation of G protein subunits α and βγ, which subsequently leads to the activation of phospholipase C (PLC). PLC acts by splitting the phosphatidylinositol bisphosphate (PIP2) molecule into two second messenger elements, inositol triphosphate (IP3) and diacylglycerol (DAG). DAG then activates protein kinase C, whereas IP3 triggers the release of intracellular calcium ions, thereby driving cell polarization, adhesion, and migration. These events also trigger multiple intracellular signaling cascades (e.g., PI3K/AKT and JAK/STAT pathways) that impel activated signaling molecules into the nucleus to initiate transcriptional processes.^8^, ^17^

### 2.2 The classification of chemokines and their receptors

According to the number and location of the highly conserved N‐terminal cysteines, chemokines are grouped into four different subfamilies: CC, CXC, CX3C, XC, and the nomenclature of the receptors is essentially similar to that of corresponding chemokines, that is, CC chemokine (CCL) binds to CC chemokine receptor (CCR) and CX3C ligand binds to CX3C receptor (CX3CR) (Table 1).^23^, ^24^, ^25^, ^26^, ^27^, ^28^, ^29^, ^30^, ^31^, ^32^, ^33^, ^34^, ^35^, ^36^, ^37^, ^38^, ^39^, ^40^, ^41^, ^42^, ^43^, ^44^, ^45^, ^46^, ^47^, ^48^, ^49^, ^50^, ^51^, ^52^, ^53^, ^54^, ^55^, ^56^, ^57^, ^58^, ^59^, ^60^, ^61^, ^62^, ^63^, ^64^, ^65^, ^66^, ^67^, ^68^, ^69^, ^70^ Depending on their functions in the body, chemokines are also categorized into proinflammatory chemokines, homeostatic chemokines, or chemokines with both functions. Homeostatic chemokines, such as CCL17, CXCL14, and CXCL15, are produced constitutively in lymphocytes or other organs under normal biological conditions and are crucially important for immune surveillance because they primarily govern the homeostatic migration and homing of various immune cells.^71^ Inflammatory chemokines are induced by infection and other proinflammatory stimuli and their main function is to rapidly attract leukocytes to the site of infection or injury to act as inflammatory mediators.^71^, ^72^, ^73^ Chemokines can also be subdivided into the following categories according to the different cells on which they act. (1) monocyte/macrophage chemokines function as key chemokines to attract monocytes/macrophages to sites of inflammation, including CCL2–3, CCL5, CCL7–8, CCL13, CCL17, and CCL22.^74^, ^75^, ^76^, ^77^ (2) T lymphocyte chemokines include four chemokines implicated in the recruiting of T lymphocytes to sites of inflammation: CCL1, CCL2, CCL17, and CCL22. In addition, activated T cells induce the expression of CXCR3 and secretion of interferon (IFN)‐γ‐induced chemokines CXCL9–11 at sites of inflammation.^78^ (3) Mast cell chemokines express multiple chemokine receptors on their surface including CCR1–5, CXCR2, and CXCR4.^79^, ^80^ CCL2 and CCL5, as ligands for these receptors, play a pivotal role in the recruitment and activation of lung mast cells. (4) Eosinophil chemokines direct the migration of eosinophils to different tissues chiefly involved in several chemokines of the CC subfamily: CCL3, CCL5, CCL7, CCL11, CCL13, CCL24, and CCL26.^81^, ^82^ Eosinophils are among the first immune cells to be recruited to the lesion, where the chemokines CCL5 and CCL11 act by binding to CCR3 that is expressed on the surface of eosinophils.^83^ (5) Neutrophil chemokines are primarily CXC types of chemokines. For example, CXCL8 (interleukin [IL]‐8) in the TME is a chemotactic agent for neutrophils, inducing neutrophils into TME and activating their metabolism and degranulation.^84^, ^85^, ^86^

**TABLE 1:** The chemokine subfamilies and their receptors

Class	Systemic name	Synonym	Receptor	Function
CXC (α subfamily)	CXCL1^23^	Growth‐regulated protein‐α (GROα), Gro‐oncogene 1 (GRO1), neutrophil‐activating protein‐3, and keratinocyte‐derived chemokine	CXCR2	Inflammatory
	CXCL2^24^	Macrophage inflammatory protein‐2a (MIP‐2α), GROβ, Gro2	CXCR2	Inflammatory
	CXCL3^25^	MIP‐2β, GROγ, Gro3	CXCR2	Inflammatory
	CXCL4^26^	Platelet factor 4 (PF4)	Unknown	Unknown
	CXCL5^27^	Epithelial‐derived neutrophil‐activating peptide 78 (ENA‐78)	CXCR2 and DARC	Inflammatory
	CXCL6^28^	Granulocyte chemotactic protein‐2 (GCP‐2)	CXCR1, CXCR2	Inflammatory
	CXCL7^29^	Platelet basic protein (PBP), leukocyte‐derived growth factor (LDGF), macrophage‐derived growth factor (MDGF), Small‐inducible cytokine B7	CXCR2	Inflammatory
	CXCL8^30^	Interleukin‐8 (IL‐8), T‐cell chemotactic factor, lymphocyte derived neutrophil activating peptide (LYNAP), neutrophil activating peptide‐1 (NAP‐1)	CXCR1, CXCR2	Inflammatory
	CXCL9^31^	Monokine induced by gamma interferon (MIG)	CXCR3 and CXCR3B	Inflammatory
	CXCL10^32^	Interferon (IFN)‐γ‐induced protein 10 (IP‐10), small inducible cytokine B10	CXCR3 and CXCR3B	Dual: adaptive immunity (Th1 responses)
	CXCL11^33^	IP‐9, interferon‐inducible T‐cell α‐chemoattractant (I‐TAC)	CXCR3, CXCR3B, and CXCR7	Inflammatory
	CXCL12^34^	Stromal cell‐derived factor‐1 (SDF‐1), SDF‐1α	CXCR4 and CXCR7	Homeostatic
	CXCL13^35^	B‐lymphocyte chemoattractant (BLC)	CXCR5 and CCXCKR	Homeostatic
	CXCL14^36^	Breast‐ and kidney‐expressed chemokine	Unknown	Inflammatory: development of antigen‐presenting cells
	CXCL15^37^	Lungkine	CXCR2	Unknown
	CXCL16^38^		CXCR6	Inflammatory: T lymphopoiesis, extravasation
	CXCL17^39^	Dendritic and monocyte chemokine‐like protein, vascular endothelial growth factor (VEGF)‐coregulated chemokine‐1	Unknown	Unknown
CC (β subfamily)	CCL1^40^	Inflammatory cytokine I‐309	CCR8	Inflammatory
	CCL2^41^	Monocyte chemotactic protein‐1 (MCP‐1) and small inducible cytokine A2	CCR2	Inflammatory: innate and adaptive immunity
	CCL3^42^	MIP‐1α	CCR1 and CCR5	Inflammatory
	CCL4^43^	MIP‐1β	CCR5	Inflammatory
	CCL5^44^	Regulated on activation, normal T‐cell expressed and secreted (RANTES)	CCR1, CCR3, and CCR5	Inflammatory
	CCL6^45^	Macrophage inflammatory protein‐related protein‐1 (MRP‐1)	CCR1	Unknown
	CCL7^46^	MCP‐3	CCR1, CCR2, and CCR3	Inflammatory
	CCL8^47^, ^52^	MCP‐2	CCR1, CCR2, CCR3, and CCR5	Inflammatory
	CCL9^48^	MIP‐1γ, MRP‐2	CCR1	Inflammatory
	CCL10^49^	Unknown	Unknown	Unknown
	CCL11^50^	Eotaxin	CCR3	Inflammatory
	CCL12^51^	MCP‐5	CCR2	Inflammatory
	CCL13^52^	MCP‐4	CCR1, CCR2, CCR3, and CCR5	Inflammatory
	CCL14^53^	Hemofiltrate CC chemokine‐1 (HCC‐1)	CCR1 and CCR5	Homeostatic
	CCL15^54^	Leukotactin‐1, MIP‐5, and HCC‐2	CCR1 and CCR3	Homeostatic
	CCL16^55^	Monotactin‐1, liver‐expressed chemokine, and HCC‐4	CCR1, CCR2, CCR5, and CCR8	Homeostatic
	CCL17^56^	Thymus‐ and activation‐regulated chemokine (TARC)	CCR4	Dual
	CCL18^57^	MIP‐4, pulmonary‐ and activation‐regulated chemokine (PARC), alternative macrophage activation‐associated CC chemokine 1 (AMAC‐1)	Unknown	Homeostatic: T cell–dendritic cell interaction (spleen, lymph node)
	CCL19^58^	MIP‐3β, EBl1 ligand chemokine (ELC) exodus‐3	CCR7 and CCXCKR	Homeostatic: T lymphopoiesis
	CCL20^52^, ^59^	MIP‐3α, liver activation‐regulated chemokine (LARC), exodus‐1	CCR6	Dual: development of dendritic cells, adaptive immunity
	CCL21^60^	Secondary lymphoid tissue chemokine (SLTC), exodus‐2, TCA4	CCR7 and CCXCKR	Dual: spleen and lymph node T cell homing
	CCL22^61^	Macrophage‐derived chemokine (MDC)	CCR4	Dual: adaptive immunity (cutaneous T cells)
	CCL23^62^	MIP‐3, myeloid progenitor inhibitory factor‐1 (MPIF‐1)	CCR1	Inflammatory
	CCL24^63^	Eotaxin‐2, MPIF‐2	CCR3	Inflammatory
	CCL25^64^	Thymus‐expressed chemokine (TECK)	CCR9 and CCXCKR	Dual: T lymphopoiesis, adaptive immunity, T cell and B cell trafficking in small intestine
	CCL26^65^	MIP‐4α, eotaxin‐3, thymic stroma chemokine‐1	CCR3	Inflammatory
	CCL27^66^	Cutaneous T‐cell‐attracting chemokine (CTACK), IL‐11 receptor a‐locus chemokine (ILC), embryonic stem cell chemokine	CCR10	Homeostatic
	CCL28^67^	Mucosae‐associated epithelial chemokine (MEC)	CCR3 and CCR10	Homeostatic
XC (γ subfamily)	XCL1^68^	Lymphotactin α	XCR1	Dual
	XCL2^69^	Lymphotactin β	XCR2	Dual
CX3C (δ subfamily)	CX3CL1^70^	Fractalkine, neurotactin	CX3CR1	Inflammatory: extravasation

## 3 DIFFERENT CHEMOKINE AXES

Although a chemokine may bind to multiple receptors, when it binds to a specific receptor and exhibits excitatory effects on the binding site and antagonistic effects on other bindings, the chemokine and its receptor form a functional chemokine axis.^87^ Below, we will summarize the characteristics of eight most reported chemokine axes, as well as recent advances in these chemokine axes in cancer and inflammatory diseases.

### 3.1 The CCL2/CCR2 axis

#### 3.1.1 Brief introduction to the CCL2/CCR2 signaling axis

CCL2 belongs to the CC chemokine subfamily, also known as small inducible cytokine A2 and monocyte chemotactic protein‐1 (MCP‐1). As the most well‐studied chemokine, CCL2 was originally identified in 1989 from the culture supernatant of peripheral blood monocytes and tumor cell lines.^88^, ^89^ CCL2 was at first characterized as a “tumor‐derived chemokine” and has been documented to be a potent chemotactic agent for a couple of immune cells (e.g., monocytes, immature dendritic cells (DCs), memory T cells, and natural killer (NK) cells), thereby promoting inflammatory effects and neoangiogenesis. In addition, various stromal cells in the TME, including endothelial cells, DCs, fibroblasts, and adipocytes, are capable of producing CCL2 to promote tumor growth and progression.^90^, ^91^, ^92^ CCL2 was the first CC chemokine to be identified and fully studied and has a high affinity for its receptor, CCR2. It has been revealed that CCR2 is widely expressed in a broad range of cell types, including monocytes,^93^ endothelial cells,^94^ DCs,^95^ and various cancer cells and the upregulation of CCR2 is related to advanced cancer, metastasis, and relapse.^96^ Once bound to its ligand CCL2, the activated signaling axis triggers various intracellular G protein‐mediated signaling cascades, such as mitogen‐activated protein kinase (MAPK)/p38, PI3K/AKT, and JAK/STAT3 pathways.^97^, ^98^, ^99^ The CCL2/CCR2 axis is implied in the proliferation, invasion, and angiogenesis of tumor cells and recruitment of immunosuppressive cells.^100^, ^101^ Previous data also identified a regulatory role of the CCL2/CCR2 signaling axis on the nervous system.^102^ Microglia are the resident immune cells of the central nervous system (CNS), which is phylogenetically related to monocytes and therefore also expresses CCR2. CCL2 can also be produced by microglia/macrophages or endothelial cells under basal and neuroinflammatory conditions.^103^, ^104^ Typically, CCL2 secreted by activated astrocytes (the major glial cells of the CNS) is thought to attract microglia to sites of neuronal infection or injury, where they engulf microbes or cellular debris.^105^ The relationship between the CCL2/CCR2 signaling axis and the pathogenesis of autoimmune diseases has also been widely investigated, and a common pathological feature of these autoimmune diseases is the upregulation of CCL2 and/or CCR2 expression in the lesions.^106^, ^107^

#### 3.1.2 Roles of the CCL2/CCR2 signaling axis in tumor progression

**FIGURE 3:**
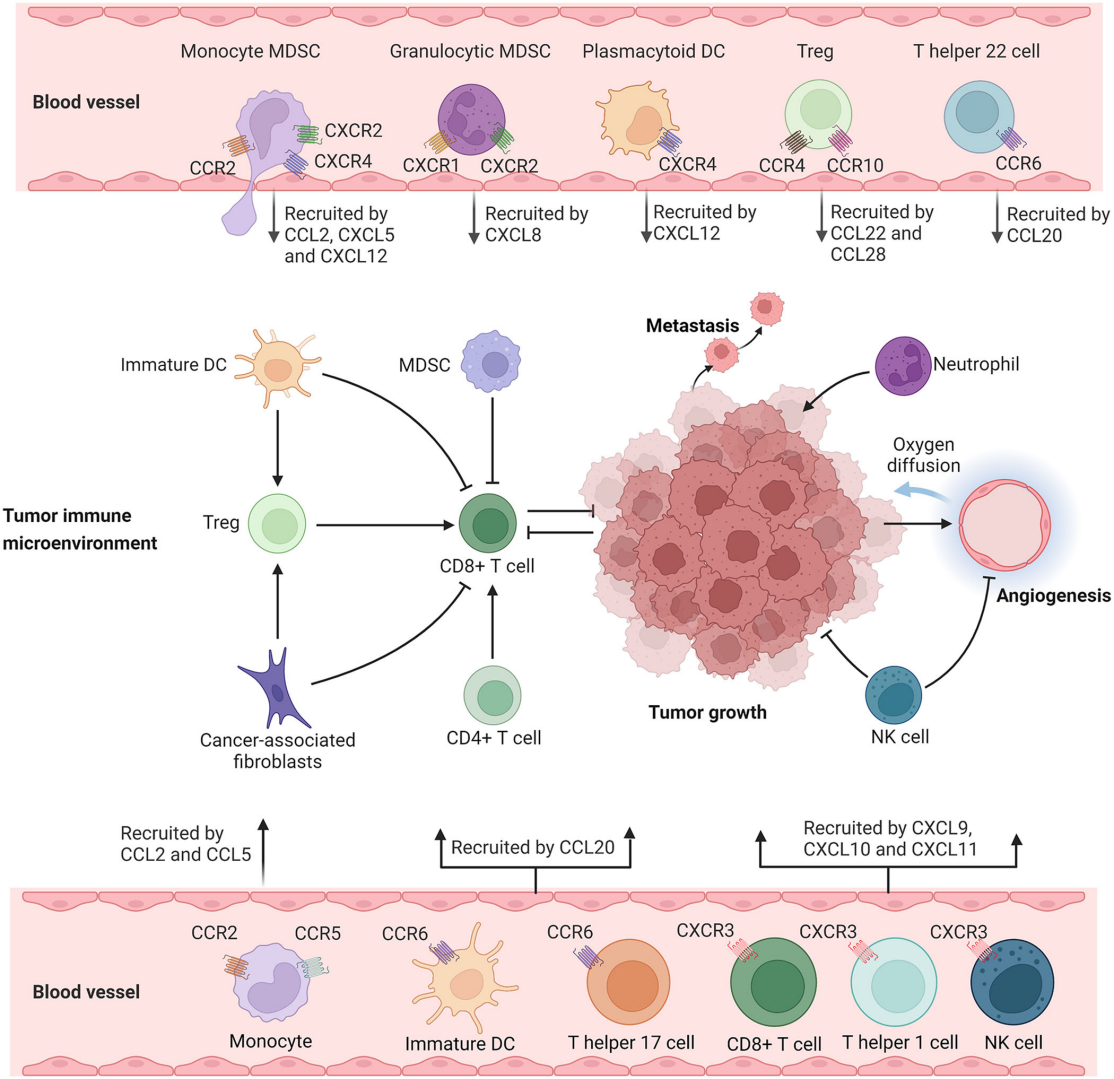
Chemokines/chemokine receptors in tumor immune microenvironment and their relevance in cancer immunotherapy. Immune cell populations, such as monocytic and granulocytic myeloid‐derived suppressor cells (MDSCs), plasmacytoid dendritic cells (DCs), regulatory T (Treg) cells, and IL‐22^+^CD4^+^ T helper 22 cells can promote tumor growth. Immune cells, such as T helper 17 cells, T helper 1 cells, CD8+ T cells, and natural killer cells (NK cells), have antitumor effects. These cells are recruited to the tumor, particularly the tumor immune microenvironment through chemokine/chemokine receptor signaling axes and are involved in almost all aspects of the tumor progression (e.g., tumor proliferation, angiogenesis, and metastasis) (the figure was created using biorender.com)

It has been demonstrated that CCL2 and its receptor CCR2 are implicated in the development and progression of various malignancies, such as prostate cancer,^108^ breast cancer,^109^ hepatocellular cancer,^110^ lung cancer,^111^ renal cancer,^112^ pancreatic cancer,^113^ and nasopharyngeal carcinoma.^114^ The CCL2/CCR2 signaling axis is involved in different stages of tumorigenic progression, for example, maintaining the proliferation and stemness of tumor cells at the site of the primary tumor; and when malignant cells metastasize, promoting the invasion of cancer cells into surrounding tissues and circulatory system, and traveling down a specific chemotactic ladder to the site of metastasis (Figure 3).^96^ After reaching a new organ and/or tissue, residual circulating tumor cells are able to successfully colonize and continue to grow through interactions with various components within the TME.^91^ It has been suggested that CCL2 might act as an autocrine or paracrine chemokine to promote the growth of tumor cells, which can be partially abolished by CCR2 antagonists or PI3K inhibitors.^115^ CCL2 can promote drug resistance in gastric cancer cells by inhibiting autophagy, and either knockdown of CCL2 or induction of autophagy successfully reversed drug resistance in tumor cells.^116^ Similarly, in vitro experiments showed that downregulation of CCL2 decreased the viability of A549 cells and enhanced docetaxel (DTX)‐induced cytotoxicity, whereas upregulation of CCL2 protected A549 cells from DTX‐induced cytotoxicity.^117^ The chemoresistance that occurs within lung cancer cells may be mediated by the stress response of CCL2‐expressing cells, implicating CCL2 as a possible target for augmenting the therapeutic efficacy of DTX on lung cancer.^117^ CCL2 also attracts different immune cells to form an immunosuppressive microenvironment, which promotes the formation of tumor‐associated microvasculature and supports the growth and metastasis of tumor cells. In mouse melanoma and pancreatic cancer models, knockdown of CCL2 with siRNA or antibody neutralization effectively inhibited DC recruitment, reduced CD68+ macrophage infiltration, and decreased tumor growth and metastasis.^118^, ^119^, ^120^ Furthermore, radiotherapy induces a significant increase in the recruitment of CCL2 and Ly6C^+^CCR2^+^ monocytes in pancreatic ductal adenocarcinoma (PDAC), thereby accelerating tumor proliferation and angiogenesis. Anti‐CCL2 antibodies selectively inhibit radiotherapy‐dependent monocyte/macrophage recruitment and retard tumor growth when used in combination with radiotherapy.^121^ However, several studies have shown inconsistent results and thereby different conclusions. Fader et al.^122^ included 37 patients with primary ovarian cancer to investigate the relationship between CCL2 expression in tumor specimens and patient response to chemotherapy and survival outcomes. The results suggested that increased expression of CCL2 in ovarian tumors was associated with better chemotherapy response and improved survival outcomes. Also, in vitro experiments illustrated that ovarian cancer cells with higher CCL2 expression were more sensitive to the traditional chemotherapeutic drugs paclitaxel and cisplatin.^122^ Interestingly, recent evidence indicates that higher levels of CCL2 in patients with squamous lung cancer is related to favorable progression‐free survival (PFS) and overall survival (OS); however, lung adenocarcinoma patients with a high expression of CCL2 exhibited a shorter OS and PFS than those with a low expression.^123^ Moreover, in vitro data from one study indicated that CCL2 could activate neutrophils and mediate the killing of breast cancer cells, whereas in mice breast cancer models, intranasal administration of CCL2 protein was able to increase the recruitment of CD4+ T cells in the lung, favoring tumor dissemination, and metastasis to the lung.^124^

Taken together, the CCL2/CCR2 signaling axis is involved in a wide range of tumor cell activities, and regulation of CCL2 and/or CCR2 expression influences tumor progression. Deeper insight into the potential mechanisms of the CCL2/CCR2 axis in tumor progression and treatment will provide new directions for a better understanding of malignancies.

#### 3.1.3 The CCL2/CCR2 axis in autoimmune diseases and neurological disorders

The pathology of autoimmune diseases is characterized by the infiltration of multiple lymphocytes in the tissues, which leads to inflammation and tissue damage. This process involves a complex network of immune cells in which chemokines act as signaling bridges. Many studies have elucidated monocytes as the molecular basis of immune cells recruitment in autoimmune disease, suggesting a major role for CCL2 and its cell surface receptor CCR2.^107^, ^125^, ^126^, ^127^ Psoriasis is a chronic skin disease caused by an imbalance between skin keratinocytes and infiltrating immune cells. In patients with psoriasis, keratinocytes secrete large amounts of CCL2, which, when combined with CCR2 on the surface of monocytes, can induce monocytes to differentiate into macrophages and migrate from the bloodstream to the site of inflammation.^128^ CCL2 is an emerging novel target in systemic lupus erythematosus (SLE) and lupus nephritis, in which the CCL2/CCR2 axis mediates the infiltration of macrophages and T cells into the nephron in nephritis.^129^ Moreover, a meta‐analysis covering 399 patients with lupus nephritis and 130 normal controls revealed that urinary CCL2 was markedly higher in patients with active lupus nephritis than those with inactive lupus nephritis and controls, suggesting that urinary CCL2 might serve as a biomarker for lupus nephritis.^130^ Meanwhile, in vivo experimental studies have illustrated that blocking CCL2 was effective in inhibiting the progression of proliferative lupus nephritis.^131^

Given the possibility that the CCL2/CCR2 axis may exert a range of immune regulation on the nervous system, its proinflammatory properties were subsequently thought to be a leading factor in the evolution of depression. Studies carried out by Stuart et al.^132^ have shown that the CCL2/CCR2 axis might be involved in regulating the proliferation and differentiation of neural progenitor cells, modulating the infiltration and activation of central immune cells, and influencing the secretion of proinflammatory factors (e.g., IL‐1β and IL‐8). Many scientists and histologists have argued that elevated CCL2 levels lead to increased blood–brain barrier permeability by inducing macrophage recruitment, cytokine production, and directly altering endothelial cell tight junction protein expression, which are observed in a variety of pathological processes, such as neuroinflammation,^133^ stroke,^134^ MS,^135^ and Alzheimer's disease (AD).^136^

The infection of HIV causes chronic inflammation in the body, along with a dysregulated immune system, which further exacerbates the inflammatory response.^137^, ^138^ The interaction of chemokine receptors (for example, CCR2, CCR3, CCR5, and CXCR4) on the cell surface with external components of HIV and accessory proteins is an essential step in HIV infection of target cells.^139^, ^140^ Among them, CCR5 and CXCR4 are the primary coreceptors for HIV‐1 invasion into target cells, with CCR2 and CCR3 playing secondary synergistic roles.^141^ In HIV infection, accentuated expression of CCL2 and/or CCR2 may contribute to HIV‐associated complications in multiple ways, depending on their role in leukocytes recruitment and maintenance of an inflammatory state.^142^ Apart from its role in inflammation and cell‐directed migration, however, CCL2 has been proven to influence directly viral replication, as evidenced by studies performed in peripheral blood mononuclear cells, T lymphocytes, and macrophages.^143^, ^144^, ^145^ Recently, several attempts have been made to investigate the relationship between high expression of CCL2 and severe acute respiratory syndrome coronavirus 2 (SARS‐CoV‐2) infection.^146^, ^147^ Conclusively, targeting the CCL2/CCR2 axis is considered to be an attractive target for the treatment of autoimmune diseases and viral infections. Therefore, more attention should be paid in the future to comprehensively investigate the potential mechanisms of action of the CCL2/CCR2 axis in these diseases mentioned above.

#### 3.1.4 Clinical trials of drugs targeting the CCL2/CCR2 axis

Due to the pivotal role played by the CCL2/CCR2 signaling axis in the progression of multiple diseases, a host of clinical trials of drugs that modulate this axis have been continually launched (Table 2). Currently, clinical trials of human neutralizing antibodies against CCL2 are focused on carlumab and MLN1202 that can effectively block the differentiation of monocytes to macrophages and reduce immune cell recruitment after binding to CCL2, exhibiting broad anti‐tumor effects. In a phase I study evaluating the safety and antitumor activity of carlumab in advanced solid tumors (NCT00537368), 44 subjects were enrolled. Carlumab was shown to be well tolerated by all patients, as no carlumab‐related adverse events were observed. However, durable stable disease (SD) was observed in only four of 33 evaluable patients, and none achieved an objective response (OR).^148^ In another completed phase II study of carlumab monotherapy for metastatic castration‐resistant prostate cancer (NCT00992186), none had an OR to carlumab treatment and only 34% of patients maintained SD for over 3 months. Notably, no patient generated antibodies to carlumab, although inhibition of free CCL2 serum concentrations was observed transiently after each dose, which seems unsustainable.^149^ Similarly, in an open‐label, multicenter phase 1b study of carlumab in combination with chemotherapy for advanced solid tumors (NCT01204996), researchers found that carlumab could be safely used in combination with standard chemotherapy doses at 10 or 15 mg/kg and was well tolerated, but did not suppress serum CCL2 levels or produce a significant antitumor response over time,^150^ suggesting that effects are not apparent and further clarification is a necessity. Meanwhile, the efficacy of MLN1202 in 44 patients with bone metastases was studied in a phase II clinical trial (NCT01015560). Of the 41 patients who completed treatment, 7.14% experienced serious adverse effects and fourteen patients experienced a decrease in urinary N‐terminal peptide values (an indicator of efficacy assessment), indicating a positive antitumor metastatic effect of MLN1202. MLN1202 was also used in randomized, double‐blind, placebo‐controlled studies of atherosclerotic cardiovascular disease and RA. The results showed that MLN1202 treatment was well tolerated and resulted in a significant reduction of high‐sensitivity C‐reactive protein levels in serum,^151^ but did not lead to amelioration of synovial inflammation in active RA.^152^

**TABLE 2:** Overview of clinical trials targeting chemokines and their receptors

Target	Drug name	Conditions	Phase	Status	Trial number
CCL2	Carlumab	Metastatic castrate‐resistant prostate cancer	II	Completed	NCT00992186
	Carlumab	Solid tumors	I	Completed	NCT00537368
	Carlumab	Combination with chemotherapy in patients with solid tumors	I	Completed	NCT01204996
CCR2	MLN1202	Cancer with bone metastases	II	Completed	NCT01015560
	MK‐0812	Multiple sclerosis	Ⅱ	Terminated	NCT00239655
	MK‐0812	Rheumatoid arthritis	II	Completed	NCT00542022
	CCX872‐B	Pancreatic adenocarcinoma	I	Not recruiting	NCT02345408
	JNJ‐41443532	Type 2 diabetes mellitus	II	Completed	NCT01230749
	JNJ‐17166864	Allergic rhinitis	II	Completed	NCT00604123
	AZD2423	Chronic obstructive pulmonary disease	II	Completed	NCT01215279
	AZD2423	Chronic obstructive pulmonary disease	II	Completed	NCT01153321
	AZD2423	Painful diabetic polyneuropathy	II	Completed	NCT01201317
	AZD2423	Posttraumatic neuralgia	II	Completed	NCT01200524
	CCX872‐B	Combination with preoperative radiation therapy in pancreatic adenocarcinoma	I /Ⅱ	Withdrawn	NCT03778879
	PF‐04136309	Advanced pancreatic adenocarcinoma	I	Completed	NCT01413022
	PF‐04136309	Combination with gemcitabine and nab‐paclitaxel in metastatic pancreatic adenocarcinoma	Ⅱ	Terminated	NCT02732938
CCR5	Maraviroc	Kaposi's sarcoma	Ⅱ	Completed	NCT01276236
	Maraviroc	Hematologic malignancy	Ⅱ	Completed	NCT01785810
	Maraviroc	Colorectal cancer	I	Completed	NCT01736813
	Maraviroc	Combination with pembrolizumab in metastatic colorectal cancer	I	Completed	NCT03274804
	Maraviroc	Graft‐versus‐host disease	I/II	Completed	NCT00948753
	Maraviroc	Hypertriglyceridemia	I	Completed	NCT01133210
	Maraviroc	Combination with rehabilitation therapy in stroke	II/III	Recruiting	NCT03172026
	Maraviroc	COVID‐19	I	Completed	NCT04435522
	Vicriviroc	Combination with pembrolizumab in colorectal neoplasms	Ⅱ	Active, not recruiting	NCT03631407
	Vicriviroc	HIV infections, acquired immunodeficiency syndrome	III	Withdrawn	NCT00243568
	Leronlimab	Solid tumors	III	Completed	NCT04504942
	Leronlimab	Nonalcoholic steatohepatitis	II	Recruiting	NCT04521114
	Leronlimab	COVID‐19	II	Recruiting	NCT04347239
	Leronlimab	Combination with carboplatin in CCR5+ triple negative breast neoplasm	I/II	Recruiting	NCT03838367
	AZD5672	Rheumatoid arthritis	II	Completed	NCT00713544
	Lentivirus vector rHIV7‐shI‐TAR‐CCR5RZ‐transduced hematopoietic progenitor cells	Intermediate‐grade or high‐grade AIDS‐related lymphoma	I	Completed	NCT00569985
CCR2/ CCR5	BMS‐813160	Combination with GVAX for locally advanced pancreatic ductal adenocarcinoma (PDAC)	I/Ⅱ	Recruiting	NCT03767582
	BMS‐813160	Combination with nivolumab, gemcitabine, and nab‐paclitaxel in borderline resectable and locally advanced PDAC	I/Ⅱ	Recruiting	NCT03496662
	BMS‐813160	Combination with chemotherapy or nivolumab in patients with pancreatic cancer	I/Ⅱ	Not recruiting	NCT03184870
	BMS‐813160	Advanced renal cell carcinoma	Ⅱ	Recruiting	NCT02996110
	BMS‐813160	Hepatocellular carcinoma	Ⅱ	Recruiting	NCT04123379
	Cenicriviroc	COVID‐19	Ⅱ	Recruiting	NCT04500418
	Cenicriviroc	Nonalcoholic steatohepatitis	II	Completed	NCT03517540
	Cenicriviroc	Nonalcoholic steatohepatitis	II	Completed	NCT02217475
	Cenicriviroc	Liver insufficiency	I	Completed	NCT02120547
	Cenicriviroc	Prediabetic state, nonalcoholic fatty liver disease, type 2 diabetes mellitus	II	Completed	NCT02330549
	Cenicriviroc	Primary sclerosing cholangitis	II	Completed	NCT02653625
	Cenicriviroc	Hepatic impairment	I	Completed	NCT03376841
CCL21	CCL21 protein	Combination with GM.CD40L vaccine in stage IV lung adenocarcinoma	I/II	Completed	NCT01433172
	CCL21‐Gene‐modified dendritic cell vaccine	Combination with pembrolizumab stage IV nonsmall cell lung cancer	I	Recruiting	NCT03546361
	Autologous dendritic cell‐adenovirus CCL21 vaccine	Melanoma (Skin)	I	Completed	NCT00798629
	Autologous dendritic cell‐adenovirus CCL21 vaccine	Advanced or recurrent nonsmall cell lung cancer	I	Completed	NCT00601094
CCR7	CD4 + CCR7 +T lymphocytes	Acute myeloblastic leukemia	Not Applicable	Completed	NCT03280290
	JBH492	Relapsed/refractory chronic lymphocytic leukemia and non‐Hodgkin's lymphoma	I/Ib	Recruiting	NCT04240704
CCL20	GSK3050002	Ulcerative colitis	I	Completed	NCT01984047
	AZD0284	Plaque psoriasis vulgaris	I	Terminated	NCT03310320
CXCL5	Immunohistochemical expression of CXCL5	Urinary bladder urothelial carcinoma	Not Applicable	Recruiting	NCT05139134
	Sunitinib malate	Kidney cancer	Not Applicable	Completed	NCT00943839
CXCR2	AZD5069	Neutrophil number and function study	I	Completed	NCT01480739
	AZD5069	Combination with enzalutamide in metastatic castration resistant prostate cancer	I/II	Recruiting	NCT03177187
	Navarixin	Combination with pembrolizumab advanced/metastatic solid tumors	II	Completed	NCT03473925
	Navarixin	Psoriasis	II	Completed	NCT00684593
	Navarixin	Allergen‐induced asthma	II	Completed	NCT00688467
	Navarixin	Chronic obstructive pulmonary disease	II	Terminated	NCT01006616
	CXCR2 ligands/CXCR2	Biological axis in pancreatic cancer	Not Applicable	Completed	NCT00851955
	RIST4721	Inflammatory response	I	Completed	NCT04105959
	CXCR2‐transduced autologous tumor infiltrating lymphocytes	Metastatic melanoma	I/II	Active, not recruiting	NCT01740557
	GSK1325756	Chronic obstructive pulmonary disease	I	Completed	NCT01209052
	GSK1325756	Nutritional status	I	Completed	NCT01209104
	Danirixin	Chronic obstructive pulmonary disease	I	Completed	NCT03136380
	Danirixin	Chronic obstructive pulmonary disease	II	Terminated	NCT03250689
	Danirixin	Chronic obstructive pulmonary disease	II	Completed	NCT03034967
	Danirixin	Infections, respiratory syncytial virus	I	Completed	NCT02201303
	Danirixin	Chronic obstructive pulmonary disease	II	Completed	NCT02130193
	SB‐656933‐AAA	Chronic obstructive pulmonary disease	I	Completed	NCT00504439
CXCL9	Emapalumab	Hemophagocytic lymphohistiocytoses	II/III	Active, not recruiting	NCT03985423
	Emapalumab	Graft failure	II	Recruiting	NCT04731298
CXCL10	NI‐0801	Primary biliary cirrhosis	II	Terminated	NCT01430429
	MDX‐1100	Combination with methotrexate in rheumatoid arthritis	II	Completed	NCT01017367
	MDX‐1100	Ulcerative colitis	I	Completed	NCT00295282
	MDX‐1100	Ulcerative colitis	II	Completed	NCT00656890
	CXCL10 protein	COVID‐19	Not Applicable	Completed	NCT04389645
CXCL10/CXCR3	Ozone	Environmental and genetic factors on lung function	Early Phase I	Recruiting	NCT03599206
CXCL12	JVS‐100	Peripheral arterial disease	II	Unknown	NCT02544204
	JVS‐100	Critical limb ischemia	II	Completed	NCT01410331
	ACRX‐100	Heart failure	I	Completed	NCT01082094
	NOX‐A12	Combination with irradiation in glioblastoma	I/II	Recruiting	NCT04121455
	NOX‐A12	Combination with pembrolizumab in colorectal and pancreatic cancer	I/II	Completed	NCT03168139
	NOX‐A12	Combination with bortezomib and dexamethasone in relapsed multiple myeloma	II	Completed	NCT01521533
	NOX‐A12	Combination with bendamustine and rituximab in relapsed chronic lymphocytic leukemia	II	Completed	NCT01486797
	NOX‐A12	Hematopoietic stem cell transplantation	I	Completed	NCT01194934
CXCR4	AMD3100	Healthy volunteers	I	Completed	NCT00322127
	AMD3100	Neutropenia	I	Completed	NCT01058993
	AMD3100	Acute myeloid leukemia	I/II	Completed	NCT00512252
	AMD3100	Lymphoma	I/II	Completed	NCT00733824
	AMD070	HIV Infections	I	Completed	NCT00063804
	POL6326	Healthy volunteers	I	Completed	NCT01841476
	CXCR4 modified anti‐BCMA (B‐cell maturation antigen) CAR T cells	Multiple myeloma	Early Phase I	Not yet recruiting	NCT04727008
	POL6326	Large reperfused ST‐elevation myocardial infarction	II	Completed	NCT01905475
	BL‐8040	Chronic myeloid leukemia	I/II	Withdrawn	NCT02115672
	BKT140	Multiple myeloma	I/II	Completed	NCT01010880
	Autologous CD4 T‐cells	HIV infections	Early Phase I	Completed	NCT03020524
	AMD11070	HIV infections	I/II	Completed	NCT00089466
	AC220	Acute myeloid leukaemia, high risk myelodysplastic syndrome	I/II	Completed	NCT01236144
	BL‐8040	Combination with pembrolizumab in metastatic pancreatic adenocarcinoma	II	Active, not recruiting	NCT02907099
	ALX‐0651	Healthy volunteers	I	Terminated	NCT01374503
	BMS‐936564	Multiple myeloma	I	Completed	NCT01359657
	BMS‐936564	Acute myelogenous leukemia and selected B‐cell cancers	I	Completed	NCT01120457
	LY2510924	Solid tumor	I	Terminated	NCT02737072
	USL311	Solid tumors, relapsed/recurrent glioblastoma multiforme	I/II	Terminated	NCT02765165
CXCL12/CXCR4	Tipifarnib	Relapsed or refractory peripheral T‐cell lymphoma	II	Completed	NCT02464228
CXCR5	CXCR5 modified EGFR CAR‐T cells	Nonsmall cell lung cancer	Early phase I	Recruiting	NCT05060796
	SP01A	HIV infections	I/II	Completed	NCT00299338

Data from https://clinicaltrials.gov/.

Inhibitors against CCR2 are designed to disrupt the binding of CCR2 to its ligand CCL2, acting by blocking the activation of a series of signaling cascades downstream of the CCL2/CCR2 axis. A phase I study evaluated the CCR2 inhibitor PF‐04136309 in combination with FOLFIRINOX for the treatment of patients with advanced pancreatic cancer (NCT01413022). Among patients who completed a course of combination therapy, 97% achieved local tumor control and 49% achieved an objective tumor response. More importantly, the mean percentage of CCR2^+^ monocytes in the blood of the combination‐treated patients was significantly lower compared with the chemotherapy group, suggesting that chemotherapy plus PF04136309 prevents the drainage of CCR2^+^ monocytes into the peripheral circulation from the bone marrow and affects antitumor immunity.^153^ Both CCX140‐B and CCX872‐B can selectively inhibit CCR2 and are mainly used to evaluate the effect on type 2 diabetes.^154^, ^155^ Several other CCR2 inhibitors (e.g., MK‐0812, JNJ‐41443532, JNJ‐17166864, AZD2423, etc.) have also been studied in clinical trials for CCL2/CCR2 axis‐related diseases, showing a favorable safety profile.^113^, ^154^, ^156^, ^157^, ^158^, ^159^

### 3.2 The CCL5/CCR5 axis

#### 3.2.1 Characteristics of the CCL5/CCR5 signaling axis

When CCL5 (also known as RANTES: regulated upon activation normal T cell expressed and secreted) was first identified, it appeared to be a classic chemokine because of its ability to direct leukocytes to sites of inflammation. It has been well accepted that CCL5 can be secreted by most inflammatory cells, with monocytes and T cells being the most common sources of CCL5.^160^ Although CCL5 can bind to several receptors, such as CCR1, CCR3–5, CD44, and GPR75, it has the highest affinity to CCR5.^161^ The transcription of CCR5 (also known as CD195) is regulated by CREB‐1 and mRNA and protein expression are commonly observed in T‐lymphocytes, monocytes, macrophages, immature DCs, eosinophils, and microglia.^162^, ^163^ CCL5 is not the only ligand for CCR5 since CCR5 also binds to proteins with an N‐terminal extracellular tail, such as CCL3 (macrophage inflammatory protein [MIP]‐1α) and CCL4 (MIP‐1β).^163^ Furthermore, CCR5 on the surface of CD4^+^ T cells is the most prominent coreceptor that assists HIV‐1 to infect cells through binding to GP120, therefore it is considered a prospective candidate for anti‐HIV therapies.^164^ The CCL5/CCR5 axis has also been reported to be engaged in the activation of several signaling pathways, including JAK/STAT, PI3K/AKT/mTOR, HIF‐α, TGF‐β‐smad, and NF‐κB axes that are implicated in inflammation, angiogenesis, tumor cell proliferation, apoptosis, and metastasis.^165^, ^166^, ^167^ In the TME, increased CCR5 levels may be the result of high CCR5 expression on the tumor cell surface or the aggregation of CCR5^+^ cells, such as monocytes, lymphocytes, adipocytes, and mesenchymal stem cells (MSCs).^161^

#### 3.2.2 The role of CCL5/CCR5 axis in cancer progression

The overexpression of CCL5 and/or its receptor CCR5 in various tumor cells (e.g. breast cancer,^168^ acute lymphocytic leukemia,^169^ multiple myeloma (MM),^170^ Hodgkin lymphoma,^171^ colorectal carcinoma^172^) has long been elucidated. The expression of CCL5 is rare in normal ductal epithelium or benign breast tumor masses, but can be obtained during malignant transformation of cells.^173^ Additionally, CCL5 is highly expressed in advanced triple‐negative breast cancer,^174^ whereas CCL5 is not overexpressed in breast tissue from women with benign breast disease or those who have undergone breast reduction.^168^ In vivo studies demonstrate that breast cancer cells stimulate MSCs to resecrete CCL5, which then acts in a paracrine manner on tumor cells. These researchers also found that lung metastasis and colonization of tumor cells increased after mice were given both breast cancer cells and MSCs, suggesting that CCL5 facilitates the metastatic ability of tumor cells.^175^ The CCL5/CCR5 axis also has far‐reaching impact on the progression of hematologic malignancies. For example, higher levels of CCL5 are detected in the serum of patients with acute myeloid leukemia, who have a monocytic phenotype^176^ or Fms‐like tyrosine kinase 3‐internal tandem duplication mutations.^177^ Also, CCL5 secretion is increased when CD40 is cocultured with classical Hodgkin lymphoma cells or with MSCs derived from lymph nodes of Hodgkin lymphoma patients.^178^ High levels of CCL5 in Hodgkin's lymphoma tissue are correlated with monocyte infiltration and poor prognosis.^171^ In addition, in vivo studies have shown that inhibition of CCR5 by neutralizing antibodies or antagonists was able to retard the progression of MM, reduce osteolytic lesions, and inhibit osteoclastogenesis.^179^, ^180^ CCL5 and CCR5 are overexpressed in colorectal cancer (CRC) primary tumor cells as well as in metastasis cells of the liver and lung and are positively correlated with prognosis in CRC.^181^ Furthermore, the CCR5/CCL5 axis also plays a critical role in the progression of multiple solid tumors, including gastric cancer (GC),^182^ glioblastoma,^44^ head and neck cancer,^183^ lung cancer,^184^ ovarian cancer,^185^ and so on, often by promoting tumor cell proliferation, metastasis, and assisting in the establishment of immunosuppressive TME.^186^

Briefly, the above results suggest that CCL5 and CCR5 are highly expressed in various tumor cells and promote tumor proliferation and metastasis by recruiting immune cells. Based on the finding that CCR5 antibodies retard tumor progression and inhibit angiogenesis,^179^, ^180^ we speculate that CCL5/CCR5 may be a potential target in cancer therapy.

#### 3.2.3 The CCL5/CCR5 axis in inflammatory diseases

In addition to being involved in the progression of multiple tumors, aberrant CCL5/CCR5 interactions have been identified in multiple types of inflammation,^187^ including AD, atherosclerosis, diabetes, hepatitis, and some viral infections.

With the intensive study by scientists of the pathology of atherosclerosis, it is now widely accepted that the disease is an inflammatory disease in which the continuous accumulation of macrophages in the intima and the rise in cytokine levels in peripheral blood and local tissues are important hallmarks.^188^ It has been reported that the chemokine CCL5 assists in the recruitment of monocytes to the intima in the early stages of atherosclerosis and promotes the conversion of macrophages.^189^ Recently, Jongstra‐Bilen et al.^190^ showed that the expression of CCL5 mRNA as well as other ligands of the CCR5 receptor (CCL3 and CCL4) was induced in the aortic intima of Ldlr−/− mice 3 weeks after the onset of cholesterol‐rich diet‐induced hypercholesterolemia. Blockade of CCR5 significantly reduced the recruitment of monocytes at the lesion site, suggesting that CCL5 chemokine signaling through CCR5 is critical. Further, CCL5/CCR5 has been reported to attract T cells to the lesion site to release inflammatory factors (e.g. perforin‐1, IL‐6, selectin, tumor necrosis factor‐α [TNF‐α], etc.) and exacerbate inflammatory damage.^191^, ^192^ Besides, CCL5 levels were found to be elevated in damaged tissues of inflammatory bowel disease (IBD), which then induced an influx of inflammatory factors. Knockdown of the CCR5 gene reduced the recruitment and activation of CCR5(+) leukocytes in the mucosa, leading to greatly reduced symptoms of inflammation in a metastatic model of colitis. Similarly, the CCR5 inhibitor maraviroc attenuated the development of intestinal inflammation by selectively reducing the recruitment of CCR5(+) leukocytes.^193^ Upon liver injury, resident Kupffer cells interact with hepatic cell populations and release chemokines to recruit circulating leukocytes, of which monocytes subsequently differentiate into macrophages in the liver, influencing the development of tissue inflammation.^194^ It has been shown that the CCL5/CCR5 signaling pathway can accelerate the inflammatory process in the liver through the NF‐kB pathway.^195^ A new study indicated that CCL5 could directly activate M1 polarization and impede M2 polarization through CCR1‐ and CCR5‐mediated activation of MAPK and NF‐κB pathways. Neutralizing antibodies or antagonists with CCL5 could greatly reduce liver injury and improve survival in drug‐injured mouse models.^196^ Additionally, the CCL5/CCR5 axis is also involved in viral infections. COVID‐19, a pandemic currently plaguing people worldwide, is caused by SARS‐CoV‐2 infection. Recent studies have shown that inhibition of the CCL5/CCR5 axis by the monoclonal antibody leronlimab could alleviate the symptoms of patients with critical pneumonia.^197^ Also, it was observed that the levels of inflammatory molecules such as CCL5, IL‐6, and TNF‐α in the serum of patients were reduced after anti‐CCR5 treatment.^198^

#### 3.2.4 Clinical trials of drugs targeting the CCL5/CCR5 axis

We have described above the seminal function of the CCL5/CCR5 axis on tumor progression in preclinical studies, which can be attenuated by knocking down CCL5, using antibody neutralization, and CCR5 antagonists to block ligand–receptor binding, setting the stage for drugs targeting the CCL5/CCR5 axis to enter clinical trials. Table 2 summarizes clinical trials of several CCR5 antagonists (maraviroc, vicriviroc, AZD5672, and leronlimab) for the treatment of metastatic CRC, advanced breast and pancreatic ductal carcinoma, and Kaposi's sarcoma associated with HIV infection (Table 2). In addition, maraviroc has been reported for the treatment of conditions other than tumors, including graft‐versus‐host disease, hypertriglyceridemia, stroke, and COVID‐19 (NCT00948753, NCT01133210, NCT03172026, and NCT04435522). Another dual chemokine receptor antagonist, cenicriviroc, which targets CCR2/CCR5 and inhibits monocyte migration, has also being evaluated for safety and efficacy in clinical trials. Cenicriviroc also inhibits HIV‐1 and HIV‐2 infection and has potent anti‐inflammatory and anti‐infective activity.^199^, ^200^ Clinical trials related to cenicriviroc have shown a role in the treatment of COVID‐19 (NCT04500418), nonalcoholic steatohepatitis (NCT03517540), primary sclerosing cholangitis (NCT02653625), type 2 diabetes mellitus (NCT02330549), and many other diseases.

### 3.3 The CCL19/CCL21/CCR7 axis

#### 3.3.1 Brief introduction of the CCL19/CCL21/CCR7 axis

Chemokines CCL19 and CCL21 are highly expressed in lymphoid tissues and act upon binding to the same receptor CCR7. In primary lymphoid organs (e.g., thymus), CCR7^+^ thymocytes are subject to CCL19 and CCL21 chemotaxis and move between different tissue sections. In particular, the CCL19/CCL21/CCR7 axis plays an instrumental role in thymocyte differentiation, clonal selection, and negative selection.^201^ Within secondary lymphoid organs (e.g., lymph nodes), CCL19 and CCL21 are continuously secreted by high endothelial veins and fibrous reticulocytes, which bind to CCR7 and then induce lymphocytes in the circulation to enter lymphatic vessels and lymph nodes.^201^ CCR7 is mostly expressed on DCs and T cells for orchestrating immune responses.^202^, ^203^ The role of CCR7 in inducing targeted migration of lymphocytes in immune and inflammatory responses is well recognized. As the predominant class of antigen‐presenting cells, DCs can rapidly transform to a mature state upon antigen stimulation and upregulate major histocompatibility complex II and several costimulatory molecules, such as CD80, CD86, and CCR7.^204^ The elevated levels of CCR7 allow DCs to sense the CCL19/CCL21 concentration gradient, move toward the highest concentration of this signaling protein, enter the lymph nodes for antigen‐presenting and then activate T cells.^205^, ^206^ CCR7 also controls the migration of thymocyte‐derived regulatory T cells and effector T cells from sites of inflammation to the second lymphoid organ (SLO) as well as their proper localization.^207^, ^208^ CCR7 expressed on the surface of cancer cells can direct malignant cells from the primary site into the lymphatic system by binding to appropriate signaling proteins, spreading in the body and eventually forming metastases in other tissues, which greatly increases the risk of death for patients.^209^

#### 3.3.2 Roles of the CCL19/CCL21/CCR7 axis in cancer progression

Because of the pivotal role of the CCL19/CCL21/CCR7 signaling axis in both antigen presentation and activation of T cell‐mediated responses, it has been postulated that increasing the levels of CCL19 and CCL21 within tumors could aid immunotherapy of cancer by enhancing the immune response to tumors. The first of these is to increase CCL19/21 concentration within the TME to sharpen the immune response to tumor. For instance, in mouse lung and colorectal models, intratumoral injection of CCL19 protein directly was shown to result in increased DCs as well as CD4^+^ and CD8^+^ T cells in the TME, promote enhanced secretion of proinflammatory factors, such as chemokines CXCL9 and CXCL10, as well as cytokines IL‐12, granulocyte‐macrophage colony stimulating factor (GM‐CSF), and IFN‐γ, and decrease levels of immunosuppressive molecules prostaglandin E2 and transforming growth factor‐β (TGF‐β); ultimately retarding tumor growth.^210^, ^211^ Similarly, in an orthotopic mouse model of breast cancer, intratumoral administration of CCL21 significantly increased the proportion of T cells, NK cells, and DCs within the tumor, reduced the size of the tumor and extended the survival time of tumor‐bearing mice.^212^ Introduction of exogenous genes into tumor cells by recombinant plasmid techniques (e.g., lentiviral transfection) is another way to achieve elevated concentrations of CCL19/CCL21 protein. For example, mouse melanoma and ovarian cancer cells transfected respectively to express mouse CCL19 and then inoculated into C57BL/6 mice, showed significantly slower tumor growth compared with the control group without CCL19 expression.^213^ In a mouse lung metastasis model, injection of endothelial progenitor cells overexpressing CCL19 by tail vein was able to reduce the number of lung metastatic nodules and prolong the survival of mice.^214^ In addition, transfection of breast cancer MCF‐7 cells with CCL21 protein potentiated a range of functions of DCs, such as antigen uptake and presentation, migration, and antiapoptotic ability in vitro.^215^

Therapeutic strategies that increase the intratumoral CCL19 or CCL21 levels may also be utilized in combination with other immunotherapies or nonimmunotherapies to improve antitumor efficacy. DNA vaccines have been a particularly attractive approach in recent years to enhance protective antitumor immunity by mobilizing leukocytes (e.g., cytotoxic T cells and NK cells) to the tumor. When combined with CCL21, the DNA vaccine showed higher efficacy than single treatment in a mouse model of orthotopic melanoma.^216^ In addition, MSCs expressing CCL19 have been demonstrated to promote immune cell infiltration into TME and enhance the efficacy of anti‐PD‐L1 antibodies.^217^ Therefore, targeting the CCL19/CCL21/CCR7 axis to inhibit lymphatic metastasis but maintaining a robust antitumor immune response has increasingly become a bright spot in tumor immunotherapy.^209^

#### 3.3.3 The CCL19/CCL21/CCR7 signaling axis in autoimmune diseases

RA is a chronic, systemic autoimmune disease of unknown etiology, characterized by intraarticular inflammatory cell infiltration and elevated proinflammatory cytokines, which can lead to multiple joint deformities and even loss of function.^218^ Page et al.^219^ detected DC subsets within the synovium of RA patients by immunohistochemical staining and showed that immature DCs were found only in the lining layer of the synovium, whereas mature DCs were found in the perivascular lymphatic aggregation zone. In addition, the expression of CCL19, CCL21, and CCR7 was only increased in the perivascular area, suggesting that the expression of these chemokines as well as CCR7 is associated with lymphocyte aggregation. In addition, upregulation of CCL19 and CCR7 gene expression was shown in psoriasis patients, whose key role is involved in establishing the typical inducible skin‐associated lymphoid tissue structures during disease progression, which can be clearly identified in the skin aggregates at the lesion site.^220^ The majority of lymphocytes in healthy human cerebrospinal fluid (CSF) are CCR7^+^ central memory T cells,^221^ whereas in patients with relapsed and progressive MS, increased expression of CCL19 is found in CSF,^222^ implying that the CCL19/CCR7 axis may be involved in the normal immune surveillance of the brain. In experimental autoimmune encephalomyelitis (EAE) models, blocking CCR7 signaling has been proved to reduce the binding of T cells to inflammatory venues in EAE brain slices.^223^, ^224^ Collectively, these results suggest that the CCL19/CCR7 axis plays an important role in the progression of autoimmune diseases and is closely related to immune regulation at the site of the lesion. Blocking the CCL19/CCR7 axis is a potential therapeutic option for the treatment of autoimmune diseases.

#### 3.3.4 Clinical trials of drugs targeting the CCL19/CCL21/CCR7 signaling axis

As mentioned above, modulating the function of the CCL19/CCL21/CCR7 axis may hold therapeutic potential for cancer as well as many inflammation‐related diseases, and there are several clinical trials currently underway.

One of the phase I trials is studying the side effects and optimal dose of autologous DC‐adenovirus CCL21 vaccine combined with intravenous pembrolizumab and seeing how well they work in treating patients with stage IV nonsmall cell lung cancer (NCT03546361). The researchers concluded that vaccines made from genetically modified viruses may help the body build an effective immune response to kill tumor cells, whereas monoclonal antibodies, such as pembrolizumab, may interfere with the ability of tumor cells to grow and spread, so giving a CCL21 genetically modified DC vaccine with pembrolizumab to treat patients with stage IV nonsmall cell lung cancer may work better. This clinical trial enrolled 24 patients, and up to 12 patients will participate in the dose escalation phase, during which 12 patients will be evaluated during the dose expansion. Two additional phase I clinical trials investigated the safety, toxicity and maximum tolerated dose of the autologous DC‐adenovirus CCL21 vaccine. The vaccine as an intratumoral injection was well tolerated by patients with advanced or recurrent nonsmall cell lung cancer and cutaneous melanoma (NCT00601094, NCT00798629). Besides, JBH492 is an antibody–drug conjugate that consists of an antibody against CCR7 on tumor cells combined with the steroid DM4, which leads to inhibition of tumor cell proliferation. A phase I/Ib Open‐label clinical trial (NCT04240704) is currently undertaken for investigating the preliminary effects of JBH492 monotherapy on non‐Hodgkin's lymphoma and chronic lymphocytic leukemia.

### 3.4 The CCL20/CCR6 axis

#### 3.4.1 Major characteristics of the CCL20/CCR6 signaling axis

Since its discovery in the 1990s, CCL20 has gradually been attributed several names, such as MIP‐3α, liver and activation regulatory chemokine and exodus‐1, and it has gained increasing attention in molecular and cellular immunology. A very large number of cells in the body express CCL20, including CD8+ T cells, B lymphocytes, T helper 17 cells, macrophages, neutrophils, DCs, mast cells, and endothelial cells.^225^, ^226^, ^227^ As an inflammatory chemokine, CCL20 strongly attracts lymphocytes and DCs to lymphoid tissues, thus participating in the formation and function of lymphoid tissues at various sites. CCL20 also has a predominant role in innate immunity, being upregulated by transcription factors, such as NFκB,^228^ and induced by the TNF‐α and IL‐1β.^229^ When the immune system is stimulated by inflammatory substances such as lipopolysaccharide, CCL20 is also quickly upregulated, leading to a prompt accumulation of immune cells in the spleen.^230^ Currently, there is only one known receptor for CCL20, namely CCR6.^231^, ^232^ CCR6 is the hallmark chemokine receptor of immune cells. When CCL20 binds to its receptor CCR6, it not only participates in regulating the immune homeostasis of the body, but also serves to modulate the inflammatory response through the Th17 pathway, playing an essential role in the progression of autoimmune diseases and various malignant tumors.^59^, ^233^, ^234^

#### 3.4.2 The CCL20/CCR6 axis in cancer progression

With concerted, extensive efforts, the functional role played by the CCL20/CCR6 axis is gradually being unfolded, particularly in regulating cancer progression and metastasis within the TME. Ding et al.^235^ revealed that tissue expression of CCL20 in clinical specimens of hepatocellular carcinoma (HCC) was related to tumor size, differentiation, recurrence, and vascular infiltration, and that high CCL20 expression was associated with worse PFS and OS in patients. In in vitro analysis, CCL20 greatly enhanced the invasive ability of triple‐negative breast cancer cell lines by increasing the secretion of matrix metalloproteinase (MMP)‐2 and MMP‐9; meanwhile, anti‐CCL20 antibody by intraperitoneal injection in a mouse breast cancer model could effectively inhibit the occurrence of bone metastases.^236^ A study showed that serum CCL20 and IL‐17A levels were higher in CRC patients than those in healthy subjects, and the combination of CCL20 and IL‐17A signature curve analysis could differentiate CRC patients from healthy volunteers effectively.^237^ In a case study of chemotherapy resistance to the FOLFOX regimen, CCL20 secreted by tumor cells was able to facilitate Tregs recruitment into the TME, which enhanced chemoresistance and closely correlated with poorer survival rates.^238^ Additionally, IL‐4‐treated M2‐type macrophages highly express CCL20 and the CCL20/CCR6 axis promotes pancreatic cancer proliferation and distant metastasis via inducing epithelial–mesenchymal transition (EMT) in vivo.^239^ Furthermore, CCL20 and/or CCR6 proteins were found to be highly expressed in lung,^240^ cervical,^241^ gastric,^242^ ovarian cancer tissues,^243^ and renal cell carcinoma,^244^ facilitating tumor proliferation and directional migration through autocrine or paracrine modes.

#### 3.4.3 The CCL20/CCR6 axis in autoimmune diseases

Similar to the CCL19/CCL21/CCR7 axis, the CCL20/CCR6 axis serves an essential role in the recruitment of inflammatory cells in the immune response and may contribute to a variety of autoimmune diseases, such as MS, IBD, psoriasis, and RA.^234^ A study showed that stimulation of human epithelial cells with Th17 cytokines (IL‐17A, IL‐22, and TNF‐α) was able to induce a remarkable increase in CCL20 and CCR6 levels in their cultures in a dose‐ and time‐dependent manner. Similar results were obtained in in vivo tests, where subcutaneous injection of these Th17 cytokines also resulted in increased expression of CCL20 and CCR6, as well as infiltration of mature DCs and CD4^+^ T cells in the skin of mice.^245^, ^246^ In several animal models of psoriasis, the use of anti‐TNF‐α antibody infliximab,^247^ anti‐CCR6 antibody,^248^ and the anti‐CCL20 antibody,^249^ respectively, significantly reduced regional infiltration of CCR6^+^CD4^+^ T cells and attenuated the inflammatory response in affected skin lesions. Significantly elevated levels of CCL20 were also found in the affected joints of RA patients, followed by a marked increase in CD4^+^CD45RO^+^CCR6^+^ memory T cells in the peripheral circulation of the patients.^250^ Hirota et al.^251^ found that in an animal model of RA, IL‐17‐producing Th17 cells predominantly expressed CCR6 and its ligand CCL20. Blockade of proinflammatory cytokines with infliximab or anti‐IL‐6R antibodies significantly decreased the CCL20 level and Th17 cells migration to the joints.^251^ In the MS mouse model known as EAE, CCR6 and CCL20 expression was found to be upregulated in the spinal cord,^252^ and CCR6‐deficient mice showed a milder development of EAE compared with wild‐type mice.^253^ CCL20 and CCR6 also play an important contribution to the pathogenesis of IBD by regulating the delicate balance of Th17 and Tregs.^254^, ^255^ Data from these results indicate that the CCL20/CCR6 axis is implicated in the pathogenesis of autoimmune diseases and that antibodies or antagonists to CCR6 or CCL20 hold promise as an intriguing treatment tactic to ameliorate neuroinflammation and autoimmunity.

A human monoclonal antibody (MOR103) to GM‐CSF was well tolerated in randomized clinical trials and showed preliminary evidence of efficacy in RA (NCT01023256) but little improvement in the severity of MS (NCT01517282). Additional animal experiments and clinical trials are certainly needed to gain more insight into the pivotal roles of the CCL20/CCR6 axis in human disease.

### 3.5 The CXCL5/CXCR2 axis

#### 3.5.1 Basics of the CXCL5/CXCR2 signaling axis

CXCL5, previously named epithelial neutrophil‐activating peptide‐78 (ENA‐78), is characterized by its ability to recruit neutrophils during the immune response,^27^, ^256^ contribute to angiogenesis and reshape the connective tissue.^257^ CXCL5 is secreted by cells stimulated by the inflammatory cytokines IL‐1 or TNF‐α, whereas many immune cells (e.g., macrophages,^258^ eosinophils^259^) and nonimmune cells (e.g., mesothelial cells^260^ and cancer‐associated fibroblasts^261^) are also capable of expressing CXCL5. In addition, the secretion of CXCL5 and IL‐1β in TME interferes with the maturation of functional DCs, and CXCL5 expressed in eosinophils inhibits the secretion of IFN‐γ.^262^ CXCL5 activates downstream signaling pathways by binding to the IL‐8B receptor, which later became known as CXCR2 and is highly expressed on neutrophils, although CXCR2 also binds to other ligands, including CXCL1–3 and CXCL6–8.^2^

#### 3.5.2 The CXCL5/CXCR2 signaling axis in cancer progression

The relationship between the CXCL5/CXCR2 signaling axis and carcinogenesis is increasingly being recognized. Abnormal elevations of CXCL5 and/or CXCR2 proteins have been noted in as many as 14 distinct malignant tumor types, including but not limited to CRC,^263^, ^264^ nonsmall cell lung cancer,^265^ breast cancer,^266^ bladder cancer,^267^ nasopharyngeal carcinoma,^268^ and so on. The expression intensity of CXCL5 was also detected in line with the malignancy degree, metastasis, and survival of cancer patients, which provides new potential for clinical application.^27^ The CXCL5/CXCR2 signaling axis can also indirectly promote tumor progression via modulating the function of various immune cells within the TME. For instance, Zhou et al.^269^ discovered that CXCL5 might contribute to the likelihood of tumor metastasis and recurrence in a mouse intrahepatic cholangiocarcinoma model via recruiting intratumoral neutrophils. Overexpression of CXCL5 on HCC stem cell‐like cells recruits immunosuppressive neutrophils and promotes lymphatic metastasis of tumor cells through binding CXCR2.^270^ MDSCs in breast and renal cell carcinomas were shown to be related to tumor grade and positively correlated with CXCL5 expression.^271^, ^272^ In addition, a positive correlation was noted between CXCL5 expression and the number of CD8^+^ T cells, CD11b^+^MMP9^+^Ly6G^+^ granulocytes and macrophages in colorectal and pancreatic cancers.^263^, ^273^

Because of CXCL5's chemotactic effect on vascular endothelium, it is also considered to be a potent angiogenic factor. In animal studies of bladder, renal cell, and CRCs, CXCL5 secreted by tumor cells binds its receptor CXCR2 and activates the downstream AKT/NF‐*κ*B signaling pathway, thereby stimulating the proliferation and aggregation of endothelial cells.^274^, ^275^, ^276^ Data from several sources have identified the involvement of CXCL5 in the proliferation of other types of tumor cells, such as prostate cancer,^277^ lung cancer,^265^ cervical cancer,^278^ hepatoblastoma,^279^ osteosarcoma,^280^ and papillary thyroid carcinoma.^281^ In addition, the levels of CXCL5 were significantly higher in the lymph node metastatic tissue of head and neck squamous cell carcinomas than those in the primary tumor area.^282^, ^283^ CXCL5 expression was also stronger in HCC cell lines with high metastatic potential than that in their less aggressive counterparts, due to its ability to strongly activate the ERK1/2 and PI3K/AKT signaling pathways in tumor cells.^284^, ^285^

#### 3.5.3 The CXCL5/CXCR2 axis in inflammatory diseases

Due to the strong chemotactic effect of CXCL5 on neutrophils, its role in regulating the inflammatory response has been widely noted. In a study of mechanical pain sensitization caused by ultraviolet B (UVB), Dawes et al.^286^ explored changes in all chemokines and inflammatory factors in inflamed skin sites in humans and rats after UVB exposure. It was found that CXCL5 expression was most significantly elevated in the inflamed skin. Moreover, CXCL5 injection via the plantar area triggered a dose‐dependent decrease in mechanical pain threshold in rats, a result that reveals for the first time the mechanism of CXCL5 involvement in chronic inflammatory pain.^286^ In addition, Xu et al.^287^ found that the expression of CXCL5 and its receptor CXCR2 was elevated notably in spinal cord neurons of rats with chronic constriction injury of the sciatic nerve, and that the CXCL5/CXCR2 pathway regulated the phosphorylation of glycogen synthase kinase‐3 (GSK‐3β), which induced neuropathic pain in rats. Mild inflammation promotes retinal ganglion cell (RGC) survival and axonal regeneration after optic nerve (ON) injury with the involvement of infiltrating macrophages and neutrophils. Interestingly, the expression of Cxcl5 and Cxcr2 is increased when the ON and lens are injured. In retinal graft cultures, the addition of recombinant CXCL5 promoted RGC survival and neurite growth with an increase in the number of activated microglia, a phenomenon that was inhibited by the CXCR2 antagonist SB225002.^288^

In pulmonary inflammation, the polymorphonuclear neutrophils (PMNs) recruited from the blood are essential for alveolar space defense and pathogen clearance. However, when PMNs are extensively translocated into the interstitial and alveolar spaces of the lung, they can lead to uncontrolled immune responses.^289^ CXCR2 is obviously upregulated in airway epithelial cells during acute exacerbations of chronic obstructive pulmonary disease (COPD), and there is a significant positive correlation between CXCR2 expression and the number of neutrophils. Blocking CXCR2 reduced the proportion of neutrophils in bronchoalveolar lavage fluid in a mouse model.^290^ Asthma is also a chronic inflammatory disease of the lungs, and although the role of neutrophils in stable asthma is unclear, a significant increase is observed in late responses to stimulation or asthma exacerbation, accompanied by increased levels of CXCL5 and CXCR2.^291^ CXCL5 and its receptor CXCR2 were overexpressed in lung tissue of acute respiratory distress syndrome (ARDS) through the upregulation of MMP‐2 and MMP‐9.^292^ In addition, CXCL5‐neutralizing antibodies effectively attenuated the inflammatory response, diffused alveolar injury and pulmonary edema, and reduced the expression levels of MMP‐2 and MMP‐9 in ARDS mouse models.^292^ Further studies confirmed that CXCR2 is essential for the development of autoantibody‐mediated arthritis and that it upregulates the expression of the corresponding ligands CXCL1, CXCL2, and CXCL5.^293^, ^294^, ^295^

The results of these studies suggest that blocking CXCL5/CXCR2 signaling appears to be a promising strategy for a wide range of inflammatory diseases and that in‐depth studies of this pathway are warranted.

#### 3.5.4 Therapeutic strategies targeting the CXCL5/CXCR2 axis

In recent years, the CXCL5/CXCR2 axis has received increasing attention for its potential in cancer screening, tumor prognosis and personalized anticancer therapy. First, in vivo tests have shown that blocking CXCL5 or applying CXCR2 antagonists can slow disease progression by blocking the AKT/NF‐*κ*B signaling pathway, thereby effectively reducing the blood supply to the tumor.^296^ In addition, CXCL5‐neutralizing antibody‐treated mice showed reduced metastasis of breast cancer cells, mainly through inhibition of ERK/Snail signaling.^297^ Meanwhile, therapeutic strategies targeting CXCL5/CXCR2 in combination with chemotherapy or immunotherapy have being explored. For example, in a mouse lung cancer model, CXCL5 antibodies synergistically enhanced the therapeutic effect of the tyrosine kinase inhibitor gefitinib through activating the AKT/NF‐*κ*B and ERK/RSK1/2 signaling pathways.^298^ Additionally, the CXCR2 antagonist SCH‐527123 not only inhibited tumor proliferation, invasion, and angiogenesis, but also enhanced the sensitivity of CRC to oxaliplatin treatment.^296^ In a phase I clinical trial of patients with human epidermal growth factor receptor‐2 negative metastatic breast cancer, a 30% responsiveness was observed for orally administered noncompetitive CXCR1/2 antagonist reparixin adjuvant to paclitaxel, and there was no pharmacokinetic effect between the two drugs (NCT02001974). Navarixin (MK‐7123), a CXCR2/CXCR1 antagonist with oral bioavailability, is currently evaluated in clinical trials for its efficacy and safety in advanced/metastatic solid tumors, psoriasis, and COPD (NCT03473925, NCT00684593, NCT00688467, and NCT01006616). Danirixin, another selective CXCR2 antagonist with high affinity, is able to effectively inhibit the binding of CXCL8 (IL‐8) to CXCR2, and several clinical trials have focused on its improvement of lung function in patients with mild to severe COPD (NCT03136380, NCT03250689, NCT03034967, and NCT02130193).

### 3.6 The CXCL9, ‐10, ‐11/CXCR3 axis

#### 3.6.1 Introduction to the CXCL9, ‐10, ‐11/CXCR3 axis

CXCL9, ‐10, and ‐11 are selective ligands of CXCR3. These ligands are primarily produced by cancer cells, endothelial cells, fibroblasts, and monocytes, and are commonly expressed at low levels in the homeostatic state, but are upregulated when stimulated by cytokines, such as TNF‐α and IFN‐γ.^299^, ^300^ CXCL9, which is referred to as monokine induced by gamma IFN (MIG), primarily mediates lymphocyte infiltration into the lesion site and inhibits tumor growth.^301^ It has been reported that CXCL10, or IFN gamma‐inducible protein 10, is intensely induced by IFN‐α/β as well as IFN‐γ, but weakly induced by TNF‐α.^302^ CXCL11 is considered the predominant CXCR3 agonist due to its stronger potency than CXCL9 or CXCL10, is a major chemoattractant for effector T cells, and stimulates calcium flux and receptor desensitization.^303^ CXCR3 is predominantly expressed on CD4^+^ and CD8^+^ T lymphocytes. In the CD4^+^ subpopulation, CXCR3 is abundant on proinflammatory Th1 cells, but was also present on FOXp3+ Tregs.^304^ It is widely believed that the CXCL9, ‐10, ‐11/CXCR3 signaling axis modulates immune cell polarization and activation and directs immune cells toward their focal sites, which include macrophages, cytotoxic lymphocytes (CTL), and NK cells, among others.^300^, ^305^

#### 3.6.2 Targeting CXCL9, ‐10, ‐11/CXCR3 axis for cancer therapy

Recent studies have reported that CXCR3 expression levels in clinical tumor specimens correlate with metastatic potential and patient prognosis, making it feasible to use the CXCL9, ‐10, ‐11/CXCR3 axis as a predictor of treatment outcomes, although the relationship between expression of these three ligands and tumor recurrence or metastasis remains controversial.^31^, ^306^, ^307^ Using shRNA‐mediated silencing, Wightman et al.^308^ determined that CXCL10/CXCR3 coexpression increased tumor cell metastasis and recurrence in both in vitro and in vivo analyses of B16F1 melanoma. Simultaneous reduction of CXCR3, CXCL9, and/or CXCL10 expression suppresses cancer metastasis rates in melanoma,^309^ CRC,^310^ and breast cancer models.^311^ Data from a study on radiotherapy for head and neck cancer showed that circulating lymphocyte populations correlated with serum CXCL10 concentrations, but not with CXCL9.^312^ Mitsuhashi et al.^313^ noted that the pretreatment serum concentrations of CXCL10 and CXCL11 in lung cancer patients receiving anti‐PD‐1 antibodies were significantly correlated with clinical outcomes, and identified tumor‐derived CXCL10/11 as a potential circulating biomarker for monitoring drug treatment sensitivity. However, some studies have found a contrary relationship between the expression levels of CXCL9/CXCL10 and poor prognosis, drawing negative conclusion for clinical development.^314^, ^315^

Drugs that enhance the expression of paracrine CXCL9, ‐10, and ‐11 and inactivate CXCR3 expression on cancer cells have exhibited antitumor activity in several tumor models. It has been suggested that blocking PD‐1 checkpoint in mouse colon cancer cells (MC38) resulted in increased levels of IFN‐γ‐induced chemokines CXCL9 and CXCL10. In contrast, reducing CXCL9 and CXCL10 expression in animal models significantly decreased the efficacy of anti‐PD‐1 drugs and limited the aggregation of CD8^+^ T cells within the TME.^316^ In lung and renal cell carcinoma models, intratumoral injection of CXCL9 or CXCL10 proteins, respectively, reduced neovascularization and delayed tumor growth by inducing tumor‐infiltrating CXCR3^+^ monocytes.^317^, ^318^ Additionally, a novel CXCL10 fusion protein (IP10‐scFv) coadministered with CTLs successfully induced tumor‐infiltrating lymphocytes and prolonged survival in mice.^319^ CXCL11 is a controversial target for cancer treatment because it helps induce Tregs migration. In a mouse model of mesothelioma, selective lysing virus transfected with CXCL11 was reported to enhance CTL and NK cell infiltration into TME, but not CD4^+^ T cells.^320^ On the contrary, CXCL11 expression was remarkably upregulated in rectal adenocarcinoma, but was not correlated with a better prognosis in cancer patients.^321^ Pharmacological antagonism of AMG487 against CXCR3 was effective in inhibiting the proliferation of osteosarcoma and colon cancer cells in vitro and attenuated lung metastasis in mouse tumor models.^322^, ^323^

#### 3.6.3 Roles of the CXCL9, ‐10, ‐11/CXCR3 axis in neurological diseases

It has been shown that CXCR3 and its ligands are expressed at high levels in CSF and peripheral blood of patients with neurological disorders and are potentially useful regulators of neuroinflammatory response.^324^, ^325^ MS is a common chronic inflammatory disease of the CNS characterized by the damage of myelin and oligodendrocytes by inflammatory cells. Early in 1999, Balashov et al.^326^ reported that CXCL10 was expressed by astrocytes in MS brain lesions and that CXCR3^+^ T cells were found to be increased in the blood of relapsed/advanced MS patients. However, treatment of rats with EAE with monoclonal antibodies against CXCL10 resulted in an increase in infiltrating CD4^+^ Th1 cells in the CNS and an exacerbation of disease grading in animal models, suggesting that CXCL10 may play a specific inhibitory role in Th1‐mediated disease development.^327^ In another study, Chung and Liao^328^ revealed no significant differences in time to disease onset and severity between CXCR3‐deficient (CXCR3^–/–^) mice and wild‐type mice. However, pathological sections revealed more severe and extensive demyelination phenomena and axonal damage in CXCR3^–/–^ mice. Furthermore, in vitro studies indicated that astrocytes respond to infection by upregulating CXCL10 mRNA expression and releasing CXCL10 into the supernatant, which is completely abolished by CXCR3 antagonists.^329^ Gliomas account for the majority of CNS tumors, with glioblastoma progression involving glioma stem cells (GSCs) that are refractory to diverse therapeutic options. Shono et al.^330^ reported that CXCL10 and CXCR3 were increased in GSCs and a malignant glioma model, celecoxib inhibited the expression of CCL2 and CXCR3 in an NF‐κB‐dependent manner, and in addition, silencing of CCL2 led to a decrease in GSC viability. Sharma et al.^331^ used high‐throughput tissue microarrays to detect CXCR3 and CXCL10 immuno‐expression in glioblastoma multiforme (GBM) and diffuse astrocytoma (DA) tissues. Their results showed that among 129 analyzable samples, strong CXCR3 and CXCL10 expression was observed in 72.7 and 50.7% of GBM cases, respectively, whereas CXCR3 and CXCL10 expression in DA cases was 31.8 and 24.5%, respectively. Moreover, CXCR3 antagonist NBI‐74330 inhibited the growth of GL261 gliomas and increased the median survival time of CXCR3^–/–^ mice, whereas NBI‐74330 did not affect the infiltration of CXCR3+ NK and NKT cells within gliomas, suggesting that CXCR3 may not be a major pathway for NK and NKT cells to enter gliomas.^332^ Xia et al.^333^ used tissues from AD patients for immunohistochemistry to demonstrate extensive expression of CXCR3 in brain structures. In addition, they found that CXCL10‐positive astrocytes were also greatly increased in AD patients compared with controls. Recently, an experiment investigating CXCR3 antagonists in the amyloid precursor protein (APP)/presenilin 1 (PS1) transgenic mouse model of AD showed that CXCR3 antagonists increased Aβ phagocytosis in microglia and ameliorated behavioral deficits in diseased mice, suggesting that CXCR3 axis mediates AD‐like pathology in APP/PS1 mice and could be a therapeutic candidate for AD.^334^

Taken together, the aforementioned studies indicate that targeting the CXCL9, ‐10, ‐11/CXCR3 axis may have potential for treatment of both cancers and neurodegeneration.

### 3.7 The CXCL12–CXCR4/CXCR7 signaling axis

#### 3.7.1 Physiological role of the CXCL12–CXCR4/CXCR7 axis

When chemokines bind to their cognate receptors on target cells, they will perform a range of important functions within the tissues. CXCL12, also known as stromal cell‐derived factor‐1, is the only ligand for CXCR4, which is predominantly secreted by stromal fibroblasts, osteoblasts, and vascular endothelial cells. CXCL12 has been highly conserved during evolution and is one of the most primitive chemokines.^335^ By interacting with CXCR4 and ACKR3, CXCL12 is essential for the development of the brain, cardiovascular system, hematopoietic organs, and reproductive cells, and thus the chemokine network of CXCL12/CXCR4/ACKR3 signaling is necessary for life.^336^ Studies have shown that CXCL12 secretion is also involved in a range of pathological processes, such as cell damage, heart failure, and inflammation during chemotherapy or after organ irradiation.^336^ Increased CXCL12 expression has also been observed in the hypoxic and proangiogenic environment within tumors or during autoimmune diseases.^337^ Functioning as a GPCRs, CXCR4 is primarily expressed on the surface of endothelial mature cells, precursor cells, and pericytes.^338^ Many factors are implicated in the regulation of CXCR4 expression, including hypoxia, stress, and injury, with HIF‐1α being the most important regulator.^338^ Also, CXCR4 expression is influenced by IL‐17A released from T cells^339^ as well as IL‐5, IFN‐γ, and TGF‐β secreted by stromal cells.^340^ Although CXCR7 is a member of the GPCR family, it does not induce cellular signaling mediated by G protein subunits (α, β, and γ), its main role is to establish and maintain the gradient of its ligands CXCL11 and CXCL12 on both sides of the cell membrane.^341^ The process for activating CXCR7 is as follows: CXCR7 binds to the ligand and the CXCR7–ligand complex is subsequently internalized by the cell membrane, a process that leads to the degradation of the ligand while the receptor moves back to the cell membrane.

The CXCL12/CXCR4 signaling axis exerts a regulatory effect on the secretion of cytokines and chemokines, as it is shown to induce the expression of TNF‐α, IL‐1α/β, CXCL5, and some other chemokines, but it does not affect the expression of IL‐2 or IFN‐γ.^336^ The CXCL12/CXCR4 axis also modulates vascular endothelial adhesion and actin polymerization responses, as well as accommodates the migration of bone MSCs (BMSCs) underneath and beneath leukemic cells.^342^, ^343^ The two receptors CXCR4 and CXCR7 can interact to form a heterodimer, which results in enhanced CXCL12‐induced signaling via G proteins. Compared with cells transfected with CXCR4 only, coexpression of CXCR4 and CXCR7 on HEK293 cells resulted in higher calcium flux and more β‐arrestin recruitment through activation of downstream ERK signal cascade.^344^, ^345^ The interactions between CXCL12, CXCR4, and CXCR7 display considerable complexity under physiological conditions. When this interaction is disrupted, regulation of this axis can influence the progression of diseases including cancer, CNS, cardiac, and autoimmune diseases.^336^, ^346^

#### 3.7.2 The CXCL12–CXCR4/CXCR7 contributes to cancer progression

Numerous studies have explored the role of the CXCL12–CXCR4/CXCR7 axis in various cancer types in recent years.^346^, ^347^, ^348^ Under the regulation of the CXCL12–CXCR4/CXCR7 signaling axis, cancer tissues can exhibit enhanced cell migration and proliferation by activating signaling cascades within tumor cells, as well as regulate angiogenesis and induce metastasis through the vascular endothelial growth factor (VEGF). Thus, drugs targeting CXCR4 and/or CXCR7 can influence cancer progression pathways by regulating the CXCR4/CXCR7–CXCL12 axis. Several studies have reported that CXCL12 can stimulate the proliferation of various tumor cell lines, including melanoma,^349^ glioma,^350^ small cell lung cancer,^351^ gastric cancer,^352^ pancreatic cancer,^353^ and CRC.^354^ Moreover, CXCL12/CXCR4 regulates EMT in sacral chondrosarcoma,^355^ oral squamous cell carcinoma,^356^ and glioblastoma.^357^ Furthermore, the CXCL12/CXCR4 axis is also critical for tumor cell metastasis and drug resistance in cancer therapy.^358^, ^359^, ^360^ Inhibition of the PI3K/AKT/NF‐κB signaling pathway by downregulating CXCR4 significantly reduced cell proliferation and increased apoptosis in osteosarcoma cells.^361^ Notch positively controls CXCL12/CXCR4 function in myeloma cell lines, and in vivo blockade of Notch markedly limits myeloma cell infiltration into bone marrow of mouse xenografts.^362^ Interference with CXCR4 expression using CXCR4 antagonist or lentivirus shRNA can effectively inhibit tumor cell proliferation and invasion in breast cancer,^363^ human hilar cholangiocarcinoma,^364^ laryngeal squamous carcinoma,^365^ and esophageal carcinoma.^366^ Long et al.^367^ investigated the ability to reduce the proliferation of HEC‐1‐A cells after inhibiting higher mRNA and protein expression levels of CXCR4 and CXCR7 in endometrial adenocarcinoma by RNA interference. Apart from these findings, immunohistochemistry showed that elevated expression of CXCR7 was related to increased tumor grade of prostate cancer, and its overexpression also increased the release of VEGF and the proinflammatory cytokine IL‐8, which might promote the invasiveness of tumor cells.^368^, ^369^ Importantly, enhanced CXCR7 expression was associated with poor prognosis in patients with prostate cancer and glioblastoma, suggesting that CXCR7 may serve as a prognostic biomarker for these two tumors.^370^, ^371^ In an animal model of prostate cancer, coadministration of CCX771 (a CXCR7 inhibitor) and enzalutamide remarkably inhibited tumor growth and macrovascular formation, thus suppressing the drug resistance of enzalutamide.^372^

#### 3.7.3 Other diseases involved in CXCL12–CXCR4/CXCR7 regulation

CXCL12 is involved in the release of inflammatory factors during the immune process and may influence the pathogenesis of atherosclerosis and osteoarthritis.^373^, ^374^ CXCL12 from the subchondral layer binds to CXCR4 in chondrocytes and induces articular cartilage degeneration by promoting a shift of TGF‐β receptor type I (TβRI) from activin receptor‐like kinase 5 (ALK5) to ALK1 in chondrocytes.^373^ Gao et al.^375^ found that CXCL12 interacted with CXCR4 to activate the GSK‐3β/β‐catenin/TCF21 pathway, thereby reducing plasma HDL‐C levels and the efficacy of reverse cholesterol transport, inhibiting ABCA1‐dependent cholesterol efflux from macrophages, and exacerbating atherosclerosis. During embryonic development, CXCR7 expression is critical for cardiovascular system function. In vitro studies have shown that CXCL12 induced migration of oligodendrocyte precursor cells and angiogenesis of HUVECs through CXCR4‐activated MEK/ERK and PI3K/AKT pathways. Knockdown of CXCR4 could also reverse these phenomena and downregulated the MEK/ERK and PI3K/AKT pathways.^376^, ^377^ Furthermore, CXCR7 also facilitates cardiac remodeling by activating endothelial cell proliferation and angiogenesis.^378^ The gradient of CXCL12 directs CXCR4‐positive neural stem cells to differentiate into neuronal cells, including oligodendrocytes, astrocytes, and so on, toward the damaged tissue.^379^ Similarly, the redistribution of CXCL12 due to the increased expression of CXCR7 at the marginal sites of endothelial cells explains the pathogenesis of MS.^380^ Several modulators that have entered clinical trials targeting the CXCL12–CXCR4/CXCR7 signaling axis are summarized in Table 2.

### 3.8 The CXCL13/CXCR5 axis

#### 3.8.1 A brief introduction of the CXCL13/CXCR5 axis

Originally known as B cell‐inducible chemokine 1 or B lymphocyte chelator, CXCL13 forms a unisexual ligand–receptor pair with CXCR5 that is essential for the homeostatic organization of the B cell compartment of secondary lymphoid tissue. CXCL13 is secreted constitutively by stromal cells (e.g., follicular high endothelial vein cells) in the B‐cell region of secondary lymphoid tissues (follicles), thus correctly orchestrating CXCR5^+^ T/B lymphocytes and macrophages from the blood into the follicles.^381^, ^382^, ^383^ CXCR5 is also named Burkitt's lymphoma receptor 1 because it was initially isolated from Burkitt's lymphoma and its expression is detected on tonsillar B cells as well as in all peripheral blood. Similar to other chemokine receptors, CXCR5 is kinetically modulated on T cells and is upregulated on memory/effector T cells following T cell receptor stimulation, whereas IL‐2 causes its downregulation.^384^, ^385^ CXCR5 shares 40% amino acid homology with CXCR1, so when CXCL13 activates CXCR5, this signaling axis can lead to intracellular calcium ion influx and induce the activation of several intracellular signaling cascades, such as PI3K/AKT, MAPK/ERK, and Rac pathways, playing a role in immune disorders as well as tumor progression.^384^, ^386^

#### 3.8.2 Roles of the CXCL13/CXCR5 axis in cancer progression

Abnormal activation of CXCL13/CXCR5 signaling has been implicated in the development of several advanced solid cancers as well as hematological malignancies. For instance, expression of CXCL13 and/or CXCR5 correlates significantly with tumorigenesis, and CXCL13 is considered a predictive factor for lung cancer progression and early diagnosis.^387^, ^388^ A recent interesting study focused on the correlation between CXCL13 and patient prognosis in patients with squamous lung cancer receiving corticosteroids and chemotherapy.^389^ This study determined that perivascular CXCL13‐positive niches induced the forming of tertiary lymphoid structures, which was correlated with better patient survival. However, studies also have shown that treatment with steroids compromised the formation of these tertiary lymphoid structures, compared with those who were untreated.^389^ There is also evidence that CXCL13 secreted by PD‐1 high expressing tumor infiltrating CD8+ lymphocytes helps to induce other immune cell subsets into TME, including B lymphocytes and T follicular helper cells (TFH cells). Moreover, CXCL13 and CXCL13+ immune cells in the TME of nonsmall cell lung cancer strongly predicted patients’ response to anti‐PD‐1 therapy, correlating with improved durable response and prolonged OS.^390^ It seems that there is a strong correlation between the CXCL13/CXCR5 axis and breast cancer progression, which have been verified in several studies. Using microarray analysis, CXCL13 was found to be the most overexpressed chemokine in breast cancer tissues when normal breast tissues were used as controls, while a positive correlation was identified between the expression of CXCL13 and CXCR5.^391^ In addition, expression levels of CXCL13 and CXCR5 could be potential biomarkers for diagnosis and prognosis for breast cancer.^392^, ^393^, ^394^ Of interest, the results of a recent in vitro study have suggested that an anti‐CXCL13 antibody reduced the levels of activated ERK and cyclin D1 and potentiated the cleavage of caspase‐9, thereby reducing the viability of MDA‐MB‐231 breast cancer cells.^395^ Furthermore, treatment with the anti‐CXCL13 antibody inhibited ERK activation and slowed tumor growth in a 4T1 mouse model of breast cancer, thus providing a rationale for clinical trials targeting CXCL13.^396^ Immunohistochemical analysis of specimens from various tumors (including ovarian cancer,^397^ CRC,^398^ prostate cancer,^399^ melanoma,^400^ clear renal cell carcinoma,^401^ and nasopharyngeal carcinoma^402^) showed that CXCL13 and CXCR5 were markedly elevated in tumors, as compared with normal tissue, and contributed to the ability of tumor cells to proliferate, migrate, and invade, ultimately affecting tumor progression, metastasis, and OS. Elucidating CXCL13/CXCR5 signaling effects and downstream signaling pathways will help investigate the molecular mechanisms that control tumor progression and responses to targeted therapies, accelerating the translation of drug research into clinical precision medicine.

#### 3.8.3 The CXCL13/CXCR5 axis in autoimmune and infectious diseases

The formation of CXCL13/CXCR5‐derived tertiary lymphoid structures has been associated with the evolution of a divergent range of diseases, including MS, myasthenia gravis, Sjögren's disease, RA, bullous pemphigoid, Graves thyroiditis, and infectious diseases. We already know that abnormal lymphocyte aggregates develop within the affected synovial membrane in patients with RA, and in fact, intense expression of CXCL13 mRNA and protein was detected in areas of B‐lymphocyte aggregation, thus facilitating endothelial progenitor cell homing and angiogenesis during RA progression.^403^ A similar role of CXCL13 and/or CXCR5 in regulating the formation of ectopic lymphoid structures was subsequently found in myasthenia gravis,^404^ Sjögren's syndrome,^405^ and SLE.^406^ Further, the expression levels of CXCL13 are associated with the progression and unfavorable prognosis of the above‐mentioned diseases and has been suggested as a biomarker to predict the progression of these diseases. Indeed, the CXCL13/CXCR5 axis not only affects the abnormal activity and differentiation of B cells, but also involves the drive of TFH cells. For example, CXCR5+/CD4+ T cells in circulation were similar to TFH cells and have been noted in SLE patients, and they are also engaged in promoting the differentiation of pathological B cells and are associated with disease progression.^407^

Before the cloning of CXCL13, CXCR5 was determined to be as a coreceptor required during HIV‐2 infection of host cells, rendering TFH cells vulnerable to viral infection. During HIV infection, the expression of CXCL13 and CXCR5 was demonstrated to be dysregulated, for example, as HIV infection progressed, the number of CXCR5+ B lymphocytes decreased, whereas plasma levels of CXCL13 increased.^408^ Subsequent studies confirmed the elevation of serum CXCL13 levels during chronic HIV infection and demonstrated an association of CXCL13 secretion with both viral load and disease progression.^408^, ^409^, ^410^ Li et al.^411^ revealed that in patients with chronic hepatitis B (CHB), CXCR5^+^CD8^+^ T cells were partially depleted but possessed greater antiviral capacity than the CXCR5^−^ subpopulation; furthermore, the CXCL13 from CHB patients promoted the infiltration of intrahepatic CXCR5^+^CD8^+^ T cells, a subpopulation that produces anti‐HBV‐specific IFN‐γ and IL‐21 and improves treatment response in CHB patients. Notably, administration of CXCR5^+^CD8^+^ T cells to CHB mice resulted in a significant reduction in HBsAg expression in the same study. Intriguingly, overexpression of CXCL13 was detected in the muscles of monkeys chronically infected with the Lyme disease pathogen *Borrelia burgdorferi*, but the bacterium did not appear to have an effect on plasma levels of CXCL13 chemokines. In contrast, once *B. burgdorferi* infected the CNS, constitutively elevated levels of CXCL13 were observed in the CSF, contributing to the formation of ectopic lymphoid tissue within the CNS.^412^ Neurosyphilis is often an advanced manifestation of a long‐term infection, usually presenting as stroke‐like symptoms or chronic meningitis. CXCL13 is also thought to be implicated in infection with *Treponema pallidum* (the causative agent of syphilis), with CXCL13 levels within the CSF of syphilis patients being 100‐fold higher than those in uninfected individuals. Pathologically, activation and enrichment of B cells and ectopic germinal centers were observed in the CNS of neurosyphilis patients, suggesting that *T. pallidum* infection leads to overexpression of CXCL13 in the CFS, causing a strong humoral response that promotes destruction of neural tissue.^413^

## 4 CONCLUSIONS

**FIGURE 4:**
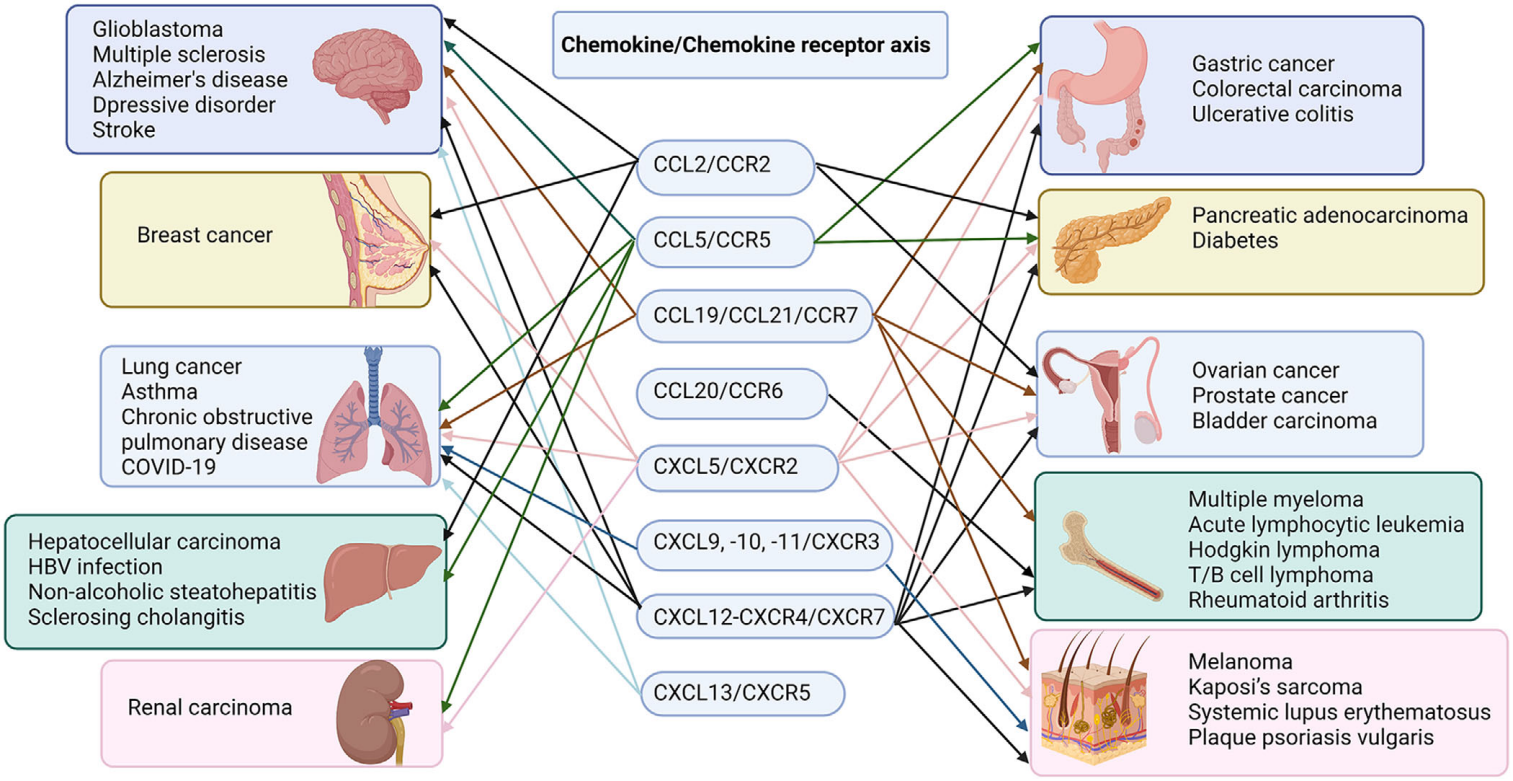
Chemokines/chemokine receptor axis in cancers and inflammatory diseases. Multiple chemokines/chemokine receptor axes play important roles in different tumor types and inflammation‐related diseases. Almost all organs of the body are regulated by the chemokines/chemokine receptor axes that predominantly affect the progression of tumors and the immune response during inflammation. Development of drugs targeting chemokines or their receptors is a potential strategy for the treatment of these diseases (the figure was created using biorender.com)

The chemokine system is an extraordinarily complex defense entity in the body, consisting of a huge array of interplaying ligands, receptors, and regulatory molecules that are involved in various cellular processes. Among them, chemotaxis of immune cells (especially lymphocytes) is its core biological function, but its impact goes far beyond that. The contribution of the chemokine network in physiopathological processes is enormous, involving organ development, immune surveillance, inflammation, infection, as well as innate and adaptive immune responses. It has conclusively been shown that the chemokine/chemokine receptor axis has a tumorigenic role in many different cancer models and clinics (Figure 4) and is also involved in immunosuppression and protective TME formation and can serve as prognostic bioindicators for many hematologic tumors as well as solid tumors. Modulation of the expression of chemokines or their homologous receptors on tumor cells or immune cells in TME provides a basis for the exploitation of new drugs for clinical evaluation in cancer immunotherapy. In fact, in addition to its vital role in tumors, almost all inflammatory diseases involve chemokines and their receptors in one way or another. Nevertheless, many unknown aspects of the role of chemokines and chemokine receptors in human disease remain to be unfolded, which necessitates strong efforts in much more basic animal studies as well as clinical researches.

## CONFLICT OF INTEREST

The authors declare no conflict of interest.

## AUTHOR CONTRIBUTIONS

All authors read and approved the final manuscript. H. L. wrote the initial manuscript and created the tables and figures. M. W. and X. Z. revised the manuscript and approved the final version.

## ETHICS STATEMENT

Not applicable.

## Data Availability

The data included in this article are available upon request from the corresponding authors.

## References

[1] Griffith JW , Sokol CL , Luster AD . Chemokines and chemokine receptors: positioning cells for host defense and immunity. Annu Rev Immunol. 2014;32:659‐702. 24655300 10.1146/annurev-immunol-032713-120145

[2] Zlotnik A , Yoshie O . The chemokine superfamily revisited. Immunity. 2012;36(5):705‐716. 22633458 10.1016/j.immuni.2012.05.008 PMC3396424

[3] Bonecchi R , Graham GJ . Atypical chemokine receptors and their roles in the resolution of the inflammatory response. Front Immunol. 2016;7:224. 27375622 10.3389/fimmu.2016.00224 PMC4901034

[4] McCully ML , Kouzeli A , Moser B . Peripheral tissue chemokines: homeostatic control of immune surveillance T cells. Trends Immunol. 2018;39(9):734‐747. 30001872 10.1016/j.it.2018.06.003

[5] Anders HJ , Romagnani P , Mantovani A . Pathomechanisms: homeostatic chemokines in health, tissue regeneration, and progressive diseases. Trends Mol Med. 2014;20(3):154‐165. 24440002 10.1016/j.molmed.2013.12.002

[6] Mollica Poeta V , Massara M , Capucetti A , Bonecchi R . Chemokines and chemokine receptors: new targets for cancer immunotherapy. Front Immunol. 2019;10:379. 30894861 10.3389/fimmu.2019.00379 PMC6414456

[7] Legler DF , Thelen M . Chemokines: chemistry, biochemistry and biological function. Chimia (Aarau). 2016;70(12):856‐859. 28661356 10.2533/chimia.2016.856

[8] Hughes CE , Nibbs RJB . A guide to chemokines and their receptors. FEBS J. 2018;285(16):2944‐2971. 29637711 10.1111/febs.14466 PMC6120486

[9] Balkwill F . Cancer and the chemokine network. Nat Rev Cancer. 2004;4(7):540‐550. 15229479 10.1038/nrc1388

[10] Chow MT , Luster AD . Chemokines in cancer. Cancer Immunol Res. 2014;2(12):1125‐1131. 25480554 10.1158/2326-6066.CIR-14-0160 PMC4258879

[11] Nagarsheth N , Wicha MS , Zou W . Chemokines in the cancer microenvironment and their relevance in cancer immunotherapy. Nat Rev Immunol. 2017;17(9):559‐572. 28555670 10.1038/nri.2017.49 PMC5731833

[12] Mukaida N , Sasaki S , Baba T . Chemokines in cancer development and progression and their potential as targeting molecules for cancer treatment. Mediators Inflamm. 2014;2014:170381. 24966464 10.1155/2014/170381 PMC4055660

[13] Ozga AJ , Chow MT , Luster AD . Chemokines and the immune response to cancer. Immunity. 2021;54(5):859‐874. 33838745 10.1016/j.immuni.2021.01.012 PMC8434759

[14] Liu H , Yang Z , Lu W , et al. Chemokines and chemokine receptors: a new strategy for breast cancer therapy. Cancer Med. 2020;9(11):3786‐3799. 32253815 10.1002/cam4.3014 PMC7286460

[15] Singh N , Baby D , Rajguru JP , Patil PB , Thakkannavar SS , Pujari VB . Inflammation and cancer. Ann Afr Med. 2019;18(3):121‐126. 31417011 10.4103/aam.aam_56_18 PMC6704802

[16] Chen L , Deng H , Cui H , et al. Inflammatory responses and inflammation‐associated diseases in organs. Oncotarget. 2018;9(6):7204‐7218. 29467962 10.18632/oncotarget.23208 PMC5805548

[17] Sokol CL , Luster AD . The chemokine system in innate immunity. Cold Spring Harb Perspect Biol. 2015;7(5):a016303. 25635046 10.1101/cshperspect.a016303 PMC4448619

[18] Burg JS , Ingram JR , Venkatakrishnan AJ , et al. Structural biology. Structural basis for chemokine recognition and activation of a viral G protein‐coupled receptor. Science. 2015;347(6226):1113‐1117. 25745166 10.1126/science.aaa5026 PMC4445376

[19] Fox JC , Thomas MA , Dishman AF , et al. Structure‐function guided modeling of chemokine‐GPCR specificity for the chemokine XCL1 and its receptor XCR1. Sci Signal. 2019;12(597):eaat4128. 31481523 10.1126/scisignal.aat4128 PMC6733756

[20] Zhao S , Wu B , Stevens RC . Advancing chemokine GPCR structure based drug discovery. Structure. 2019;27(3):405‐408. 30840872 10.1016/j.str.2019.02.004

[21] Soulika AM , Pleasure DE . Chemokines. Encyclopedia of the Neurological Sciences. 2014:764‐769.

[22] Yung SC , Farber JM . Chemokines. Handbook of Biologically Active Peptides. 2013:656‐663.

[23] Wang L , Zhang YL , Lin QY , et al. CXCL1‐CXCR2 axis mediates angiotensin II‐induced cardiac hypertrophy and remodelling through regulation of monocyte infiltration. Eur Heart J. 2018;39(20):1818‐1831. 29514257 10.1093/eurheartj/ehy085

[24] Lepsenyi M , Algethami N , Al‐Haidari AA , et al. CXCL2‐CXCR2 axis mediates αV integrin‐dependent peritoneal metastasis of colon cancer cells. Clin Exp Metastasis. 2021;38(4):401‐410. 34115261 10.1007/s10585-021-10103-0 PMC8318971

[25] Sun X , He X , Zhang Y , et al. Inflammatory cell‐derived CXCL3 promotes pancreatic cancer metastasis through a novel myofibroblast‐hijacked cancer escape mechanism. Gut. 2022;71(1):129‐147. 33568427 10.1136/gutjnl-2020-322744

[26] Domschke G , Gleissner CA . CXCL4‐induced macrophages in human atherosclerosis. Cytokine. 2019;122:154141. 28899579 10.1016/j.cyto.2017.08.021

[27] Zhang W , Wang H , Sun M , et al. CXCL5/CXCR2 axis in tumor microenvironment as potential diagnostic biomarker and therapeutic target. Cancer Commun (Lond). 2020;40(2‐3):69‐80. 32237072 10.1002/cac2.12010 PMC7163794

[28] Wang X , Dai Y , Zhang X , et al. CXCL6 regulates cell permeability, proliferation, and apoptosis after ischemia‐reperfusion injury by modulating Sirt3 expression via AKT/FOXO3a activation. Cancer Biol Ther. 2021;22(1):30‐39. 33241954 10.1080/15384047.2020.1842705 PMC7834049

[29] Wang YH , Shen CY , Lin SC , et al. Monocytes secrete CXCL7 to promote breast cancer progression. Cell Death Dis. 2021;12(12):1090. 34789744 10.1038/s41419-021-04231-4 PMC8599470

[30] Han ZJ , Li YB , Yang LX , Cheng HJ , Liu X , Chen H . Roles of the CXCL8‐CXCR1/2 axis in the tumor microenvironment and immunotherapy. Molecules. 2021;27(1):137. 10.3390/molecules27010137 PMC8746913 35011369

[31] Neo SY , Lundqvist A . The multifaceted roles of CXCL9 within the tumor microenvironment. Adv Exp Med Biol. 2020;1231:45‐51. 32060845 10.1007/978-3-030-36667-4_5

[32] Karin N , Razon H . Chemokines beyond chemo‐attraction: cXCL10 and its significant role in cancer and autoimmunity. Cytokine. 2018;109:24‐28. 29449068 10.1016/j.cyto.2018.02.012

[33] Gao Q , Zhang Y . CXCL11 signaling in the tumor microenvironment. Adv Exp Med Biol. 2021;1302:41‐50. 34286440 10.1007/978-3-030-62658-7_4

[34] Janssens R , Struyf S , Proost P . The unique structural and functional features of CXCL12. Cell Mol Immunol. 2018;15(4):299‐311. 29082918 10.1038/cmi.2017.107 PMC6052832

[35] Gao SH , Liu SZ , Wang GZ , Zhou GB . CXCL13 in cancer and other diseases: biological functions, clinical significance, and therapeutic opportunities. Life (Basel). 2021;11(12):1282. 10.3390/life11121282 PMC8708574 34947813

[36] Cereijo R , Gavaldà‐Navarro A , Cairó M , et al. CXCL14, a brown adipokine that mediates brown‐fat‐to‐macrophage communication in thermogenic adaptation. Cell Metab. 2018;28(5):750‐763.e6. 30122557 10.1016/j.cmet.2018.07.015

[37] Broxmeyer HE , Cooper SH , Ropa J . CXCL15/Lungkine has suppressive activity on proliferation and expansion of multi‐potential, erythroid, granulocyte and macrophage progenitors in S‐phase specific manner. Blood Cells Mol Dis. 2021;91:102594. 34520986 10.1016/j.bcmd.2021.102594 PMC9231597

[38] Korbecki J , Bajdak‐Rusinek K , Kupnicka P , et al. The role of CXCL16 in the pathogenesis of cancer and other diseases. Int J Mol Sci. 2021;22(7):3490. 33800554 10.3390/ijms22073490 PMC8036711

[39] Xiao S , Xie W , Zhou L . Mucosal chemokine CXCL17: what is known and not known. Scand J Immunol. 2021;93(2):e12965. 32869346 10.1111/sji.12965

[40] Liu SS , Liu C , Lv XX , et al. The chemokine CCL1 triggers an AMFR‐SPRY1 pathway that promotes differentiation of lung fibroblasts into myofibroblasts and drives pulmonary fibrosis. Immunity. 2021;54(9):2042‐2056.e8. 34407391 10.1016/j.immuni.2021.06.008

[41] Lv LL , Feng Y , Wen Y , et al. Exosomal CCL2 from tubular epithelial cells is critical for albumin‐induced tubulointerstitial inflammation. J Am Soc Nephrol. 2018;29(3):919‐935. 29295871 10.1681/ASN.2017050523 PMC5827595

[42] Zhao X , Gu M , Xu X , et al. CCL3/CCR1 mediates CD14(+)CD16(−) circulating monocyte recruitment in knee osteoarthritis progression. Osteoarthr Cartil. 2020;28(5):613‐625. 10.1016/j.joca.2020.01.009 32006659

[43] Mukaida N , Sasaki SI , Baba T . CCL4 signaling in the tumor microenvironment. Adv Exp Med Biol. 2020;1231:23‐32. 32060843 10.1007/978-3-030-36667-4_3

[44] Kranjc MK , Novak M , Pestell RG , Lah TT . Cytokine CCL5 and receptor CCR5 axis in glioblastoma multiforme. Radiol Oncol. 2019;53(4):397‐406. 31747383 10.2478/raon-2019-0057 PMC6884928

[45] Li F , Du X , Lan F , et al. Eosinophilic inflammation promotes CCL6‐dependent metastatic tumor growth. Sci Adv. 2021;7(22):eabb5943. 34039594 10.1126/sciadv.abb5943 PMC8153717

[46] Xie C , Ye F , Zhang N , Huang Y , Pan Y , Xie X . CCL7 contributes to angiotensin II‐induced abdominal aortic aneurysm by promoting macrophage infiltration and pro‐inflammatory phenotype. J Cell Mol Med. 2021;25(15):7280‐7293. 34189838 10.1111/jcmm.16757 PMC8335673

[47] Farmaki E , Kaza V , Chatzistamou I , Kiaris H . CCL8 promotes postpartum breast cancer by recruiting M2 macrophages. iScience. 2020;23(6):101217. 32535027 10.1016/j.isci.2020.101217 PMC7300153

[48] Li B , Zhang S , Huang N , et al. CCL9/CCR1 induces myeloid‑derived suppressor cell recruitment to the spleen in a murine H22 orthotopic hepatoma model. Oncol Rep. 2019;41(1):608‐618. 30365155 10.3892/or.2018.6809

[49] Rump L , Mattey DL , Kehoe O , Middleton J . An initial investigation into endothelial CC chemokine expression in the human rheumatoid synovium. Cytokine. 2017;97:133‐140. 28648867 10.1016/j.cyto.2017.05.023 PMC5516773

[50] Polosukhina D , Singh K , Asim M , et al. CCL11 exacerbates colitis and inflammation‐associated colon tumorigenesis. Oncogene. 2021;40(47):6540‐6546. 34625710 10.1038/s41388-021-02046-3 PMC8629429

[51] Marazioti A , Kairi CA , Spella M , et al. Beneficial impact of CCL2 and CCL12 neutralization on experimental malignant pleural effusion. PLoS One. 2013;8(8):e71207. 23967166 10.1371/journal.pone.0071207 PMC3743892

[52] Abu El‐Asrar AM , Berghmans N , Al‐Obeidan SA , et al. The CC chemokines CCL8, CCL13 and CCL20 are local inflammatory biomarkers of HLA‐B27‐associated uveitis. Acta Ophthalmol. 2019;97(1):e122‐e128. 30242977 10.1111/aos.13835

[53] Gu Y , Li X , Bi Y , et al. CCL14 is a prognostic biomarker and correlates with immune infiltrates in hepatocellular carcinoma. Aging (Albany NY). 2020;12(1):784‐807. 31927532 10.18632/aging.102656 PMC6977663

[54] Liu LZ , Zhang Z , Zheng BH , et al. CCL15 recruits suppressive monocytes to facilitate immune escape and disease progression in hepatocellular carcinoma. Hepatology. 2019;69(1):143‐159. 30070719 10.1002/hep.30134

[55] Shen W , Zhang X , Tang J , et al. CCL16 maintains stem cell‐like properties in breast cancer by activating CCR2/GSK3β/β‐catenin/OCT4 axis. Theranostics. 2021;11(5):2297‐2317. 33500726 10.7150/thno.51000 PMC7797668

[56] Chen YT , Hsu H , Lin CC , et al. Inflammatory macrophages switch to CCL17‐expressing phenotype and promote peritoneal fibrosis. J Pathol. 2020;250(1):55‐66. 31579932 10.1002/path.5350

[57] Cardoso AP , Pinto ML , Castro F , et al. The immunosuppressive and pro‐tumor functions of CCL18 at the tumor microenvironment. Cytokine Growth Factor Rev. 2021;60:107‐119. 33863622 10.1016/j.cytogfr.2021.03.005

[58] Yan Y , Chen R , Wang X , et al. CCL19 and CCR7 expression, signaling pathways, and adjuvant functions in viral infection and prevention. Front Cell Dev Biol. 2019;7:212. 31632965 10.3389/fcell.2019.00212 PMC6781769

[59] Kadomoto S , Izumi K , Mizokami A . The CCL20‐CCR6 axis in cancer progression. Int J Mol Sci. 2020;21(15):5186. 10.3390/ijms21155186 PMC7432448 32707869

[60] Sharma S , Kadam P , Dubinett S . CCL21 programs immune activity in tumor microenvironment. Adv Exp Med Biol. 2020;1231:67‐78. 32060847 10.1007/978-3-030-36667-4_7

[61] Ren G , Al‐Jezani N , Railton P , Powell JN , Krawetz RJ . CCL22 induces pro‐inflammatory changes in fibroblast‐like synoviocytes. iScience. 2021;24(1):101943. 33490888 10.1016/j.isci.2020.101943 PMC7809191

[62] Faura J , Bustamante A , Penalba A , et al. CCL23: a chemokine associated with progression from mild cognitive impairment to Alzheimer's disease. J Alzheimers Dis. 2020;73(4):1585‐1595. 31958084 10.3233/JAD-190753 PMC8010612

[63] Wang Y , Wu X , Geng M , et al. CCL24 protects renal function by controlling inflammation in podocytes. Dis Markers. 2021;2021:8837825. 34221188 10.1155/2021/8837825 PMC8221868

[64] Wu X , Sun M , Yang Z , et al. The roles of CCR9/CCL25 in inflammation and inflammation‐associated diseases. Front Cell Dev Biol. 2021;9:686548. 34490243 10.3389/fcell.2021.686548 PMC8416662

[65] Chen X , An Y , Zhang Y , et al. CCL26 is upregulated by nab‐paclitaxel in pancreatic cancer‐associated fibroblasts and promotes PDAC invasiveness through activation of the PI3K/AKT/mTOR pathway. Acta Biochim Biophys Sin (Shanghai). 2021;53(5):612‐619. 33764366 10.1093/abbs/gmab032

[66] Martínez‐Rodríguez M , Monteagudo C . CCL27 signaling in the tumor microenvironment. Adv Exp Med Biol. 2021;1302:113‐132. 34286445 10.1007/978-3-030-62658-7_9

[67] Mohan T , Deng L , Wang BZ . CCL28 chemokine: an anchoring point bridging innate and adaptive immunity. Int Immunopharmacol. 2017;51:165‐170. 28843907 10.1016/j.intimp.2017.08.012 PMC5755716

[68] Bergamaschi C , Pandit H , Nagy BA , et al. Heterodimeric IL‐15 delays tumor growth and promotes intratumoral CTL and dendritic cell accumulation by a cytokine network involving XCL1, IFN‐γ, CXCL9 and CXCL10. J Immunother Cancer. 2020;8(1):e000599. 32461349 10.1136/jitc-2020-000599 PMC7254133

[69] Fox JC , Nakayama T , Tyler RC , Sander TL , Yoshie O , Volkman BF . Structural and agonist properties of XCL2, the other member of the C‐chemokine subfamily. Cytokine. 2015;71(2):302‐311. 25497737 10.1016/j.cyto.2014.11.010 PMC4297508

[70] Pawelec P , Ziemka‐Nalecz M , Sypecka J , Zalewska T . The impact of the CX3CL1/CX3CR1 axis in neurological disorders. Cells. 2020;9(10):2277. 10.3390/cells9102277 PMC7600611 33065974

[71] Stewart TJ , Smyth MJ . Chemokine‐chemokine receptors in cancer immunotherapy. Immunotherapy. 2009;1(1):109‐127. 20635978 10.2217/1750743X.1.1.109

[72] Fernandez EJ , Lolis E . Structure, function, and inhibition of chemokines. Annu Rev Pharmacol Toxicol. 2002;42:469‐499. 11807180 10.1146/annurev.pharmtox.42.091901.115838

[73] Gerber PA , Hippe A , Buhren BA , Müller A , Homey B . Chemokines in tumor‐associated angiogenesis. Biol Chem. 2009;390(12):1213‐1223. 19804363 10.1515/BC.2009.144

[74] Fillion I , Ouellet N , Simard M , Bergeron Y , Sato S , Bergeron MG . Role of chemokines and formyl peptides in pneumococcal pneumonia‐induced monocyte/macrophage recruitment. J Immunol. 2001;166(12):7353‐7361. 11390486 10.4049/jimmunol.166.12.7353

[75] Fantuzzi L , Belardelli F , Gessani S . Monocyte/macrophage‐derived CC chemokines and their modulation by HIV‐1 and cytokines: a complex network of interactions influencing viral replication and AIDS pathogenesis. J Leukoc Biol. 2003;74(5):719‐725. 12960239 10.1189/jlb.0403175

[76] Montero J , Coll J , Sevilla N , Cuesta A , Bols NC , Tafalla C . Interleukin 8 and CK‐6 chemokines specifically attract rainbow trout (Oncorhynchus mykiss) RTS11 monocyte‐macrophage cells and have variable effects on their immune functions. Dev Comp Immunol. 2008;32(11):1374‐1384. 18572244 10.1016/j.dci.2008.05.004

[77] Li M , Chen L , Gao Y , et al. Recent advances targeting C‐C chemokine receptor type 2 for liver diseases in monocyte/macrophage. Liver Int. 2020;40(12):2928‐2936. 33025657 10.1111/liv.14687

[78] Bier A , Khashab R , Sharabi Y , Grossman E , Leibowitz A . Melatonin prevents T lymphocyte infiltration to the kidneys of hypertensive rats, induced by a high‐salt diet, by preventing the expression of CXCR3 ligand chemokines. Nutrients. 2021;13(10):3577. 34684578 10.3390/nu13103577 PMC8538338

[79] De Filippo K , Dudeck A , Hasenberg M , et al. Mast cell and macrophage chemokines CXCL1/CXCL2 control the early stage of neutrophil recruitment during tissue inflammation. Blood. 2013;121(24):4930‐4937. 23645836 10.1182/blood-2013-02-486217

[80] Mukai K , Tsai M , Saito H , Galli SJ . Mast cells as sources of cytokines, chemokines, and growth factors. Immunol Rev. 2018;282(1):121‐150. 29431212 10.1111/imr.12634 PMC5813811

[81] Menzies‐Gow A , Robinson DS . Eosinophil chemokines and chemokine receptors: their role in eosinophil accumulation and activation in asthma and potential as therapeutic targets. J Asthma. 2001;38(8):605‐613. 11758889 10.1081/jas-100107538

[82] Oliveira SH , Lukacs NW . The role of chemokines and chemokine receptors in eosinophil activation during inflammatory allergic reactions. Braz J Med Biol Res. 2003;36(11):1455‐1463. 14576899 10.1590/s0100-879x2003001100002

[83] Shinagawa K , Trifilieff A , Anderson GP . Involvement of CCR3‐reactive chemokines in eosinophil survival. Int Arch Allergy Immunol. 2003;130(2):150‐157. 12673069 10.1159/000069005

[84] Tecchio C , Cassatella MA . Neutrophil‐derived chemokines on the road to immunity. Semin Immunol. 2016;28(2):119‐128. 27151246 10.1016/j.smim.2016.04.003 PMC7129466

[85] Rajarathnam K , Schnoor M , Richardson RM , Rajagopal S . How do chemokines navigate neutrophils to the target site: dissecting the structural mechanisms and signaling pathways. Cell Signal. 2019;54:69‐80. 30465827 10.1016/j.cellsig.2018.11.004 PMC6664297

[86] Capucetti A , Albano F , Bonecchi R . Multiple roles for chemokines in neutrophil biology. Front Immunol. 2020;11:1259. 32733442 10.3389/fimmu.2020.01259 PMC7363767

[87] Liu K , Wu L , Yuan S , et al. Structural basis of CXC chemokine receptor 2 activation and signalling. Nature. 2020;585(7823):135‐140. 32610344 10.1038/s41586-020-2492-5

[88] Matsushima K , Larsen CG , DuBois GC , Oppenheim JJ . Purification and characterization of a novel monocyte chemotactic and activating factor produced by a human myelomonocytic cell line. J Exp Med. 1989;169(4):1485‐1490. 2926331 10.1084/jem.169.4.1485 PMC2189236

[89] Yoshimura T , Robinson EA , Tanaka S , Appella E , Leonard EJ . Purification and amino acid analysis of two human monocyte chemoattractants produced by phytohemagglutinin‐stimulated human blood mononuclear leukocytes. J Immunol. 1989;142(6):1956‐1962. 2921521

[90] Hao Q , Vadgama JV , Wang P . CCL2/CCR2 signaling in cancer pathogenesis. Cell Commun Signal. 2020;18(1):82. 32471499 10.1186/s12964-020-00589-8 PMC7257158

[91] Kadomoto S , Izumi K , Mizokami A . Roles of CCL2‐CCR2 axis in the tumor microenvironment. Int J Mol Sci. 2021;22(16):8530. 34445235 10.3390/ijms22168530 PMC8395188

[92] O'Connor T , Heikenwalder M . CCL2 in the tumor microenvironment. Adv Exp Med Biol. 2021;1302:1‐14. 34286437 10.1007/978-3-030-62658-7_1

[93] Charo IF , Myers SJ , Herman A , Franci C , Connolly AJ , Coughlin SR . Molecular cloning and functional expression of two monocyte chemoattractant protein 1 receptors reveals alternative splicing of the carboxyl‐terminal tails. Proc Natl Acad Sci USA. 1994;91(7):2752‐2756. 8146186 10.1073/pnas.91.7.2752 PMC43448

[94] Lou Q , Liu YL , Zhang SM , Li YY , Huang XF . CCL2 promotes angiogenesis of primary rat cardiac microvascular endothelial cells. Sheng Li Xue Bao. 2020;72(4):441‐448. 32820306

[95] Bosteels C , Fierens K , De Prijck S , et al. CCR2‐ and flt3‐dependent inflammatory conventional type 2 dendritic cells are necessary for the induction of adaptive immunity by the human vaccine adjuvant system AS01. Front Immunol. 2020;11:606805. 33519816 10.3389/fimmu.2020.606805 PMC7841299

[96] Lim SY , Yuzhalin AE , Gordon‐Weeks AN , Muschel RJ . Targeting the CCL2‐CCR2 signaling axis in cancer metastasis. Oncotarget. 2016;7(19):28697‐28710. 26885690 10.18632/oncotarget.7376 PMC5053756

[97] Tian DS , Peng J , Murugan M , et al. Chemokine CCL2‐CCR2 signaling induces neuronal cell death via STAT3 activation and IL‐1β production after status epilepticus. J Neurosci. 2017;37(33):7878‐7892. 28716963 10.1523/JNEUROSCI.0315-17.2017 PMC5559763

[98] Han R , Gu S , Zhang Y , et al. Estrogen promotes progression of hormone‐dependent breast cancer through CCL2‐CCR2 axis by upregulation of Twist via PI3K/AKT/NF‐κB signaling. Sci Rep. 2018;8(1):9575. 29934505 10.1038/s41598-018-27810-6 PMC6015029

[99] Guo F , Xu D , Lin Y , et al. Chemokine CCL2 contributes to BBB disruption via the p38 MAPK signaling pathway following acute intracerebral hemorrhage. FASEB J. 2020;34(1):1872‐1884. 31914700 10.1096/fj.201902203RR

[100] Kulbe H , Levinson NR , Balkwill F , Wilson JL . The chemokine network in cancer–much more than directing cell movement. Int J Dev Biol. 2004;48(5‐6):489‐496. 15349823 10.1387/ijdb.041814hk

[101] Yoshimura T . The chemokine MCP‐1 (CCL2) in the host interaction with cancer: a foe or ally?. Cell Mol Immunol. 2018;15(4):335‐345. 29375123 10.1038/cmi.2017.135 PMC6052833

[102] O'Connor T , Borsig L , Heikenwalder M . CCL2‐CCR2 signaling in disease pathogenesis. Endocr Metab Immune Disord Drug Targets. 2015;15(2):105‐118. 25772168 10.2174/1871530315666150316120920

[103] Bao X , Chen C , Yuan L . Triptolide attenuates neuropathic pain by regulating microglia polarization through the CCL2/CCR2 axis. Evid Based Complement Alternat Med. 2021;2021:8985721. 34691228 10.1155/2021/8985721 PMC8531820

[104] Vakilian A , Khorramdelazad H , Heidari P , Sheikh Rezaei Z , Hassanshahi G . CCL2/CCR2 signaling pathway in glioblastoma multiforme. Neurochem Int. 2017;103:1‐7. 28025034 10.1016/j.neuint.2016.12.013

[105] Liu H , Sun Y , O'Brien JA , et al. Necroptotic astrocytes contribute to maintaining stemness of disseminated medulloblastoma through CCL2 secretion. Neuro Oncol. 2020;22(5):625‐638. 31729527 10.1093/neuonc/noz214 PMC7229261

[106] Hedayati‐Moghadam M , Hosseinian S , Paseban M , et al. The role of chemokines in cardiovascular diseases and the therapeutic effect of curcumin on CXCL8 and CCL2 as pathological chemokines in atherosclerosis. Adv Exp Med Biol. 2021;1328:155‐170. 34981477 10.1007/978-3-030-73234-9_11

[107] Moadab F , Khorramdelazad H , Abbasifard M . Role of CCL2/CCR2 axis in the immunopathogenesis of rheumatoid arthritis: latest evidence and therapeutic approaches. Life Sci. 2021;269:119034. 33453247 10.1016/j.lfs.2021.119034

[108] Ding X , Yang DR , Lee SO , et al. TR4 nuclear receptor promotes prostate cancer metastasis via upregulation of CCL2/CCR2 signaling. Int J Cancer. 2015;136(4):955‐964. 24975468 10.1002/ijc.29049

[109] Fang WB , Smart C , et al. Expression of CCL2/CCR2 signaling proteins in breast carcinoma cells is associated with invasive progression. Sci Rep. 2021;11(1):8708. 33888841 10.1038/s41598-021-88229-0 PMC8062684

[110] Avila MA , Berasain C . Targeting CCL2/CCR2 in tumor‐infiltrating macrophages: a tool emerging out of the box against hepatocellular carcinoma. Cell Mol Gastroenterol Hepatol. 2019;7(2):293‐294. 30529279 10.1016/j.jcmgh.2018.11.002 PMC6354282

[111] An J , Xue Y , Long M , Zhang G , Zhang J , Su H . Targeting CCR2 with its antagonist suppresses viability, motility and invasion by downregulating MMP‐9 expression in non‐small cell lung cancer cells. Oncotarget. 2017;8(24):39230‐39240. 28424406 10.18632/oncotarget.16837 PMC5503609

[112] Wang Z , Xie H , Zhou L , et al. CCL2/CCR2 axis is associated with postoperative survival and recurrence of patients with non‐metastatic clear‐cell renal cell carcinoma. Oncotarget. 2016;7(32):51525‐51534. 27409666 10.18632/oncotarget.10492 PMC5239494

[113] Sanford DE , Belt BA , Panni RZ , et al. Inflammatory monocyte mobilization decreases patient survival in pancreatic cancer: a role for targeting the CCL2/CCR2 axis. Clin Cancer Res. 2013;19(13):3404‐3415. 23653148 10.1158/1078-0432.CCR-13-0525 PMC3700620

[114] Chen CH , Su LJ , Tsai HT , Hwang CF . ELF‐1 expression in nasopharyngeal carcinoma facilitates proliferation and metastasis of cancer cells via modulation of CCL2/CCR2 signaling. Cancer Manag Res. 2019;11:5243‐5254. 31289447 10.2147/CMAR.S196355 PMC6560358

[115] Lu Y , Cai Z , Galson DL , et al. Monocyte chemotactic protein‐1 (MCP‐1) acts as a paracrine and autocrine factor for prostate cancer growth and invasion. Prostate. 2006;66(12):1311‐1318. 16705739 10.1002/pros.20464

[116] Xu W , Wei Q , Han M , et al. CCL2‐SQSTM1 positive feedback loop suppresses autophagy to promote chemoresistance in gastric cancer. Int J Biol Sci. 2018;14(9):1054‐1066. 29989092 10.7150/ijbs.25349 PMC6036739

[117] Wang T , Zhan Q , Peng X , Qiu Z , Zhao T . CCL2 influences the sensitivity of lung cancer A549 cells to docetaxel. Oncol Lett. 2018;16(1):1267‐1274. 30061946 10.3892/ol.2018.8769 PMC6063033

[118] Chang AL , Miska J , Wainwright DA , et al. CCL2 produced by the glioma microenvironment is essential for the recruitment of regulatory T cells and myeloid‐derived suppressor cells. Cancer Res. 2016;76(19):5671‐5682. 27530322 10.1158/0008-5472.CAN-16-0144 PMC5050119

[119] Yang H , Zhang Q , Xu M , et al. CCL2‐CCR2 axis recruits tumor associated macrophages to induce immune evasion through PD‐1 signaling in esophageal carcinogenesis. Mol Cancer. 2020;19(1):41. 32103760 10.1186/s12943-020-01165-x PMC7045401

[120] Cho HR , Kumari N , Thi Vu H , Kim H , Park CK , Choi SH . Increased antiangiogenic effect by blocking CCL2‐dependent macrophages in a rodent glioblastoma model: correlation study with dynamic susceptibility contrast perfusion MRI. Sci Rep. 2019;9(1):11085. 31366997 10.1038/s41598-019-47438-4 PMC6668454

[121] Kalbasi A , Komar C , Tooker GM , et al. Tumor‐derived CCL2 mediates resistance to radiotherapy in pancreatic ductal adenocarcinoma. Clin Cancer Res. 2017;23(1):137‐148. 27354473 10.1158/1078-0432.CCR-16-0870 PMC5195913

[122] Fader AN , Rasool N , Vaziri SA , et al. CCL2 expression in primary ovarian carcinoma is correlated with chemotherapy response and survival outcomes. Anticancer Res. 2010;30(12):4791‐4798. 21187454

[123] Li L , Liu YD , Zhan YT , et al. High levels of CCL2 or CCL4 in the tumor microenvironment predict unfavorable survival in lung adenocarcinoma. Thorac Cancer. 2018;9(7):775‐784. 29722145 10.1111/1759-7714.12643 PMC6026602

[124] Lavender N , Yang J , Chen SC , et al. The Yin/Yan of CCL2: a minor role in neutrophil anti‐tumor activity in vitro but a major role on the outgrowth of metastatic breast cancer lesions in the lung in vivo. BMC Cancer. 2017;17(1):88. 28143493 10.1186/s12885-017-3074-2 PMC5286656

[125] Gutierrez‐Arcelus M , Rich SS , Raychaudhuri S . Autoimmune diseases ‐ connecting risk alleles with molecular traits of the immune system. Nat Rev Genet. 2016;17(3):160‐174. 26907721 10.1038/nrg.2015.33 PMC4896831

[126] Kraus AU , Penna‐Martinez M , Meyer G , Badenhoop K . Vitamin D effects on monocytes' CCL‐2, IL6 and CD14 transcription in Addison's disease and HLA susceptibility. J Steroid Biochem Mol Biol. 2018;177:53‐58. 28765037 10.1016/j.jsbmb.2017.07.026

[127] Rana AK , Li Y , Dang Q , Yang F . Monocytes in rheumatoid arthritis: circulating precursors of macrophages and osteoclasts and, their heterogeneity and plasticity role in RA pathogenesis. Int Immunopharmacol. 2018;65:348‐359. 30366278 10.1016/j.intimp.2018.10.016

[128] Behfar S , Hassanshahi G , Nazari A , Khorramdelazad H . A brief look at the role of monocyte chemoattractant protein‐1 (CCL2) in the pathophysiology of psoriasis. Cytokine. 2018;110:226‐231. 29277337 10.1016/j.cyto.2017.12.010

[129] Kulkarni O , Anders HJ . CCL2/MCP1: a novel target in systemic lupus erythematosus and lupus nephritis. Z Rheumatol. 2008;67(3):220‐224. 18398619 10.1007/s00393-008-0283-8

[130] Lee YH , Song GG . Urinary MCP‐1 as a biomarker for lupus nephritis: a meta‐analysis. Z Rheumatol. 2017;76(4):357‐363. 27278779 10.1007/s00393-016-0109-z

[131] Devarapu SK , Kumar Vr S , Rupanagudi KV , et al. Dual blockade of the pro‐inflammatory chemokine CCL2 and the homeostatic chemokine CXCL12 is as effective as high dose cyclophosphamide in murine proliferative lupus nephritis. Clin Immunol. 2016;169:139‐147. 27392463 10.1016/j.clim.2016.07.003

[132] Stuart MJ , Baune BT . Chemokines and chemokine receptors in mood disorders, schizophrenia, and cognitive impairment: a systematic review of biomarker studies. Neurosci Biobehav Rev. 2014;42:93‐115. 24513303 10.1016/j.neubiorev.2014.02.001

[133] Duan L , Zhang XD , Miao WY , et al. PDGFRβ cells rapidly relay inflammatory signal from the circulatory system to neurons via chemokine CCL2. Neuron. 2018;100(1):183‐200.e8. 30269986 10.1016/j.neuron.2018.08.030

[134] Georgakis MK , Malik R , Björkbacka H , et al. Circulating monocyte chemoattractant protein‐1 and risk of stroke: meta‐analysis of population‐based studies involving 17 180 Individuals. Circ Res. 2019;125(8):773‐782. 31476962 10.1161/CIRCRESAHA.119.315380 PMC6763364

[135] Dhaiban S , Al‐Ani M , Elemam NM , Maghazachi AA . Targeting chemokines and chemokine receptors in multiple sclerosis and experimental autoimmune encephalomyelitis. J Inflamm Res. 2020;13:619‐633. 33061527 10.2147/JIR.S270872 PMC7532903

[136] Gutiérrez IL , González‐Prieto M , Caso JR , García‐Bueno B , Leza JC , Madrigal JLM . Reboxetine treatment reduces neuroinflammation and neurodegeneration in the 5xFAD mouse model of Alzheimer's disease: role of CCL2. Mol Neurobiol. 2019;56(12):8628‐8642. 31297718 10.1007/s12035-019-01695-6

[137] Olson A , Coote C , Snyder‐Cappione JE , Lin N , Sagar M . HIV‐1 transcription but not intact provirus levels are associated with systemic inflammation. J Infect Dis. 2021;223(11):1934‐1942. 33075121 10.1093/infdis/jiaa657 PMC8176637

[138] Sauce D , Pourcher V , Ferry T , Boddaert J , Slama L , Allavena C . Immune activation and chronic inflammation: is there an additional effect of HIV in a geriatric population?. Medicine (Baltimore). 2021;100(17):e25678. 33907138 10.1097/MD.0000000000025678 PMC8084076

[139] Henderson LJ , Reoma LB , Kovacs JA , Nath A . Advances toward curing HIV‐1 Infection in tissue reservoirs. J Virol. 2020;94(3):e00375‐19. 10.1128/JVI.00375-19 PMC7000960 31694954

[140] Covino DA , Sabbatucci M , Fantuzzi L . The CCL2/CCR2 axis in the pathogenesis of HIV‐1 infection: a new cellular target for therapy?. Curr Drug Targets. 2016;17(1):76‐110. 26687605 10.2174/138945011701151217110917

[141] Fantuzzi L , Spadaro F , Vallanti G , et al. Endogenous CCL2 (monocyte chemotactic protein‐1) modulates human immunodeficiency virus type‐1 replication and affects cytoskeleton organization in human monocyte‐derived macrophages. Blood. 2003;102(7):2334‐2337. 12805068 10.1182/blood-2002-10-3275

[142] Ansari AW , Kamarulzaman A , Schmidt RE . Multifaceted impact of host C‐C chemokine CCL2 in the immuno‐pathogenesis of HIV‐1/M. tuberculosis co‐infection. Front Immunol. 2013;4:312. 24109479 10.3389/fimmu.2013.00312 PMC3790230

[143] Vicenzi E , Alfano M , Ghezzi S , et al. Divergent regulation of HIV‐1 replication in PBMC of infected individuals by CC chemokines: suppression by RANTES, MIP‐1alpha, and MCP‐3, and enhancement by MCP‐1. J Leukoc Biol. 2000;68(3):405‐412. 10985258

[144] Campbell GR , Spector SA . CCL2 increases X4‐tropic HIV‐1 entry into resting CD4+ T cells. J Biol Chem. 2008;283(45):30745‐30753. 18784079 10.1074/jbc.M804112200 PMC2576528

[145] Sabbatucci M , Covino DA , Purificato C , et al. Endogenous CCL2 neutralization restricts HIV‐1 replication in primary human macrophages by inhibiting viral DNA accumulation. Retrovirology. 2015;12:4. 25608886 10.1186/s12977-014-0132-6 PMC4314729

[146] Chen S , Yang L , Nilsson‐Payant B , et al. SARS‐CoV‐2 infected cardiomyocytes recruit monocytes by secreting CCL2. Res Sq. 2020. 10.21203/rs.3.rs-94634/v1

[147] Yang L , Nilsson‐Payant BE , Han Y , et al. Cardiomyocytes recruit monocytes upon SARS‐CoV‐2 infection by secreting CCL2. Stem Cell Rep. 2021;16(9):2274‐2288. 10.1016/j.stemcr.2021.07.012 PMC8289700 34403650

[148] Sandhu SK , Papadopoulos K , Fong PC , et al. A first‐in‐human, first‐in‐class, phase I study of carlumab (CNTO 888), a human monoclonal antibody against CC‐chemokine ligand 2 in patients with solid tumors. Cancer Chemother Pharmacol. 2013;71(4):1041‐1050. 23385782 10.1007/s00280-013-2099-8

[149] Pienta KJ , Machiels JP , Schrijvers D , et al. Phase 2 study of carlumab (CNTO 888), a human monoclonal antibody against CC‐chemokine ligand 2 (CCL2), in metastatic castration‐resistant prostate cancer. Invest New Drugs. 2013;31(3):760‐768. 22907596 10.1007/s10637-012-9869-8

[150] Brana I , Calles A , LoRusso PM , et al. an anti‐C‐C chemokine ligand 2 monoclonal antibody, in combination with four chemotherapy regimens for the treatment of patients with solid tumors: an open‐label, multicenter phase 1b study. Target Oncol. 2015;10(1):111‐123. 24928772 10.1007/s11523-014-0320-2

[151] Gilbert J , Lekstrom‐Himes J , Donaldson D , et al. Effect of CC chemokine receptor 2 CCR2 blockade on serum C‐reactive protein in individuals at atherosclerotic risk and with a single nucleotide polymorphism of the monocyte chemoattractant protein‐1 promoter region. Am J Cardiol. 2011;107(6):906‐911. 21247529 10.1016/j.amjcard.2010.11.005

[152] Vergunst CE , Gerlag DM , Lopatinskaya L , et al. Modulation of CCR2 in rheumatoid arthritis: a double‐blind, randomized, placebo‐controlled clinical trial. Arthritis Rheum. 2008;58(7):1931‐1939. 18576354 10.1002/art.23591

[153] Nywening TM , Wang‐Gillam A , Sanford DE , et al. Targeting tumour‐associated macrophages with CCR2 inhibition in combination with FOLFIRINOX in patients with borderline resectable and locally advanced pancreatic cancer: a single‐centre, open‐label, dose‐finding, non‐randomised, phase 1b trial. Lancet Oncol. 2016;17(5):651‐662. 27055731 10.1016/S1470-2045(16)00078-4 PMC5407285

[154] Di Prospero NA , Artis E , Andrade‐Gordon P , et al. CCR2 antagonism in patients with type 2 diabetes mellitus: a randomized, placebo‐controlled study. Diabetes Obes Metab. 2014;16(11):1055‐1064.</bib 24798870 10.1111/dom.12309

[155] Bekker P , Charvat T , Miao S , et al. Clinical development of CCR2 antagonists CCX140‐B and CCX872‐B. Nephrology Dialysis Transplantation. 50th European‐Renal‐Association, Volume 28 2013;17‐17.

[156] Horuk R . Chemokine receptor antagonists: overcoming developmental hurdles. Nat Rev Drug Discov. 2009;8(1):23‐33. 19079127 10.1038/nrd2734

[157] Yang MG , Xiao Z , Cherney RJ , et al. Use of a conformational‐switching mechanism to modulate exposed polarity: discovery of CCR2 antagonist BMS‐741672. ACS Med Chem Lett. 2019;10(3):300‐305. 30891130 10.1021/acsmedchemlett.8b00439 PMC6421546

[158] Kalliomäki J , Attal N , Jonzon B , et al. A randomized, double‐blind, placebo‐controlled trial of a chemokine receptor 2 (CCR2) antagonist in posttraumatic neuralgia. Pain. 2013;154(5):761‐767. 23523116 10.1016/j.pain.2013.02.003

[159] Kalliomäki J , Huizar K , Kågedal M , Hägglöf B , Schmelz M . Evaluation of the effects of a metabotropic glutamate receptor 5‐antagonist on electrically induced pain and central sensitization in healthy human volunteers. Eur J Pain. 2013;17(10):1465‐1471. 23650072 10.1002/j.1532-2149.2013.00327.x

[160] Appay V , Rowland‐Jones SL . RANTES: a versatile and controversial chemokine. Trends Immunol. 2001;22(2):83‐87. 11286708 10.1016/s1471-4906(00)01812-3

[161] Aldinucci D , Borghese C , Casagrande N . The CCL5/CCR5 axis in cancer progression. Cancers (Basel). 2020;12(7):1765. 10.3390/cancers12071765 PMC7407580 32630699

[162] Hemmatazad H , Berger MD . CCR5 is a potential therapeutic target for cancer. Expert Opin Ther Targets. 2021;25(4):311‐327. 33719836 10.1080/14728222.2021.1902505

[163] Oppermann M . Chemokine receptor CCR5: insights into structure, function, and regulation. Cell Signal. 2004;16(11):1201‐1210. 15337520 10.1016/j.cellsig.2004.04.007

[164] Martínez‐Muñoz L , Barroso R , Dyrhaug SY , et al. CCR5/CD4/CXCR4 oligomerization prevents HIV‐1 gp120IIIB binding to the cell surface. Proc Natl Acad Sci USA. 2014;111(19):E1960‐E1969. 24778234 10.1073/pnas.1322887111 PMC4024905

[165] Huang CY , Fong YC , Lee CY , et al. CCL5 increases lung cancer migration via PI3K, Akt and NF‐kappaB pathways. Biochem Pharmacol. 2009;77(5):794‐803. 19073147 10.1016/j.bcp.2008.11.014

[166] Hermida MA , Dinesh Kumar J , Leslie NR . GSK3 and its interactions with the PI3K/AKT/mTOR signalling network. Adv Biol Regul. 2017;65:5‐15. 28712664 10.1016/j.jbior.2017.06.003

[167] Johansen C , Rittig AH , Mose M , et al. STAT2 is involved in the pathogenesis of psoriasis by promoting CXCL11 and CCL5 production by keratinocytes. PLoS One. 2017;12(5):e0176994. 28472186 10.1371/journal.pone.0176994 PMC5417613

[168] Velasco‐Velázquez M , Jiao X , De La Fuente M , et al. CCR5 antagonist blocks metastasis of basal breast cancer cells. Cancer Res. 2012;72(15):3839‐3850. 22637726 10.1158/0008-5472.CAN-11-3917

[169] Zi J , Yuan S , Qiao J , et al. Treatment with the C‐C chemokine receptor type 5 (CCR5)‐inhibitor maraviroc suppresses growth and induces apoptosis of acute lymphoblastic leukemia cells. Am J Cancer Res. 2017;7(4):869‐880. 28469959 PMC5411794

[170] Menu E , De Leenheer E , De Raeve H , et al. Role of CCR1 and CCR5 in homing and growth of multiple myeloma and in the development of osteolytic lesions: a study in the 5TMM model. Clin Exp Metastasis. 2006;23(5‐6):291‐300. 17086356 10.1007/s10585-006-9038-6

[171] Casagrande N , Borghese C , Visser L , Mongiat M , Colombatti A , Aldinucci D . CCR5 antagonism by maraviroc inhibits Hodgkin lymphoma microenvironment interactions and xenograft growth. Haematologica. 2019;104(3):564‐575. 30309853 10.3324/haematol.2018.196725 PMC6395337

[172] Halama N , Zoernig I , Berthel A , et al. Tumoral immune cell exploitation in colorectal cancer metastases can be targeted effectively by anti‐CCR5 therapy in cancer patients. Cancer Cell. 2016;29(4):587‐601. 27070705 10.1016/j.ccell.2016.03.005

[173] Velasco‐Velázquez M , Xolalpa W , Pestell RG . The potential to target CCL5/CCR5 in breast cancer. Expert Opin Ther Targets. 2014;18(11):1265‐1275. 25256399 10.1517/14728222.2014.949238

[174] Khalid A , Wolfram J , Ferrari I , et al. Recent advances in discovering the role of CCL5 in metastatic breast cancer. Mini Rev Med Chem. 2015;15(13):1063‐1072. 26420723 10.2174/138955751513150923094709 PMC4968951

[175] Karnoub AE , Dash AB , Vo AP , et al. Mesenchymal stem cells within tumour stroma promote breast cancer metastasis. Nature. 2007;449(7162):557‐563. 17914389 10.1038/nature06188

[176] Yazdani Z , Mousavi Z , Ghasemimehr N , et al. Differential regulatory effects of chemotherapeutic protocol on CCL3_CCL4_CCL5/CCR5 axes in acute myeloid leukemia patients with monocytic lineage. Life Sci. 2020;240:117071. 31783051 10.1016/j.lfs.2019.117071

[177] Waldeck S , Rassner M , Keye P , et al. CCL5 mediates target‐kinase independent resistance to FLT3 inhibitors in FLT3‐ITD‐positive AML. Mol Oncol. 2020;14(4):779‐794. 31955503 10.1002/1878-0261.12640 PMC7138400

[178] Aldinucci D , Lorenzon D , Cattaruzza L , et al. Expression of CCR5 receptors on Reed‐Sternberg cells and Hodgkin lymphoma cell lines: involvement of CCL5/Rantes in tumor cell growth and microenvironmental interactions. Int J Cancer. 2008;122(4):769‐776. 17935139 10.1002/ijc.23119

[179] Abe M , Hiura K , Wilde J , et al. Role for macrophage inflammatory protein (MIP)‐1alpha and MIP‐1beta in the development of osteolytic lesions in multiple myeloma. Blood. 2002;100(6):2195‐2202. 12200385

[180] Oba Y , Lee JW , Ehrlich LA , et al. MIP‐1alpha utilizes both CCR1 and CCR5 to induce osteoclast formation and increase adhesion of myeloma cells to marrow stromal cells. Exp Hematol. 2005;33(3):272‐278. 15730850 10.1016/j.exphem.2004.11.015

[181] Cambien B , Richard‐Fiardo P , Karimdjee BF , et al. CCL5 neutralization restricts cancer growth and potentiates the targeting of PDGFRβ in colorectal carcinoma. PLoS One. 2011;6(12):e28842. 22205974 10.1371/journal.pone.0028842 PMC3243667

[182] Aldinucci D , Casagrande N . Inhibition of the CCL5/CCR5 axis against the progression of gastric cancer. Int J Mol Sci. 2018;19(5):1477. 10.3390/ijms19051477 PMC5983686 29772686

[183] González‐Arriagada WA , Lozano‐Burgos C , Zúñiga‐Moreta R , González‐Díaz P , Coletta RD . Clinicopathological significance of chemokine receptor (CCR1, CCR3, CCR4, CCR5, CCR7 and CXCR4) expression in head and neck squamous cell carcinomas. J Oral Pathol Med. 2018;47(8):755‐763. 29797610 10.1111/jop.12736

[184] Lu Y , Luan XR . miR‐147a suppresses the metastasis of non‐small‐cell lung cancer by targeting CCL5. J Int Med Res. 2020;48(4):300060519883098. 31884861 10.1177/0300060519883098 PMC7607764

[185] Long H , Xie R , Xiang T , et al. Autocrine CCL5 signaling promotes invasion and migration of CD133+ ovarian cancer stem‐like cells via NF‐κB‐mediated MMP‐9 upregulation. Stem Cells. 2012;30(10):2309‐2319. 22887854 10.1002/stem.1194

[186] Chang LY , Lin YC , Mahalingam J , et al. Tumor‐derived chemokine CCL5 enhances TGF‐β‐mediated killing of CD8(+) T cells in colon cancer by T‐regulatory cells. Cancer Res. 2012;72(5):1092‐1102. 22282655 10.1158/0008-5472.CAN-11-2493

[187] Zeng Z , Lan T , Wei Y , Wei X . CCL5/CCR5 axis in human diseases and related treatments. Genes Dis. 2022;9(1):12‐27. 34514075 10.1016/j.gendis.2021.08.004 PMC8423937

[188] Poupel L , Combadière C . Atherosclerosis : on the trail of chemokines. Biol Aujourdhui. 2010;204(4):285‐293. 21215245 10.1051/jbio/2010026

[189] Kang H , Li X , Xiong K , et al. The entry and egress of monocytes in atherosclerosis: a biochemical and biomechanical driven process. Cardiovasc Ther. 2021;2021:6642927. 34345249 10.1155/2021/6642927 PMC8282391

[190] Jongstra‐Bilen J , Tai K , Althagafi MG , et al. Role of myeloid‐derived chemokine CCL5/RANTES at an early stage of atherosclerosis. J Mol Cell Cardiol. 2021;156:69‐78. 33781821 10.1016/j.yjmcc.2021.03.010

[191] Li Y , Liu X , Duan W , et al. Batf3‐dependent CD8α(+) dendritic cells aggravates atherosclerosis via Th1 cell induction and enhanced CCL5 expression in plaque macrophages. EBioMedicine. 2017;18:188‐198. 28411140 10.1016/j.ebiom.2017.04.008 PMC5405198

[192] Chistiakov DA , Melnichenko AA , Grechko AV , Myasoedova VA , Orekhov AN . Potential of anti‐inflammatory agents for treatment of atherosclerosis. Exp Mol Pathol. 2018;104(2):114‐124. 29378168 10.1016/j.yexmp.2018.01.008

[193] Mencarelli A , Cipriani S , Francisci D , et al. Highly specific blockade of CCR5 inhibits leukocyte trafficking and reduces mucosal inflammation in murine colitis. Sci Rep. 2016;6:30802. 27492684 10.1038/srep30802 PMC4974621

[194] Wen Y , Lambrecht J , Ju C , Tacke F . Hepatic macrophages in liver homeostasis and diseases‐diversity, plasticity and therapeutic opportunities. Cell Mol Immunol. 2021;18(1):45‐56. 33041338 10.1038/s41423-020-00558-8 PMC7852525

[195] Luedde T , Schwabe RF . NF‐κB in the liver–linking injury, fibrosis and hepatocellular carcinoma. Nat Rev Gastroenterol Hepatol. 2011;8(2):108‐118. 21293511 10.1038/nrgastro.2010.213 PMC3295539

[196] Li M , Sun X , Zhao J , et al. CCL5 deficiency promotes liver repair by improving inflammation resolution and liver regeneration through M2 macrophage polarization. Cell Mol Immunol. 2020;17(7):753‐764. 31481754 10.1038/s41423-019-0279-0 PMC7331700

[197] Patterson BK , Seethamraju H , Dhody K , et al. Disruption of the CCL5/RANTES‐CCR5 pathway restores immune homeostasis and reduces plasma viral load in critical COVID‐19. medRxiv. 2020. 10.1101/2020.05.02.20084673

[198] Agresti N , Lalezari JP , Amodeo PP , et al. Disruption of CCR5 signaling to treat COVID‐19‐associated cytokine storm: case series of four critically ill patients treated with leronlimab. J Transl Autoimmun. 2021;4:100083. 33521616 10.1016/j.jtauto.2021.100083 PMC7823045

[199] Kuwata T , Enomoto I , Baba M , Matsushita S . Incompatible natures of the HIV‐1 envelope in resistance to the CCR5 antagonist cenicriviroc and to neutralizing antibodies. Antimicrob Agents Chemother. 2016;60(1):437‐450. 26525792 10.1128/AAC.02285-15 PMC4704143

[200] Visseaux B , Charpentier C , Collin G , et al. Cenicriviroc, a novel CCR5 (R5) and CCR2 antagonist, shows in vitro activity against R5 tropic HIV‐2 clinical isolates. PLoS One. 2015;10(8):e0134904. 26247470 10.1371/journal.pone.0134904 PMC4527700

[201] Nitta T , Nitta S , Lei Y , Lipp M , Takahama Y . CCR7‐mediated migration of developing thymocytes to the medulla is essential for negative selection to tissue‐restricted antigens. Proc Natl Acad Sci USA. 2009;106(40):17129‐17133. 19805112 10.1073/pnas.0906956106 PMC2761327

[202] Van Raemdonck K , Umar S , Palasiewicz K , et al. CCL21/CCR7 signaling in macrophages promotes joint inflammation and Th17‐mediated osteoclast formation in rheumatoid arthritis. Cell Mol Life Sci. 2020;77(7):1387‐1399. 31342120 10.1007/s00018-019-03235-w PMC10040247

[203] Jaeger K , Bruenle S , Weinert T , et al. Structural basis for allosteric ligand recognition in the human CC chemokine receptor 7. Cell. 2019;178(5):1222‐1230.e10. 31442409 10.1016/j.cell.2019.07.028 PMC6709783

[204] Zheng Y , Manzotti CN , Liu M , Burke F , Mead KI , Sansom DM . CD86 and CD80 differentially modulate the suppressive function of human regulatory T cells. J Immunol. 2004;172(5):2778‐2784. 14978077 10.4049/jimmunol.172.5.2778

[205] Haessler U , Pisano M , Wu M , Swartz MA . Dendritic cell chemotaxis in 3D under defined chemokine gradients reveals differential response to ligands CCL21 and CCL19. Proc Natl Acad Sci USA. 2011;108(14):5614‐5619. 21422278 10.1073/pnas.1014920108 PMC3078419

[206] Ricart BG , John B , Lee D , Hunter CA , Hammer DA . Dendritic cells distinguish individual chemokine signals through CCR7 and CXCR4. J Immunol. 2011;186(1):53‐61. 21106854 10.4049/jimmunol.1002358

[207] Correale P , Rotundo MS , Botta C , et al. Tumor infiltration by T lymphocytes expressing chemokine receptor 7 (CCR7) is predictive of favorable outcome in patients with advanced colorectal carcinoma. Clin Cancer Res. 2012;18(3):850‐857. 22142823 10.1158/1078-0432.CCR-10-3186

[208] Aggarwal S , Sharma SC , S ND . Dynamics of regulatory T cells (T(regs)) in patients with oral squamous cell carcinoma. J Surg Oncol. 2017;116(8):1103‐1113. 28833201 10.1002/jso.24782

[209] Zhang L , Wang F , Yao X , Ma S , Zhang L , Qin Z . Progress in targeting therapy of cancer metastasis by CCL21/CCR7 axis. Sheng Wu Gong Cheng Xue Bao. 2020;36(12):2741‐2754. 33398969 10.13345/j.cjb.200174

[210] Hillinger S , Yang SC , Batra RK , et al. CCL19 reduces tumour burden in a model of advanced lung cancer. Br J Cancer. 2006;94(7):1029‐1034. 16598185 10.1038/sj.bjc.6603061 PMC2361223

[211] Lu J , Ma J , Cai W , et al. CC motif chemokine ligand 19 suppressed colorectal cancer in vivo accompanied by an increase in IL‐12 and IFN‐γ. Biomed Pharmacother. 2015;69:374‐379. 25661385 10.1016/j.biopha.2014.12.032

[212] Phan‐Lai V , Kievit FM , Florczyk SJ , Wang K , Disis ML , Zhang M . CCL21 and IFNγ recruit and activate tumor specific T cells in 3D scaffold model of breast cancer. Anticancer Agents Med Chem. 2014;14(2):204‐210. 24237220 10.2174/18715206113136660375 PMC4049463

[213] Gao JQ , Sugita T , Kanagawa N , et al. Anti‐tumor responses induced by chemokine CCL19 transfected into an ovarian carcinoma model via fiber‐mutant adenovirus vector. Biol Pharm Bull. 2005;28(6):1066‐1070. 15930746 10.1248/bpb.28.1066

[214] Hamanishi J , Mandai M , Matsumura N , et al. Activated local immunity by CC chemokine ligand 19‐transduced embryonic endothelial progenitor cells suppresses metastasis of murine ovarian cancer. Stem Cells. 2010;28(1):164‐173. 19911426 10.1002/stem.256

[215] Wu S , Xing W , Peng J , et al. Tumor transfected with CCL21 enhanced reactivity and apoptosis resistance of human monocyte‐derived dendritic cells. Immunobiology. 2008;213(5):417‐426. 18472050 10.1016/j.imbio.2007.10.003

[216] Igoucheva O , Grazzini M , Pidich A , et al. Immunotargeting and eradication of orthotopic melanoma using a chemokine‐enhanced DNA vaccine. Gene Ther. 2013;20(9):939‐948. 23552473 10.1038/gt.2013.17

[217] Iida Y , Yoshikawa R , Murata A , et al. Local injection of CCL19‐expressing mesenchymal stem cells augments the therapeutic efficacy of anti‐PD‐L1 antibody by promoting infiltration of immune cells. J Immunother Cancer. 2020;8(2):e000582. 10.1136/jitc-2020-000582 PMC7368496 32675195

[218] Komatsu N , Takayanagi H . Autoimmune arthritis: the interface between the immune system and joints. Adv Immunol. 2012;115:45‐71. 22608255 10.1016/B978-0-12-394299-9.00002-3

[219] Page G , Lebecque S , Miossec P . Anatomic localization of immature and mature dendritic cells in an ectopic lymphoid organ: correlation with selective chemokine expression in rheumatoid synovium. J Immunol. 2002;168(10):5333‐5341. 11994492 10.4049/jimmunol.168.10.5333

[220] Mitsui H , Suárez‐Fariñas M , Belkin DA , et al. Combined use of laser capture microdissection and cDNA microarray analysis identifies locally expressed disease‐related genes in focal regions of psoriasis vulgaris skin lesions. J Invest Dermatol. 2012;132(6):1615‐1626. 22402443 10.1038/jid.2012.33 PMC3352975

[221] de Graaf MT , Smitt PA , Luitwieler RL , et al. Central memory CD4+ T cells dominate the normal cerebrospinal fluid. Cytometry B Clin Cytom. 2011;80(1):43‐50. 20632412 10.1002/cyto.b.20542

[222] Gold R , Jawad A , Miller DH , et al. Expert opinion: guidelines for the use of natalizumab in multiple sclerosis patients previously treated with immunomodulating therapies. J Neuroimmunol. 2007;187(1‐2):156‐158. 17499366 10.1016/j.jneuroim.2007.04.006

[223] Alt C , Laschinger M , Engelhardt B . Functional expression of the lymphoid chemokines CCL19 (ELC) and CCL 21 (SLC) at the blood‐brain barrier suggests their involvement in G‐protein‐dependent lymphocyte recruitment into the central nervous system during experimental autoimmune encephalomyelitis. Eur J Immunol. 2002;32(8):2133‐2144. 12209625 10.1002/1521-4141(200208)32:8<2133::AID-IMMU2133>3.0.CO;2-W

[224] Columba‐Cabezas S , Serafini B , Ambrosini E , Aloisi F . Lymphoid chemokines CCL19 and CCL21 are expressed in the central nervous system during experimental autoimmune encephalomyelitis: implications for the maintenance of chronic neuroinflammation. Brain Pathol. 2003;13(1):38‐51. 12580544 10.1111/j.1750-3639.2003.tb00005.x PMC8095989

[225] Lee AY , Eri R , Lyons AB , Grimm MC , Korner H . CC chemokine ligand 20 and its cognate receptor CCR6 in mucosal T cell immunology and inflammatory bowel disease: odd couple or axis of evil?. Front Immunol. 2013;4:194. 23874340 10.3389/fimmu.2013.00194 PMC3711275

[226] Mita S , Nakakuki M , Ichioka M , et al. Dienogest inhibits C‐C motif chemokine ligand 20 expression in human endometriotic epithelial cells. Eur J Obstet Gynecol Reprod Biol. 2017;214:65‐70. 28482330 10.1016/j.ejogrb.2017.04.048

[227] Chen W , Qin Y , Liu S . CCL20 signaling in the tumor microenvironment. Adv Exp Med Biol. 2020;1231:53‐65. 32060846 10.1007/978-3-030-36667-4_6

[228] Fujiie S , Hieshima K , Izawa D , et al. Proinflammatory cytokines induce liver and activation‐regulated chemokine/macrophage inflammatory protein‐3alpha/CCL20 in mucosal epithelial cells through NF‐kappaB [correction of NK‐kappaB]. Int Immunol. 2001;13(10):1255‐1263. 11581170 10.1093/intimm/13.10.1255

[229] Skovdahl HK , Damås JK , Granlund AVB , et al. C‐C motif ligand 20 (CCL20) and C‐C motif chemokine receptor 6 (CCR6) in human peripheral blood mononuclear cells: dysregulated in ulcerative colitis and a potential role for CCL20 in IL‐1β release. Int J Mol Sci. 2018;19(10):3257. 10.3390/ijms19103257 PMC6214005 30347808

[230] Paradis M , Mindt BC , Duerr CU , et al. A TNF‐α‐CCL20‐CCR6 axis regulates Nod1‐induced B cell responses. J Immunol. 2014;192(6):2787‐2799. 24534531 10.4049/jimmunol.1203310

[231] Baba M , Imai T , Nishimura M , et al. Identification of CCR6, the specific receptor for a novel lymphocyte‐directed CC chemokine LARC. J Biol Chem. 1997;272(23):14893‐14898. 9169459 10.1074/jbc.272.23.14893

[232] Yang D , Chertov O , Bykovskaia SN , et al. Beta‐defensins: linking innate and adaptive immunity through dendritic and T cell CCR6. Science. 1999;286(5439):525‐528. 10521347 10.1126/science.286.5439.525

[233] Lee AYS , Körner H . The CCR6‐CCL20 axis in humoral immunity and T‐B cell immunobiology. Immunobiology. 2019;224(3):449‐454. 30772094 10.1016/j.imbio.2019.01.005

[234] Meitei HT , Jadhav N , Lal G . CCR6‐CCL20 axis as a therapeutic target for autoimmune diseases. Autoimmun Rev. 2021;20(7):102846. 33971346 10.1016/j.autrev.2021.102846

[235] Ding X , Wang K , Wang H , et al. High expression of CCL20 is associated with poor prognosis in patients with hepatocellular carcinoma after curative resection. J Gastrointest Surg. 2012;16(4):828‐836. 22072303 10.1007/s11605-011-1775-4

[236] Lee SK , Park KK , Kim HJ , et al. Human antigen R‐regulated CCL20 contributes to osteolytic breast cancer bone metastasis. Sci Rep. 2017;7(1):9610. 28851919 10.1038/s41598-017-09040-4 PMC5575024

[237] Wang D , Yuan W , Wang Y , et al. Serum CCL20 combined with IL‐17A as early diagnostic and prognostic biomarkers for human colorectal cancer. J Transl Med. 2019;17(1):253. 31387598 10.1186/s12967-019-2008-y PMC6685266

[238] Wang D , Yang L , Yu W , et al. Colorectal cancer cell‐derived CCL20 recruits regulatory T cells to promote chemoresistance via FOXO1/CEBPB/NF‐κB signaling. J Immunother Cancer. 2019;7(1):215. 31395078 10.1186/s40425-019-0701-2 PMC6688336

[239] Liu B , Jia Y , Ma J , et al. Tumor‐associated macrophage‐derived CCL20 enhances the growth and metastasis of pancreatic cancer. Acta Biochim Biophys Sin (Shanghai). 2016;48(12):1067‐1074. 27797715 10.1093/abbs/gmw101

[240] Zhang XP , Hu ZJ , Meng AH , Duan GC , Zhao QT , Yang J . Role of CCL20/CCR6 and the ERK signaling pathway in lung adenocarcinoma. Oncol Lett. 2017;14(6):8183‐8189. 29250193 10.3892/ol.2017.7253 PMC5727607

[241] Walch‐Rückheim B , Mavrova R , Henning M , et al. Stromal fibroblasts induce CCL20 through IL6/C/EBPβ to support the recruitment of Th17 cells during cervical cancer progression. Cancer Res. 2015;75(24):5248‐5259. 26631268 10.1158/0008-5472.CAN-15-0732

[242] Han G , Wu D , Yang Y , Li Z , Zhang J , Li C . CrkL meditates CCL20/CCR6‐induced EMT in gastric cancer. Cytokine. 2015;76(2):163‐169. 26044596 10.1016/j.cyto.2015.05.009

[243] Liu W , Wang W , Wang X , Xu C , Zhang N , Di W . Cisplatin‐stimulated macrophages promote ovarian cancer migration via the CCL20‐CCR6 axis. Cancer Lett. 2020;472:59‐69. 31866467 10.1016/j.canlet.2019.12.024

[244] Kadomoto S , Izumi K , Hiratsuka K , et al. Tumor‐associated macrophages induce migration of renal cell carcinoma cells via activation of the CCL20‐CCR6 axis. Cancers (Basel). 2019;12(1):89. 10.3390/cancers12010089 PMC7017081 31905918

[245] Harper EG , Guo C , Rizzo H , et al. Th17 cytokines stimulate CCL20 expression in keratinocytes in vitro and in vivo: implications for psoriasis pathogenesis. J Invest Dermatol. 2009;129(9):2175‐2183. 19295614 10.1038/jid.2009.65 PMC2892172

[246] Kim TG , Jee H , Fuentes‐Duculan J , et al. Dermal clusters of mature dendritic cells and T cells are associated with the CCL20/CCR6 chemokine system in chronic psoriasis. J Invest Dermatol. 2014;134(5):1462‐1465. 24401998 10.1038/jid.2013.534

[247] Brunner PM , Koszik F , Reininger B , Kalb ML , Bauer W , Stingl G . Infliximab induces downregulation of the IL‐12/IL‐23 axis in 6‐sulfo‐LacNac (slan)+ dendritic cells and macrophages. J Allergy Clin Immunol. 2013;132(5):1184‐1193.e8. 23890755 10.1016/j.jaci.2013.05.036

[248] Robert R , Ang C , Sun G , et al. Essential role for CCR6 in certain inflammatory diseases demonstrated using specific antagonist and knockin mice. JCI Insight. 2017;2(15):e94821. 10.1172/jci.insight.94821 PMC5543919 28768901

[249] Mabuchi T , Singh TP , Takekoshi T , et al. CCR6 is required for epidermal trafficking of γδ‐T cells in an IL‐23‐induced model of psoriasiform dermatitis. J Invest Dermatol. 2013;133(1):164‐171. 22895364 10.1038/jid.2012.260 PMC3511632

[250] Ruth JH , Shahrara S , Park CC , et al. Role of macrophage inflammatory protein‐3alpha and its ligand CCR6 in rheumatoid arthritis. Lab Invest. 2003;83(4):579‐588. 12695561 10.1097/01.lab.0000062854.30195.52

[251] Hirota K , Yoshitomi H , Hashimoto M , et al. Preferential recruitment of CCR6‐expressing Th17 cells to inflamed joints via CCL20 in rheumatoid arthritis and its animal model. J Exp Med. 2007;204(12):2803‐2812. 18025126 10.1084/jem.20071397 PMC2118525

[252] Hou L , Yuki K . CCR6 and CXCR6 identify the Th17 cells with cytotoxicity in experimental autoimmune encephalomyelitis. Front Immunol. 2022;13:819224. 35178050 10.3389/fimmu.2022.819224 PMC8844514

[253] Elhofy A , Depaolo RW , Lira SA , Lukacs NW , Karpus WJ . Mice deficient for CCR6 fail to control chronic experimental autoimmune encephalomyelitis. J Neuroimmunol. 2009;213(1‐2):91‐99. 19535153 10.1016/j.jneuroim.2009.05.011 PMC2728039

[254] Skovdahl HK , Granlund A , Østvik AE , et al. Expression of CCL20 and its corresponding receptor CCR6 is enhanced in active inflammatory bowel disease, and TLR3 mediates CCL20 expression in colonic epithelial cells. PLoS One. 2015;10(11):e0141710. 26536229 10.1371/journal.pone.0141710 PMC4633243

[255] Godefroy E , Alameddine J , Montassier E , et al. Expression of CCR6 and CXCR6 by gut‐derived CD4(+)/CD8α(+) T‐regulatory cells, which are decreased in blood samples from patients with inflammatory bowel diseases. Gastroenterology. 2018;155(4):1205‐1217. 29981781 10.1053/j.gastro.2018.06.078

[256] Mathur AN , Zirak B , Boothby IC , et al. Treg‐cell control of a CXCL5‐IL‐17 inflammatory axis promotes hair‐follicle‐stem‐cell differentiation during skin‐barrier repair. Immunity. 2019;50(3):655‐667.e4. 30893588 10.1016/j.immuni.2019.02.013 PMC6507428

[257] Duchene J , Lecomte F , Ahmed S , et al. A novel inflammatory pathway involved in leukocyte recruitment: role for the kinin B1 receptor and the chemokine CXCL5. J Immunol. 2007;179(7):4849‐4856. 17878384 10.4049/jimmunol.179.7.4849 PMC3696729

[258] Rousselle A , Qadri F , Leukel L , et al. CXCL5 limits macrophage foam cell formation in atherosclerosis. J Clin Invest. 2013;123(3):1343‐1347. 23376791 10.1172/JCI66580 PMC3582141

[259] Persson T , Monsef N , Andersson P , et al. Expression of the neutrophil‐activating CXC chemokine ENA‐78/CXCL5 by human eosinophils. Clin Exp Allergy. 2003;33(4):531‐537. 12680872 10.1046/j.1365-2222.2003.01609.x

[260] Peng Y , Kajiyama H , Yuan H , et al. PAI‐1 secreted from metastatic ovarian cancer cells triggers the tumor‐promoting role of the mesothelium in a feedback loop to accelerate peritoneal dissemination. Cancer Lett. 2019;442:181‐192. 30429105 10.1016/j.canlet.2018.10.027

[261] Xu WW , Li B , Guan XY , et al. Cancer cell‐secreted IGF2 instigates fibroblasts and bone marrow‐derived vascular progenitor cells to promote cancer progression. Nat Commun. 2017;8:14399. 28186102 10.1038/ncomms14399 PMC5309924

[262] Yu PF , Huang Y , Han YY , et al. TNFα‐activated mesenchymal stromal cells promote breast cancer metastasis by recruiting CXCR2(+) neutrophils. Oncogene. 2017;36(4):482‐490. 27375023 10.1038/onc.2016.217 PMC5290040

[263] Yildirim K , Colak E , Aktimur R , et al. Clinical value of CXCL5 for determining of colorectal cancer. Asian Pac J Cancer Prev. 2018;19(9):2481‐2484. 30255816 10.22034/APJCP.2018.19.9.2481 PMC6249465

[264] Zhao J , Ou B , Han D , et al. Tumor‐derived CXCL5 promotes human colorectal cancer metastasis through activation of the ERK/Elk‐1/Snail and AKT/GSK3β/β‐catenin pathways. Mol Cancer. 2017;16(1):70. 28356111 10.1186/s12943-017-0629-4 PMC5372323

[265] Wang L , Shi L , Gu J , et al. CXCL5 regulation of proliferation and migration in human non‐small cell lung cancer cells. J Physiol Biochem. 2018;74(2):313‐324. 29526026 10.1007/s13105-018-0619-z

[266] Romero‐Moreno R , Curtis KJ , Coughlin TR , et al. The CXCL5/CXCR2 axis is sufficient to promote breast cancer colonization during bone metastasis. Nat Commun. 2019;10(1):4404. 31562303 10.1038/s41467-019-12108-6 PMC6765048

[267] Wang W , Zhang M , Huang Z , et al. Knockdown of CXCL5 inhibits the invasion, metastasis and stemness of bladder cancer lung metastatic cells by downregulating CD44. Anticancer Drugs. 2022;33(1):e103‐e112. 34407043 10.1097/CAD.0000000000001153 PMC8670357

[268] Qiu WZ , Zhang HB , Xia WX , et al. The CXCL5/CXCR2 axis contributes to the epithelial‐mesenchymal transition of nasopharyngeal carcinoma cells by activating ERK/GSK‐3β/snail signalling. J Exp Clin Cancer Res. 2018;37(1):85. 29665837 10.1186/s13046-018-0722-6 PMC5905166

[269] Zhou SL , Dai Z , Zhou ZJ , et al. CXCL5 contributes to tumor metastasis and recurrence of intrahepatic cholangiocarcinoma by recruiting infiltrative intratumoral neutrophils. Carcinogenesis. 2014;35(3):597‐605. 24293410 10.1093/carcin/bgt397

[270] Soler‐Cardona A , Forsthuber A , Lipp K , et al. CXCL5 facilitates melanoma cell‐neutrophil interaction and lymph node metastasis. J Invest Dermatol. 2018;138(7):1627‐1635. 29474942 10.1016/j.jid.2018.01.035

[271] Li X , Wang M , Gong T , et al. A S100A14‐CCL2/CXCL5 signaling axis drives breast cancer metastasis. Theranostics. 2020;10(13):5687‐5703. 32483412 10.7150/thno.42087 PMC7255008

[272] Najjar YG , Rayman P , Jia X , et al. Myeloid‐derived suppressor cell subset accumulation in renal cell carcinoma parenchyma is associated with intratumoral expression of IL1β, IL8, CXCL5, and Mip‐1α. Clin Cancer Res. 2017;23(9):2346‐2355. 27799249 10.1158/1078-0432.CCR-15-1823 PMC5411325

[273] Jiang SH , Zhu LL , Zhang M , et al. GABRP regulates chemokine signalling, macrophage recruitment and tumour progression in pancreatic cancer through tuning KCNN4‐mediated Ca(2+) signalling in a GABA‐independent manner. Gut. 2019;68(11):1994‐2006. 30826748 10.1136/gutjnl-2018-317479

[274] Huang Z , Zhang M , Chen G , et al. Bladder cancer cells interact with vascular endothelial cells triggering EGFR signals to promote tumor progression. Int J Oncol. 2019;54(5):1555‐1566. 30816487 10.3892/ijo.2019.4729 PMC6438427

[275] Guan Z , Li C , Fan J , He D , Li L . Androgen receptor (AR) signaling promotes RCC progression via increased endothelial cell proliferation and recruitment by modulating AKT → NF‐κB → CXCL5 signaling. Sci Rep. 2016;6:37085. 27848972 10.1038/srep37085 PMC5111066

[276] Chen C , Xu ZQ , Zong YP , et al. CXCL5 induces tumor angiogenesis via enhancing the expression of FOXD1 mediated by the AKT/NF‐κB pathway in colorectal cancer. Cell Death Dis. 2019;10(3):178. 30792394 10.1038/s41419-019-1431-6 PMC6385313

[277] Qi Y , Zhao W , Li M , et al. High C‐X‐C motif chemokine 5 expression is associated with malignant phenotypes of prostate cancer cells via autocrine and paracrine pathways. Int J Oncol. 2018;53(1):358‐370. 29749439 10.3892/ijo.2018.4388

[278] Bai L , Yao N , Qiao G , Wu L , Ma X . CXCL5 contributes to the tumorigenicity of cervical cancer and is post‐transcriptionally regulated by miR‐577. Int J Clin Exp Pathol. 2020;13(12):2984‐2993. 33425099 PMC7791384

[279] Yang Y , Hou J , Shao M , et al. CXCL5 as an autocrine or paracrine cytokine is associated with proliferation and migration of hepatoblastoma HepG2 cells. Oncol Lett. 2017;14(6):7977‐7985. 29344240 10.3892/ol.2017.7236 PMC5755189

[280] Dang H , Wu W , Wang B , et al. CXCL5 plays a promotingrRole in osteosarcoma cell migration and invasion in autocrine‐ and paracrine‐dependent manners. Oncol Res. 2017;25(2):177‐186. 28277189 10.3727/096504016X14732772150343 PMC7840695

[281] Cui D , Zhao Y , Xu J . Activation of CXCL5‐CXCR2 axis promotes proliferation and accelerates G1 to S phase transition of papillary thyroid carcinoma cells and activates JNK and p38 pathways. Cancer Biol Ther. 2019;20(5):608‐616. 30404567 10.1080/15384047.2018.1539289 PMC6606038

[282] Lee BS , Jang JY , Seo C , Kim CH . Crosstalk between head and neck cancer cells and lymphatic endothelial cells promotes tumor metastasis via CXCL5‐CXCR2 signaling. FASEB J. 2021;35(1):e21181. 33231340 10.1096/fj.202001455R

[283] Li Y , Wu T , Gong S , et al. Analysis of the prognosis and therapeutic value of the CXC chemokine family in head and neck squamous cell carcinoma. Front Oncol. 2020;10:570736. 33489879 10.3389/fonc.2020.570736 PMC7820708

[284] Jia X , Wei S , Xiong W . CXCL5/NF‐κB pathway as a therapeutic target in hepatocellular carcinoma treatment. J Oncol. 2021;2021:9919494. 34194499 10.1155/2021/9919494 PMC8184336

[285] Nie Y , Jiang MC , Liu C , Liu Q , Zhu X . CXCL5 has potential to be a marker for hepatocellular carcinoma prognosis and was correlating with immune infiltrates. Front Oncol. 2021;11:637023. 33869023 10.3389/fonc.2021.637023 PMC8045759

[286] Dawes JM , Calvo M , Perkins JR , et al. CXCL5 mediates UVB irradiation‐induced pain. Sci Transl Med. 2011;3(90):90ra60. 10.1126/scitranslmed.3002193 PMC3232447 21734176

[287] Xu W , Zhu M , Yuan S , Yu W . Spinal CXCL5 contributes to nerve injury‐induced neuropathic pain via modulating GSK‐3β phosphorylation and activity in rats. Neurosci Lett. 2016;634:52‐59. 27717828 10.1016/j.neulet.2016.10.004

[288] Liu YF , Liang JJ , Ng TK , et al. CXCL5/CXCR2 modulates inflammation‐mediated neural repair after optic nerve injury. Exp Neurol. 2021;341:113711. 33785307 10.1016/j.expneurol.2021.113711

[289] Quinton LJ , Nelson S , Zhang P , Happel KI , Gamble L , Bagby GJ . Effects of systemic and local CXC chemokine administration on the ethanol‐induced suppression of pulmonary neutrophil recruitment. Alcohol Clin Exp Res. 2005;29(7):1198‐1205. 16046875 10.1097/01.alc.0000171927.66130.aa

[290] Pedersen F , Waschki B , Marwitz S , et al. Neutrophil extracellular trap formation is regulated by CXCR2 in COPD neutrophils. Eur Respir J. 2018;51(4):1700970. 10.1183/13993003.00970-2017 29449427

[291] Sokulsky LA , Garcia‐Netto K , Nguyen TH , et al. A critical role for the CXCL3/CXCL5/CXCR2 neutrophilic chemotactic axis in the regulation of type 2 responses in a model of rhinoviral‐induced asthma exacerbation. J Immunol. 2020;205(9):2468‐2478. 32948685 10.4049/jimmunol.1901350

[292] Wang CY , Shang M , Zhou CL , Feng LZ , Zhou QS , Hu K . Mechanism of Cxc chemokine ligand 5 (CXCL5)/Cxc chemokine receptor 2 (CXCR2) bio‐axis in mice with acute respiratory distress syndrome. Med Sci Monit. 2019;25:5299‐5305. 31311916 10.12659/MSM.915835 PMC6659456

[293] Jacobs JP , Ortiz‐Lopez A , Campbell JJ , Gerard CJ , Mathis D , Benoist C . Deficiency of CXCR2, but not other chemokine receptors, attenuates autoantibody‐mediated arthritis in a murine model. Arthritis Rheum. 2010;62(7):1921‐1932. 20506316 10.1002/art.27470 PMC2994550

[294] Barsante MM , Cunha TM , Allegretti M , et al. Blockade of the chemokine receptor CXCR2 ameliorates adjuvant‐induced arthritis in rats. Br J Pharmacol. 2008;153(5):992‐1002. 17891165 10.1038/sj.bjp.0707462 PMC2267272

[295] Podolin PL , Bolognese BJ , Foley JJ , et al. A potent and selective nonpeptide antagonist of CXCR2 inhibits acute and chronic models of arthritis in the rabbit. J Immunol. 2002;169(11):6435‐6444. 12444152 10.4049/jimmunol.169.11.6435

[296] Ning Y , Labonte MJ , Zhang W , et al. The CXCR2 antagonist, SCH‐527123, shows antitumor activity and sensitizes cells to oxaliplatin in preclinical colon cancer models. Mol Cancer Ther. 2012;11(6):1353‐1364. 22391039 10.1158/1535-7163.MCT-11-0915

[297] Hsu YL , Hou MF , Kuo PL , Huang YF , Tsai EM . Breast tumor‐associated osteoblast‐derived CXCL5 increases cancer progression by ERK/MSK1/Elk‐1/snail signaling pathway. Oncogene. 2013;32(37):4436‐4447. 23045282 10.1038/onc.2012.444

[298] Kuo PL , Huang MS , Hung JY , et al. Synergistic effect of lung tumor‐associated dendritic cell‐derived HB‐EGF and CXCL5 on cancer progression. Int J Cancer. 2014;135(1):96‐108. 24346967 10.1002/ijc.28673

[299] Li Q , Sun J , Cao Y , et al. Bu‐Shen‐Fang‐Chuan formula attenuates T‐lymphocytes recruitment in the lung of rats with COPD through suppressing CXCL9/CXCL10/CXCL11‐CXCR3 axis. Biomed Pharmacother. 2020;123:109735. 31864210 10.1016/j.biopha.2019.109735

[300] Tokunaga R , Zhang W , Naseem M , et al. CXCL9, CXCL10, CXCL11/CXCR3 axis for immune activation ‐ A target for novel cancer therapy. Cancer Treat Rev. 2018;63:40‐47. 29207310 10.1016/j.ctrv.2017.11.007 PMC5801162

[301] Gorbachev AV , Kobayashi H , Kudo D , et al. CXC chemokine ligand 9/monokine induced by IFN‐gamma production by tumor cells is critical for T cell‐mediated suppression of cutaneous tumors. J Immunol. 2007;178(4):2278‐2286. 17277133 10.4049/jimmunol.178.4.2278

[302] Qian C , An H , Yu Y , Liu S , Cao X . TLR agonists induce regulatory dendritic cells to recruit Th1 cells via preferential IP‐10 secretion and inhibit Th1 proliferation. Blood. 2007;109(8):3308‐3315. 17170125 10.1182/blood-2006-08-040337

[303] Karin N . CXCR3 ligands in cancer and autoimmunity, chemoattraction of effector T cells, and beyond. Front Immunol. 2020;11:976. 32547545 10.3389/fimmu.2020.00976 PMC7274023

[304] Paust HJ , Riedel JH , Krebs CF , et al. CXCR3+ regulatory T cells control TH1 responses in crescentic GN. J Am Soc Nephrol. 2016;27(7):1933‐1942. 26534920 10.1681/ASN.2015020203 PMC4926966

[305] Schoenborn JR , Wilson CB . Regulation of interferon‐gamma during innate and adaptive immune responses. Adv Immunol. 2007;96:41‐101. 17981204 10.1016/S0065-2776(07)96002-2

[306] Humblin E , Kamphorst AO . CXCR3‐CXCL9: it's all in the tumor. Immunity. 2019;50(6):1347‐1349. 31216458 10.1016/j.immuni.2019.05.013

[307] Reschke R , Yu J , Flood B , Higgs EF , Hatogai K , Gajewski TF . Immune cell and tumor cell‐derived CXCL10 is indicative of immunotherapy response in metastatic melanoma. J Immunother Cancer. 2021;9(9):e003521. 10.1136/jitc-2021-003521 PMC8487215 34593622

[308] Wightman SC , Uppal A , Pitroda SP , et al. Oncogenic CXCL10 signalling drives metastasis development and poor clinical outcome. Br J Cancer. 2015;113(2):327‐335. 26042934 10.1038/bjc.2015.193 PMC4506383

[309] Bagheri H , Pourhanifeh MH , Derakhshan M , et al. CXCL‐10: a new candidate for melanoma therapy?. Cell Oncol (Dordr). 2020;43(3):353‐365. 32207043 10.1007/s13402-020-00501-z PMC12990686

[310] Bai M , Chen X , Ba YI . CXCL10/CXCR3 overexpression as a biomarker of poor prognosis in patients with stage II colorectal cancer. Mol Clin Oncol. 2016;4(1):23‐30. 26870351 10.3892/mco.2015.665 PMC4726926

[311] Gomes‐Santos IL , Amoozgar Z , Kumar AS , et al. Exercise training improves tumor control by increasing CD8(+) T‐cell infiltration via CXCR3 signaling and sensitizes breast cancer to immune checkpoint blockade. Cancer Immunol Res. 2021;9(7):765‐778. 33839688 10.1158/2326-6066.CIR-20-0499 PMC8295193

[312] Sridharan V , Margalit DN , Lynch SA , et al. Definitive chemoradiation alters the immunologic landscape and immune checkpoints in head and neck cancer. Br J Cancer. 2016;115(2):252‐260. 27380136 10.1038/bjc.2016.166 PMC4947695

[313] Mitsuhashi A , Kondoh K , Horikawa K , et al. Programmed death (PD)‐1/PD‐ligand 1 blockade mediates antiangiogenic effects by tumor‐derived CXCL10/11 as a potential predictive biomarker. Cancer Sci. 2021;112(12):4853‐4866. 34628702 10.1111/cas.15161 PMC8645761

[314] Bolomsky A , Schreder M , Hübl W , Zojer N , Hilbe W , Ludwig H . Monokine induced by interferon gamma (MIG/CXCL9) is an independent prognostic factor in newly diagnosed myeloma. Leuk Lymphoma. 2016;57(11):2516‐2525. 26999330 10.3109/10428194.2016.1151511

[315] Sato Y , Motoyama S , Nanjo H , et al. CXCL10 expression status is prognostic in patients with advanced thoracic esophageal squamous cell carcinoma. Ann Surg Oncol. 2016;23(3):936‐942. 26464192 10.1245/s10434-015-4909-1

[316] Chow MT , Ozga AJ , Servis RL , et al. Intratumoral activity of the CXCR3 chemokine system is required for the efficacy of anti‐PD‐1 therapy. Immunity. 2019;50(6):1498‐1512.e5. 31097342 10.1016/j.immuni.2019.04.010 PMC6527362

[317] Gudowska‐Sawczuk M , Kudelski J , Mroczko B . The role of chemokine receptor CXCR3 and its ligands in renal cell carcinoma. Int J Mol Sci. 2020;21(22):8582. 10.3390/ijms21228582 PMC7696621 33202536

[318] Jie X , Chen Y , Zhao Y , et al. Targeting KDM4C enhances CD8(+) T cell mediated antitumor immunity by activating chemokine CXCL10 transcription in lung cancer. J Immunother Cancer. 2022;10(2):e003716. 10.1136/jitc-2021-003716 PMC8819819 35121645

[319] Wang X , Lu XL , Zhao HY , Zhang FC , Jiang XB . A novel recombinant protein of IP10‐EGFRvIIIscFv and CD8(+) cytotoxic T lymphocytes synergistically inhibits the growth of implanted glioma in mice. Cancer Immunol Immunother. 2013;62(7):1261‐1272. 23640602 10.1007/s00262-013-1426-6 PMC11029612

[320] Liu Z , Ravindranathan R , Li J , Kalinski P , Guo ZS , Bartlett DL . CXCL11‐Armed oncolytic poxvirus elicits potent antitumor immunity and shows enhanced therapeutic efficacy. Oncoimmunology. 2016;5(3):e1091554. 27141352 10.1080/2162402X.2015.1091554 PMC4839379

[321] Cao Y , Jiao N , Sun T , et al. CXCL11 correlates with antitumor immunity and an improved prognosis in colon cancer. Front Cell Dev Biol. 2021;9:646252. 33777950 10.3389/fcell.2021.646252 PMC7991085

[322] Pradelli E , Karimdjee‐Soilihi B , Michiels JF , et al. Antagonism of chemokine receptor CXCR3 inhibits osteosarcoma metastasis to lungs. Int J Cancer. 2009;125(11):2586‐2594. 19544560 10.1002/ijc.24665 PMC2772145

[323] Cambien B , Karimdjee BF , Richard‐Fiardo P , et al. Organ‐specific inhibition of metastatic colon carcinoma by CXCR3 antagonism. Br J Cancer. 2009;100(11):1755‐1764. 19436305 10.1038/sj.bjc.6605078 PMC2695685

[324] Koper OM , Kamińska J , Sawicki K , Kemona H . CXCL9, CXCL10, CXCL11, and their receptor (CXCR3) in neuroinflammation and neurodegeneration. Adv Clin Exp Med. 2018;27(6):849‐856. 29893515 10.17219/acem/68846

[325] Zhou YQ , Liu DQ , Chen SP , et al. The role of CXCR3 in neurological diseases. Curr Neuropharmacol. 2019;17(2):142‐150. 29119926 10.2174/1570159X15666171109161140 PMC6343204

[326] Balashov KE , Rottman JB , Weiner HL , Hancock WW . CCR5(+) and CXCR3(+) T cells are increased in multiple sclerosis and their ligands MIP‐1alpha and IP‐10 are expressed in demyelinating brain lesions. Proc Natl Acad Sci USA. 1999;96(12):6873‐6878. 10359806 10.1073/pnas.96.12.6873 PMC22009

[327] Narumi S , Kaburaki T , Yoneyama H , Iwamura H , Kobayashi Y , Matsushima K . Neutralization of IFN‐inducible protein 10/CXCL10 exacerbates experimental autoimmune encephalomyelitis. Eur J Immunol. 2002;32(6):1784‐1791. 12115662 10.1002/1521-4141(200206)32:6<1784::AID-IMMU1784>3.0.CO;2-R

[328] Chung CY , Liao F . CXCR3 signaling in glial cells ameliorates experimental autoimmune encephalomyelitis by restraining the generation of a pro‐Th17 cytokine milieu and reducing CNS‐infiltrating Th17 cells. J Neuroinflammation. 2016;13(1):76. 27068264 10.1186/s12974-016-0536-4 PMC4828793

[329] Piotrowska A , Ciapała K , Pawlik K , Kwiatkowski K , Rojewska E , Mika J . Comparison of the effects of chemokine receptors CXCR2 and CXCR3 pharmacological modulation in neuropathic pain model‐in vivo and in vitro study. Int J Mol Sci. 2021;22(20):11074. 10.3390/ijms222011074 PMC8538855 34681732

[330] Shono K , Yamaguchi I , Mizobuchi Y , et al. Downregulation of the CCL2/CCR2 and CXCL10/CXCR3 axes contributes to antitumor effects in a mouse model of malignant glioma. Sci Rep. 2020;10(1):15286. 32943658 10.1038/s41598-020-71857-3 PMC7499211

[331] Sharma I , Siraj F , Sharma KC , Singh A . Immunohistochemical expression of chemokine receptor CXCR3 and its ligand CXCL10 in low‐grade astrocytomas and glioblastoma multiforme: a tissue microarray‐based comparison. J Cancer Res Ther. 2016;12(2):793‐797. 27461653 10.4103/0973-1482.153657

[332] Liu C , Luo D , Reynolds BA , et al. Chemokine receptor CXCR3 promotes growth of glioma. Carcinogenesis. 2011;32(2):129‐137. 21051441 10.1093/carcin/bgq224 PMC3026840

[333] Xia MQ , Bacskai BJ , Knowles RB , Qin SX , Hyman BT . Expression of the chemokine receptor CXCR3 on neurons and the elevated expression of its ligand IP‐10 in reactive astrocytes: in vitro ERK1/2 activation and role in Alzheimer's disease. J Neuroimmunol. 2000;108(1‐2):227‐235. 10900358 10.1016/s0165-5728(00)00285-x

[334] Krauthausen M , Kummer MP , Zimmermann J , et al. CXCR3 promotes plaque formation and behavioral deficits in an Alzheimer's disease model. J Clin Invest. 2015;125(1):365‐378. 25500888 10.1172/JCI66771 PMC4382235

[335] Zhou Y , Cao H‐B , Li W‐J , Zhao L . The CXCL12 (SDF‐1)/CXCR4 chemokine axis: oncogenic properties, molecular targeting, and synthetic and natural product CXCR4 inhibitors for cancer therapy. Chin J Natural Med. 2018;16(11):801‐810. 10.1016/S1875-5364(18)30122-5 30502762

[336] Mousavi A . CXCL12/CXCR4 signal transduction in diseases and its molecular approaches in targeted‐therapy. Immunol Lett. 2020;217:91‐115. 31747563 10.1016/j.imlet.2019.11.007

[337] Huynh C , Dingemanse J , Sidharta PN . Relevance of the CXCR4/CXCR7‐CXCL12 axis and its effect in pathophysiological conditions. Pharmacol Res. 2020;161:105092. 32758634 10.1016/j.phrs.2020.105092

[338] Bianchi ME , Mezzapelle R . The chemokine receptor CXCR4 in cell proliferation and tissue regeneration. Front Immunol. 2020;11:2109. 32983169 10.3389/fimmu.2020.02109 PMC7484992

[339] Wang M , Wang L , Ren T , Xu L , Wen Z . IL‐17A/IL‐17RA interaction promoted metastasis of osteosarcoma cells. Cancer Biol Ther. 2013;14(2):155‐163. 23192273 10.4161/cbt.22955 PMC3571997

[340] Ao M , Franco OE , Park D , Raman D , Williams K , Hayward SW . Cross‐talk between paracrine‐acting cytokine and chemokine pathways promotes malignancy in benign human prostatic epithelium. Cancer Res. 2007;67(9):4244‐4253. 17483336 10.1158/0008-5472.CAN-06-3946

[341] Lounsbury N . Advances in CXCR7 modulators. Pharmaceuticals (Basel). 2020;13(2):33. 10.3390/ph13020033 PMC7169404 32098047

[342] Cho BS , Kim HJ , Konopleva M . Targeting the CXCL12/CXCR4 axis in acute myeloid leukemia: from bench to bedside. Korean J Intern Med. 2017;32(2):248‐257. 28219003 10.3904/kjim.2016.244 PMC5339474

[343] Liu C , Weng Y , Yuan T , et al. CXCL12/CXCR4 signal axis plays an important role in mediating bone morphogenetic protein 9‐induced osteogenic differentiation of mesenchymal stem cells. Int J Med Sci. 2013;10(9):1181‐1192. 23935395 10.7150/ijms.6657 PMC3739017

[344] Décaillot FM , Kazmi MA , Lin Y , Ray‐Saha S , Sakmar TP , Sachdev P . CXCR7/CXCR4 heterodimer constitutively recruits beta‐arrestin to enhance cell migration. J Biol Chem. 2011;286(37):32188‐32197. 21730065 10.1074/jbc.M111.277038 PMC3173186

[345] Ceholski DK , Turnbull IC , Pothula V , et al. CXCR4 and CXCR7 play distinct roles in cardiac lineage specification and pharmacologic β‐adrenergic response. Stem Cell Res. 2017;23:77‐86. 28711757 10.1016/j.scr.2017.06.015 PMC5859259

[346] Shi Y , Riese DJ , Shen J . The role of the CXCL12/CXCR4/CXCR7 chemokine axis in cancer. Front Pharmacol. 2020;11:574667. 33363463 10.3389/fphar.2020.574667 PMC7753359

[347] Santagata S , Ieranò C , Trotta AM , et al. CXCR4 and CXCR7 signaling pathways: a focus on the cross‐talk between cancer cells and tumor microenvironment. Front Oncol. 2021;11:591386. 33937018 10.3389/fonc.2021.591386 PMC8082172

[348] Sun X , Cheng G , Hao M , et al. CXCL12/CXCR4/CXCR7 chemokine axis and cancer progression. Cancer Metastasis Rev. 2010;29(4):709‐722. 20839032 10.1007/s10555-010-9256-x PMC3175097

[349] Mitchell B , Mahalingam M . The CXCR4/CXCL12 axis in cutaneous malignancies with an emphasis on melanoma. Histol Histopathol. 2014;29(12):1539‐1546. 24879309 10.14670/HH-29.1539

[350] Chen L , Zhu M , Yu S , et al. Arg kinase mediates CXCL12/CXCR4‐induced invadopodia formation and invasion of glioma cells. Exp Cell Res. 2020;389(1):111893. 32035133 10.1016/j.yexcr.2020.111893

[351] Wang M , Lin T , Wang Y , et al. CXCL12 suppresses cisplatin‐induced apoptosis through activation of JAK2/STAT3 signaling in human non‐small‐cell lung cancer cells. Onco Targets Ther. 2017;10:3215‐3224. 28721072 10.2147/OTT.S133055 PMC5499863

[352] Daniel SK , Seo YD , Pillarisetty VG . The CXCL12‐CXCR4/CXCR7 axis as a mechanism of immune resistance in gastrointestinal malignancies. Semin Cancer Biol. 2020;65:176‐188. 31874281 10.1016/j.semcancer.2019.12.007

[353] Malik S , Westcott JM , Brekken RA , Burrows FJ . CXCL12 in pancreatic cancer: its function and potential as a therapeutic drug target. Cancers (Basel). 2021;14(1):86. 10.3390/cancers14010086 PMC8750050 35008248

[354] Khare T , Bissonnette M , Khare S . CXCL12‐CXCR4/CXCR7 axis in colorectal cancer: therapeutic target in preclinical and clinical studies. Int J Mol Sci. 2021;22(14):7371. 10.3390/ijms22147371 PMC8305488 34298991

[355] Yang P , Wang G , Huo H , Li Q , Zhao Y , Liu Y . SDF‐1/CXCR4 signaling up‐regulates survivin to regulate human sacral chondrosarcoma cell cycle and epithelial‐mesenchymal transition via ERK and PI3K/AKT pathway. Med Oncol. 2015;32(1):377. 25428386 10.1007/s12032-014-0377-x

[356] Duan Y , Zhang S , Wang L , et al. Targeted silencing of CXCR4 inhibits epithelial‐mesenchymal transition in oral squamous cell carcinoma. Oncol Lett. 2016;12(3):2055‐2061. 27602138 10.3892/ol.2016.4838 PMC4998519

[357] Yao C , Li P , Song H , et al. CXCL12/CXCR4 axis upregulates twist to induce EMT in human glioblastoma. Mol Neurobiol. 2016;53(6):3948‐3953. 26179613 10.1007/s12035-015-9340-x

[358] Mortezaee K . CXCL12/CXCR4 axis in the microenvironment of solid tumors: a critical mediator of metastasis. Life Sci. 2020;249:117534. 32156548 10.1016/j.lfs.2020.117534

[359] Zhou W , Guo S , Liu M , Burow ME , Wang G . Targeting CXCL12/CXCR4 axis in tumor immunotherapy. Curr Med Chem. 2019;26(17):3026‐3041. 28875842 10.2174/0929867324666170830111531 PMC5949083

[360] Hirbe AC , Morgan EA , Weilbaecher KN . The CXCR4/SDF‐1 chemokine axis: a potential therapeutic target for bone metastases?. Curr Pharm Des. 2010;16(11):1284‐1290. 20166978 10.2174/138161210791034012

[361] Jiang C , Ma S , Hu R , et al. Effect of CXCR4 on apoptosis in osteosarcoma cells via the PI3K/Akt/NF‐κβ signaling pathway. Cell Physiol Biochem. 2018;46(6):2250‐2260. 29734183 10.1159/000489593

[362] Mirandola L , Apicella L , Colombo M , et al. Anti‐Notch treatment prevents multiple myeloma cells localization to the bone marrow via the chemokine system CXCR4/SDF‐1. Leukemia. 2013;27(7):1558‐1566. 23354012 10.1038/leu.2013.27

[363] Guo S , Xiao D , Liu H , Zheng X , Liu L , Liu S . Interfering with CXCR4 expression inhibits proliferation, adhesion and migration of breast cancer MDA‐MB‐231 cells. Oncol Lett. 2014;8(4):1557‐1562. 25202367 10.3892/ol.2014.2323 PMC4156168

[364] Tan XY , Chang S , Liu W , Tang HH . Silencing of CXCR4 inhibits tumor cell proliferation and neural invasion in human hilar cholangiocarcinoma. Gut Liver. 2014;8(2):196‐204. 24672662 10.5009/gnl.2014.8.2.196 PMC3964271

[365] Luo HN , Wang ZH , Sheng Y , et al. MiR‐139 targets CXCR4 and inhibits the proliferation and metastasis of laryngeal squamous carcinoma cells. Med Oncol. 2014;31(1):789. 24318902 10.1007/s12032-013-0789-z

[366] Wang DF , Lou N , Qiu MZ , Lin YB , Liang Y . Effects of CXCR4 gene silencing by lentivirus shRNA on proliferation of the EC9706 human esophageal carcinoma cell line. Tumour Biol. 2013;34(5):2951‐2959. 23744460 10.1007/s13277-013-0858-0

[367] Long P , Sun F , Ma Y , Huang Y . Inhibition of CXCR4 and CXCR7 for reduction of cell proliferation and invasion in human endometrial cancer. Tumour Biol. 2016;37(6):7473‐7480. 26678890 10.1007/s13277-015-4580-y

[368] Saha A , Ahn S , Blando J , Su F , Kolonin MG , DiGiovanni J . Proinflammatory CXCL12‐CXCR4/CXCR7 signaling axis drives Myc‐induced prostate cancer in obese mice. Cancer Res. 2017;77(18):5158‐5168. 28687617 10.1158/0008-5472.CAN-17-0284 PMC5600849

[369] Luo Y , Li Q , Yang X , et al. Overexpression of CXCR7 is a novel indicator for enzalutamide resistance in castration‐resistant prostate cancer patients. Dis Markers. 2021;2021:6649579. 34413914 10.1155/2021/6649579 PMC8369184

[370] Yang J , Tang H , Huang J , An H . Upregulation of CXCR7 is associated with poor prognosis of prostate cancer. Med Sci Monit. 2018;24:5185‐5191. 30047547 10.12659/MSM.906180 PMC6074061

[371] Deng L , Zheng W , Dong X , et al. Chemokine receptor CXCR7 is an independent prognostic biomarker in glioblastoma. Cancer Biomark. 2017;20(1):1‐6. 28759950 10.3233/CBM-151430

[372] Li S , Fong KW , Gritsina G , et al. Activation of MAPK signaling by CXCR7 leads to enzalutamide resistance in prostate cancer. Cancer Res. 2019;79(10):2580‐2592. 30952632 10.1158/0008-5472.CAN-18-2812 PMC6522281

[373] Qin HJ , Xu T , Wu HT , et al. SDF‐1/CXCR4 axis coordinates crosstalk between subchondral bone and articular cartilage in osteoarthritis pathogenesis. Bone. 2019;125:140‐150. 31108241 10.1016/j.bone.2019.05.010

[374] Murad HAS , Rafeeq MM , Alqurashi TMA . Role and implications of the CXCL12/CXCR4/CXCR7 axis in atherosclerosis: still a debate. Ann Med. 2021;53(1):1598‐1612. 34494495 10.1080/07853890.2021.1974084 PMC8439212

[375] Gao JH , He LH , Yu XH , et al. CXCL12 promotes atherosclerosis by downregulating ABCA1 expression via the CXCR4/GSK3β/β‐catenin(T120)/TCF21 pathway. J Lipid Res. 2019;60(12):2020‐2033. 31662443 10.1194/jlr.RA119000100 PMC6889714

[376] Tian Y , Yin H , Deng X , Tang B , Ren X , Jiang T . CXCL12 induces migration of oligodendrocyte precursor cells through the CXCR4‑activated MEK/ERK and PI3K/AKT pathways. Mol Med Rep. 2018;18(5):4374‐4380. 30221695 10.3892/mmr.2018.9444 PMC6172403

[377] Song ZY , Wang F , Cui SX , Qu XJ . Knockdown of CXCR4 inhibits CXCL12‐induced angiogenesis in HUVECs through downregulation of the MAPK/ERK and PI3K/AKT and the Wnt/β‐catenin pathways. Cancer Invest. 2018;36(1):10‐18. 29381400 10.1080/07357907.2017.1422512

[378] Hao H , Hu S , Chen H , et al. Loss of endothelial CXCR7 impairs vascular homeostasis and cardiac remodeling after myocardial infarction: implications for cardiovascular drug discovery. Circulation. 2017;135(13):1253‐1264. 28154007 10.1161/CIRCULATIONAHA.116.023027

[379] Cheng X , Wang H , Zhang X , et al. The role of SDF‐1/CXCR4/CXCR7 in neuronal regeneration after cerebral ischemia. Front Neurosci. 2017;11:590. 29123467 10.3389/fnins.2017.00590 PMC5662889

[380] Cruz‐Orengo L , Holman DW , Dorsey D , et al. CXCR7 influences leukocyte entry into the CNS parenchyma by controlling abluminal CXCL12 abundance during autoimmunity. J Exp Med. 2011;208(2):327‐339. 21300915 10.1084/jem.20102010 PMC3039853

[381] Gunn MD , Ngo VN , Ansel KM , Ekland EH , Cyster JG , Williams LT . A B‐cell‐homing chemokine made in lymphoid follicles activates Burkitt's lymphoma receptor‐1. Nature. 1998;391(6669):799‐803. 9486651 10.1038/35876

[382] Shi K , Hayashida K , Kaneko M , et al. Lymphoid chemokine B cell‐attracting chemokine‐1 (CXCL13) is expressed in germinal center of ectopic lymphoid follicles within the synovium of chronic arthritis patients. J Immunol. 2001;166(1):650‐655. 11123349 10.4049/jimmunol.166.1.650

[383] Rubio AJ , Porter T , Zhong X . Duality of B cell‐CXCL13 axis in tumor immunology. Front Immunol. 2020;11:521110. 33193299 10.3389/fimmu.2020.521110 PMC7609404

[384] Kazanietz MG , Durando M , Cooke M . CXCL13 and its receptor CXCR5 in cancer: inflammation, immune response, and beyond. Front Endocrinol (Lausanne). 2019;10:471. 31354634 10.3389/fendo.2019.00471 PMC6639976

[385] Wan S , Lin M , Mao Y , Chen X , Liang D . Altered expression of CXCL13 and its chemokine receptor CXCR5 on B lymphocytes during active graves' orbitopathy. Curr Eye Res. 2021;46(2):210‐216. 32643429 10.1080/02713683.2020.1786132

[386] Hussain M , Liu J , Wang GZ , Zhou GB . CXCL13 signaling in the tumor microenvironment. Adv Exp Med Biol. 2021;1302:71‐90. 34286442 10.1007/978-3-030-62658-7_6

[387] Tian C , Li C , Zeng Y , et al. Identification of CXCL13/CXCR5 axis's crucial and complex effect in human lung adenocarcinoma. Int Immunopharmacol. 2021;94:107416. 33676174 10.1016/j.intimp.2021.107416

[388] Chao CC , Lee WF , Wang SW , et al. CXC chemokine ligand‐13 promotes metastasis via CXCR5‐dependent signaling pathway in non‐small cell lung cancer. J Cell Mol Med. 2021;25(19):9128‐9140. 34427969 10.1111/jcmm.16743 PMC8500967

[389] Siliņa K , Soltermann A , Attar FM , et al. Germinal centers determine the prognostic relevance of tertiary lymphoid structures and are impaired by corticosteroids in lung squamous cell carcinoma. Cancer Res. 2018;78(5):1308‐1320. 29279354 10.1158/0008-5472.CAN-17-1987

[390] Thommen DS , Koelzer VH , Herzig P , et al. A transcriptionally and functionally distinct PD‐1(+) CD8(+) T cell pool with predictive potential in non‐small‐cell lung cancer treated with PD‐1 blockade. Nat Med. 2018;24(7):994‐1004. 29892065 10.1038/s41591-018-0057-z PMC6110381

[391] Panse J , Friedrichs K , Marx A , et al. Chemokine CXCL13 is overexpressed in the tumour tissue and in the peripheral blood of breast cancer patients. Br J Cancer. 2008;99(6):930‐938. 18781150 10.1038/sj.bjc.6604621 PMC2538749

[392] Jiang L , Wang D , Sheng M , et al. CXCL13/CXCR5 are potential biomarkers for diagnosis and prognosis for breast cancer. J BUON. 2020;25(6):2552‐2561. 33455096

[393] Heimes AS , Madjar K , Edlund K , et al. Subtype‐specific prognostic impact of different immune signatures in node‐negative breast cancer. Breast Cancer Res Treat. 2017;165(2):293‐300. 28585074 10.1007/s10549-017-4327-0

[394] Schmidt M , Weyer‐Elberich V , Hengstler JG , et al. Prognostic impact of CD4‐positive T cell subsets in early breast cancer: a study based on the FinHer trial patient population. Breast Cancer Res. 2018;20(1):15. 29482642 10.1186/s13058-018-0942-x PMC5827982

[395] Ma JJ , Jiang L , Tong DY , Ren YN , Sheng MF , Liu HC . CXCL13 inhibition induce the apoptosis of MDA‐MB‐231 breast cancer cells through blocking CXCR5/ERK signaling pathway. Eur Rev Med Pharmacol Sci. 2018;22(24):8755‐8762. 30575916 10.26355/eurrev_201812_16641

[396] Xu L , Liang Z , Li S , Ma J . Signaling via the CXCR5/ERK pathway is mediated by CXCL13 in mice with breast cancer. Oncol Lett. 2018;15(6):9293‐9298. 29844827 10.3892/ol.2018.8510 PMC5958818

[397] Yang M , Lu J , Zhang G , et al. CXCL13 shapes immunoactive tumor microenvironment and enhances the efficacy of PD‐1 checkpoint blockade in high‐grade serous ovarian cancer. J Immunother Cancer. 2021;9(1):e001136. 10.1136/jitc-2020-001136 PMC7813306 33452206

[398] Zhu Z , Zhang X , Guo H , Fu L , Pan G , Sun Y . CXCL13‐CXCR5 axis promotes the growth and invasion of colon cancer cells via PI3K/AKT pathway. Mol Cell Biochem. 2015;400(1‐2):287‐295. 25476740 10.1007/s11010-014-2285-y

[399] Ohandjo AQ , Liu Z , Dammer EB , et al. Transcriptome network analysis identifies CXCL13‐CXCR5 signaling modules in the prostate tumor immune microenvironment. Sci Rep. 2019;9(1):14963. 31628349 10.1038/s41598-019-46491-3 PMC6802083

[400] Si Z , Hu H . Identification of CXCL13 as an immune‐related biomarker associated with tumorigenesis and prognosis in cutaneous melanoma patients. Med Sci Monit. 2021;27:e932052. 34247183 10.12659/MSM.932052 PMC8280950

[401] Zheng Z , Cai Y , Chen H , et al. CXCL13/CXCR5 axis predicts poor prognosis and promotes progression through PI3K/AKT/mTOR pathway in clear cell renal cell carcinoma. Front Oncol. 2018;8:682. 30723697 10.3389/fonc.2018.00682 PMC6349755

[402] Li JP , Wu CY , Chen MY , et al. PD‐1(+)CXCR5(−)CD4(+) Th‐CXCL13 cell subset drives B cells into tertiary lymphoid structures of nasopharyngeal carcinoma. J Immunother Cancer. 2021;9(7):e002101. 10.1136/jitc-2020-002101 PMC8276302 34253636

[403] Tsai CH , Chen CJ , Gong CL , et al. CXCL13/CXCR5 axis facilitates endothelial progenitor cell homing and angiogenesis during rheumatoid arthritis progression. Cell Death Dis. 2021;12(9):846. 34518512 10.1038/s41419-021-04136-2 PMC8437941

[404] Weiss JM , Robinet M , Aricha R , et al. Novel CXCL13 transgenic mouse: inflammation drives pathogenic effect of CXCL13 in experimental myasthenia gravis. Oncotarget. 2016;7(7):7550‐7562. 26771137 10.18632/oncotarget.6885 PMC4884937

[405] Aqrawi LA , Ivanchenko M , Björk A , et al. Diminished CXCR5 expression in peripheral blood of patients with Sjögren's syndrome may relate to both genotype and salivary gland homing. Clin Exp Immunol. 2018;192(3):259‐270. 29453859 10.1111/cei.13118 PMC5980494

[406] Bao YQ , Wang JP , Dai ZW , et al. Increased circulating CXCL13 levels in systemic lupus erythematosus and rheumatoid arthritis: a meta‐analysis. Clin Rheumatol. 2020;39(1):281‐290. 31523787 10.1007/s10067-019-04775-z

[407] Moser B . CXCR5, the defining marker for follicular B helper T (TFH) cells. Front Immunol. 2015;6:296. 26106395 10.3389/fimmu.2015.00296 PMC4459225

[408] Mehraj V , Ramendra R , Isnard S , et al. CXCL13 as a biomarker of immune activation during early and chronic HIV infection. Front Immunol. 2019;10:289. 30846990 10.3389/fimmu.2019.00289 PMC6393370

[409] Bekele Feyissa Y , Chiodi F , Sui Y , Berzofsky JA . The role of CXCL13 in antibody responses to HIV‐1 infection and vaccination. Front Immunol. 2021;12:638872. 33732259 10.3389/fimmu.2021.638872 PMC7959754

[410] Widney DP , Breen EC , Boscardin WJ , et al. Serum levels of the homeostatic B cell chemokine, CXCL13, are elevated during HIV infection. J Interferon Cytokine Res. 2005;25(11):702‐706. 16318584 10.1089/jir.2005.25.702

[411] Li Y , Tang L , Guo L , et al. CXCL13‐mediated recruitment of intrahepatic CXCR5(+)CD8(+) T cells favors viral control in chronic HBV infection. J Hepatol. 2020;72(3):420‐430. 31610223 10.1016/j.jhep.2019.09.031

[412] Schmidt C , Plate A , Angele B , et al. A prospective study on the role of CXCL13 in Lyme neuroborreliosis. Neurology. 2011;76(12):1051‐1058. 21422457 10.1212/WNL.0b013e318211c39a

[413] Yu Q , Cheng Y , Wang Y , et al. Aberrant humoral immune responses in neurosyphilis: cXCL13/CXCR5 play a pivotal role for B‐cell recruitment to the cerebrospinal fluid. J Infect Dis. 2017;216(5):534‐544. 28931218 10.1093/infdis/jix233

